# Detection of lineage-specific evolutionary changes among primate species

**DOI:** 10.1186/1471-2105-12-274

**Published:** 2011-07-04

**Authors:** Mihaela Pertea, Geo M Pertea, Steven L Salzberg

**Affiliations:** 1 Center for Bioinformatics and Computational Biology, University of Maryland, College Park, Maryland, USA; 2 McKusick-Nathans Institute of Genetic Medicine, Johns Hopkins University School of Medicine, Baltimore, Maryland, USA

## Abstract

**Background:**

Comparison of the human genome with other primates offers the opportunity to detect evolutionary events that created the diverse phenotypes among the primate species. Because the primate genomes are highly similar to one another, methods developed for analysis of more divergent species do not always detect signs of evolutionary selection.

**Results:**

We have developed a new method, called DivE, specifically designed to find regions that have evolved either more or less rapidly than expected, for any clade within a set of very closely related species. Unlike some previous methods, DivE does not rely on rates of synonymous and nonsynonymous substitution, which enables it to detect evolutionary events in noncoding regions. We demonstrate using simulated data that DivE compares favorably to alternative methods, and we then apply DivE to the ENCODE regions in 14 primate species. We identify thousands of regions in these primates, ranging from 50 to >10000 bp in length, that appear to have experienced either constrained or accelerated rates of evolution. In particular, we detected 4942 regions that have potentially undergone positive selection in one or more primate species. Most of these regions occur outside of protein-coding genes, although we identified 20 proteins that have experienced positive selection.

**Conclusions:**

DivE provides an easy-to-use method to predict both positive and negative selection in noncoding DNA, that is particularly well-suited to detecting lineage-specific selection in large genomes.

## Background

The genome of a living species is the product of a long series of changes, including neutral, beneficial, and detrimental alterations to the sequence. Sequence changes that affect the organism's fitness are subject to evolutionary pressures, such as the pressure to survive, to out-compete other species, and to defend the organism against external attack. In order to uncover these changes, we need to know what the ancestral genome looked like, which we can infer by comparing multiple genomes to one another. As we accumulate genomes from species related to human, and especially from within the primate lineages, we should be able to learn more about what makes humans special. At the same time, we can learn what makes each primate different from the others. Until recently, methods for detecting the effects of evolution had been designed for relatively distant species such as humans and mice. With the publication of the chimpanzee genome [1], we had our first look at a very close relative of human. The genomes of chimpanzees and humans are so close, in fact, that sequence similarity cannot be used to infer functional significance: in most cases, similarity simply reflects the recent divergence between the species. With more species, sequence comparison even among close relatives can be used to tease apart regions that are constrained by evolutionary forces and that, consequently, are likely to have functional importance to the biology of humans.

Recently, the ENCODE project selected 13 primates (in addition to human) and sequenced 1% of each genome to produce "comparative grade" [2] assemblies. These high-quality sequences from close human relatives give us a greater ability than before to detect the signs of evolutionary selection on the human genome and other primates. The traces of evolution's effects can be found more easily when they are shared among multiple species. Signs of selection also may indicate functionally important sequences, and in particular they can be used to identify regulatory regions that fall outside protein-coding regions and are otherwise difficult to find.

Broadly speaking, there are two main types of selective processes driving the evolution of genomes. Negative or purifying selection is the evolutionary pressure that eliminates deleterious mutations from a population. Most mutations in the genome are probably neutral, because most of the genome is itself non-functional, but within coding regions, the majority of mutations are deleterious [3]. Deleterious mutations are likely to be transient; i.e., they do not become fixed in the human population. Negative selection has been identified principally by pairwise sequence alignment methods, through which DNA or amino acid sequences can be shown to be more highly conserved than expected based on the overall evolutionary distance between a pair of species. By one well-known estimate, approximately 5% of the human genome is under negative selection [4], of which only 1.5% is contained in protein-coding exons.

Positive selection is more difficult to detect. In positive selection, a region of the genome, protein coding or otherwise, accumulates beneficial mutations that provide a survival advantage to the organism. One way to detect positive selection is by the presence of genes that have acquired many more mutations than other genes when compared to close relatives. A well-documented example of positive selection is the rapid change in the hemagglutinin protein on the surface of the human influenza virus, which is in constant competition with the human immune system [5]. Positive selection must be carefully distinguished from the relaxation of selective constraints, however. If a sequence (a gene or a regulatory sequence) ceases to perform its function, and if that function is no longer needed by the organism, then it might accumulate mutations faster precisely because it is no longer functional.

In this study, we describe a new method, called DivE, for detecting lineage-specific regions evolving at a slower or faster rate than the background evolutionary rate in the primate genomes. Other methods have been previously developed for detecting selection, but most look only at conservation of sequence (negative selection) in all aligned species, and are not lineage specific [6-12]. Methods to detect accelerated regions (i.e. regions evolving at faster-than-neutral rates) have also appeared recently [13-18]. Some of these methods allow for lineage-specific selection [14-16,18], but in contrast with conservation-detection methods, they cannot be easily used for genome-wide scans to detect selection, and look only at particular regions of interest. Although accelerated regions may indicate positive selection, this is not necessarily the case [19]. There are many examples where positive selection manifests itself at only a small number of sites [20-23]. Our method is not suited to the identification of positive selection in these cases.

Recently a new program, phyloP, was developed to examine the more general problem of detecting either conserved or accelerated regions in a set of aligned orthologous sequences from multiple species [24]. PhyloP implements four different statistical phylogenetic tests to find significant departures from non-neutral substitution rates on a whole phylogeny as well as on selected subtrees (clades) of interest in the phylogeny. It was shown to have fairly good accuracy in detecting strong selection even at individual nucleotides. In one respect, DivE is similar to phyloP in that both methods try to solve the general problem of detecting an increase or a decrease in the rate of substitution in a given genomic region, either on a whole phylogeny or within a clade of the phylogeny.

However, in phyloP the phylogenetic subtree of interest needs to be provided to the program, while in contrast DivE addresses the more complicated problem in which the lineage of interest is not pre-specified. Therefore the lineage under selection must be detected automatically by DivE from among all possible subtrees within a phylogeny. Another significant difference is that applying phyloP to an entire genome to detect selection involves using a sliding window approach. Although a sliding-window analysis is a popular method to test for negative or positive selection, there are results that show that this approach is not generally valid if selective trends are not known *a priori *in a given region [25]. In addition, the sensitivity of phyloP is dependent on the size of the window used to scan the genome, which in turn depends on the number of species available. DivE doesn't use a sliding window approach, but instead tries to determine the optimal size for the selected genomic element that is predicted to be under selection. In regard to these differences, DivE is more similar to DLESS [26], a method that detects sequences that have either come under selection, or begun to drift, in any lineage. While DLESS only allows for detection of a "gain" event (conservation in a phylogenetic subtree) or a "loss" event (where a subtree is evolving neutrally while the rest of the tree is conserved), DivE also detects acceleration events in any clade of the tree. DLESS is the only other computational method, prior to DivE, that can detect lineage-specific selection when the lineage of interest is not pre-specified.

Below we present our method for detecting both conserved and accelerated regions and apply it to 14 primate genomes. We describe results on simulated and real data, including the identification of positively selected genes that intersect regions evolving faster than the neutral mutation rate. The method described in this paper is implemented in the DivE package which is available as free, open-source software [27].

## Results

### Simulation results

For our simulation tests, we created sequence elements that were both positively and negatively selected within the same 14 primate species used for our later experiments on real data. Because we knew the precise location, size, and type of selection involved in each element, we could use this data to evaluated the accuracy of DivE and compare it to other methods.

**Figure 1:**
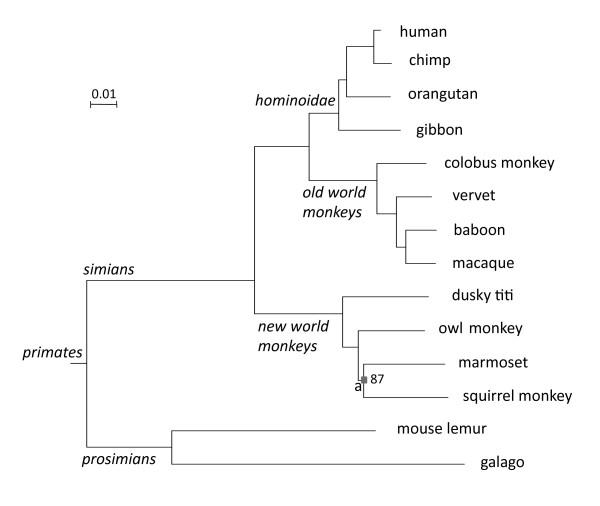
**Phylognetic tree of the fourteen primates used in this study**. The branches in the tree are proportional to the expected number of substitutions per site. For reference, a scale of 0.01 substitutions per site is shown. The names of the major clades in this phylogeny are shown in italics. All nodes have 100% support values in a Bayesian phylogeny framework, except for node a.

**Figure 2:**
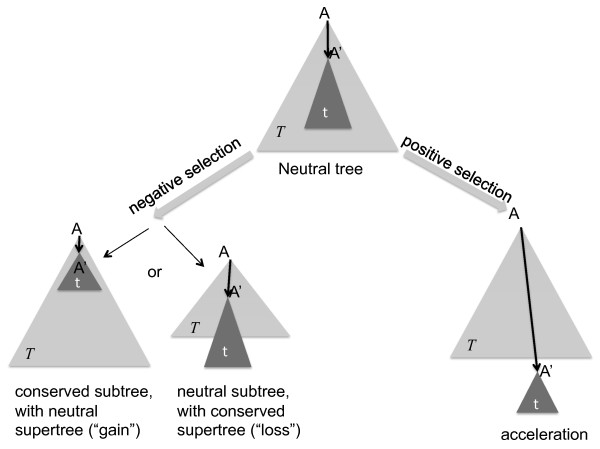
**The selection problem**. Branch length changes to the neutral phylogenetic tree are shown for each type of selection considered in this study: gain, loss, and acceleration. The figure shows a phylogenetic tree composed of a supertree ***T ***with root **A**, containing a subtree **t **with root **A' **that is affected by selection. The left side of the figure shows the effects of negative selection on the relative shape and position of **t**. In the case of "gain" elements the branches within **t **as well as the branch from **A **to **A' **are reduced in length. In the case of "loss" elements, **t **is unaffected while the rest of the tree is reduced in size (conserved). Both gain and loss events are particular cases of negative selection affecting different lineages in the tree. In the case of acceleration, shown on the right side of the figure, only the branch from **A **to **A' **increases in length. Positive selection is one cause, but not the only cause, of this type of increase. Branches within **t **may decrease in length if there is additional selection within the tree.

We created simulated data sets that contain selected elements of lengths between 50 bp and 1000 bp in all subtrees of the phylogeny of the 14 primates (see Figure 1 and Methods for a description of the primate phylogeny). Conserved elements are either "gained" or "lost" on a particular lineage, where a "gain" event implies that the region defined by that particular lineage will experience selective pressure that will tend to eliminate individuals with mutations in that region (i.e., negative or purifying selection). A "loss" event implies that the region in question does not have evolutionary constraints, and will evolve at the neutral substitution rate, while the rest of the tree is constrained. The average substitution rate observed for conserved elements is a fraction of the non-conserved regions, and we therefore can simulate negative selection by reducing the branch lengths of the selected subtree (for gain) or supertree (for loss), as depicted in Figure 2. For accelerated elements, the observed substitution rate is greater than the neutral rate. A special case of accelerated evolution is positive selection, which occurs when a sequence is under pressure to change more rapidly; e.g., in order to adapt to changes in the environment. A particular subtree might be under positive selection if the branch from the whole tree leading to that subtree is elongated, while the branches within the subtree are the same or shorter than the background mutation rate (see Figure 2).

In our simulation, the accelerated elements in a given subtree are generated according to a phylogeny in which the parent edge of the node at the root of the subtree is longer. We chose a scaling parameter *ρ *(where *ρ *∈ {0.01, 0.02, 0.05, 0.1, 0.2, 0.3, 0.4, 0.5}) to represent the selection strength affecting the elements by reducing (through multiplication by *ρ*) or enlarging (by dividing by *ρ*) different branches of the phylogenetic tree depending on the type of section [28] as described above. We obtained the results presented here by generating 100 elements for each subtree in the phylogeny, type of selection, selection strength, and length.

We used DivE to detect regions that have either come under negative selection, or begun to drift on any lineage. We compared its results to DLESS [26], the only previous method to our knowledge that has been developed to solve the same problem as the one presented here. The same input was given to both DivE and DLESS: a set of aligned orthologous sequences and a neutral phylogenetic model generated with phyloFit [29], which included a substitution rate matrix, a tree with branch lengths in units of expected substitutions per site, and estimates of nucleotide equilibrium frequencies.

Table 1 shows the average accuracies obtained by DivE and DLESS for the prediction of elements of different lengths and at different selection strengths (as specified by the scaling parameter *ρ*) that are either gained or lost. Averages are computed on all clades in the phylogeny (excluding the whole tree). The accuracy of prediction for each program is computed as the average of the precision and recall to detect sites under selection. Precision and recall performances for detecting negative selection in all subtrees for both DivE and DLESS are shown in Additional file 1, Tables S1-S16.

**Table 1:** Average accuracy (shown as a percentage) of DivE (dE) and DLESS (dl) to detect conservation and accelerated evolution computed on all primate subtrees for simulated data of different lengths.

		50 bp		100 bp		200 bp		500 bp		1000 bp	
		dE	dl	dE	dl	dE	dl	dE	dl	dE	dl
gain	0.01	**4.4**	1.6	**15.0**	5.3	**32.3**	15.6	**59.6**	40.0	**80.7**	66.0
	
	0.02	**5.2**	1.7	**15.1**	6.3	**32.7**	15.9	**58.7**	40.0	**81.5**	63.7
	
	0.05	**3.8**	1.6	**13.0**	5.6	**30.5**	15.6	**56.0**	39.9	**79.6**	62.2
	
	0.1	**4.7**	0.9	**12.2**	3.8	**27.5**	14.6	**54.0**	37.8	**77.2**	58.6
	
	0.2	**2.0**	1.6	**8.7**	2.7	**22.7**	10.5	**48.6**	33.6	**70.7**	53.8
	
	0.3	**2.1**	0.2	**6.6**	2.0	**18.3**	8.9	**40.7**	28.0	**62.1**	48.4
	
	0.4	**1.3**	0.3	**5.2**	1.8	**13.6**	5.8	**33.8**	21.5	**54.2**	36.6
	
	0.5	0	**0.2**	**1.9**	0.1	**13.0**	4.1	**25.4**	13.8	**45.4**	26.0

loss	0.01	**85.6**	73.6	**93.9**	86.5	**97.4**	93.5	**99.1**	97.2	**99.5**	98.8
	
	0.02	**85.7**	72.7	**93.7**	86.8	**97.3**	93.5	**99.0**	97.3	**99.5**	98.6
	
	0.05	**84.3**	69.3	**93.4**	86.9	**97.2**	93.6	**99.0**	97.3	**99.5**	98.7
	
	0.1	**82.2**	61.5	**91.8**	86.3	**96.7**	93.1	**98.9**	97.4	**99.5**	98.9
	
	0.2	**70.6**	43.9	**87.5**	84.1	**94.2**	91.9	**98.1**	97.3	**99.2**	98.7
	
	0.3	**53.9**	29.8	**81.6**	72.3	**90.5**	90.4	96.2	**96.7**	98.3	98.3
	
	0.4	**41.2**	22.8	**70.7**	52.6	**85.7**	80.7	93.7	**94.0**	**96.7**	96.6
	
	0.5	**31.3**	12.5	**55.1**	37.0	**77.3**	58.9	**89.8**	80.8	**94.4**	87.6

acc	0.01^-1^	71.0	-	80.4	-	88.0	-	93.6	-	96.4	-
	
	0.02^-1^	69.5	-	79.8	-	87.1	-	93.5	-	96.1	-
	
	0.05^-1^	54.4	-	73.7	-	84.8	-	92.7	-	95.9	-
	
	0.1^-1^	35.8	-	58.7	-	78.7	-	90.7	-	95.2	-
	
	0.2^-1^	16.4	-	33.2	-	56.1	-	80.9	-	90.2	-
	
	0.3^-1^	6.1	-	20.4	-	36.8	-	61.5	-	80.3	-
	
	0.4^-1^	3.0	-	8.6	-	24.2	-	47.1	-	66.6	-
	
	0.5^-1^	1.8	-	3.7	-	10.7	-	31.9	-	47.0	-

Accuracy was computed as the average of precision and recall. The first column in table shows the type of selection (gain, loss, or acceleration), and the value of the scaling parameter ρ. Smaller values of ρ correspond to stronger selection pressure, which is easier to detect. Results in bold show the larger accuracy value for each comparison between DivE and DLESS.

In almost all cases, DivE's accuracy is significantly greater than that obtained by DLESS. In the few cases where DLESS obtains a better accuracy, this accuracy is less than 0.5% greater than that of DivE. As observed before [26] the power to predicting elements that are lost is greater than the power to predicted elements that are gained. Actually, in the case of lineage-specific "gains" (the upper third of Table 1), the average accuracy only surpasses 50% for elements longer than 500 bp.

**Figure 3:**
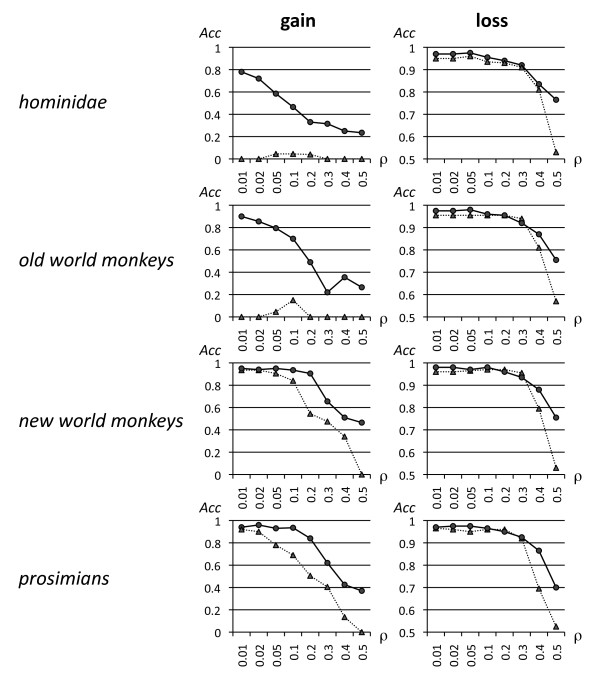
**Accuracy of gain and loss predictions on the major primate clades for negatively selected regions**. All regions are 200 bp in length. DivE is shown with a continuous line, and DLESS with a dotted line. On the *x *axis is the value of the scaling parameter ρ determining the selection strength, while the accuracy (the average of precision and recall) is shown on the y axis.

Figure 3 shows that accuracy can be greater when considering specific clades with more species, and longer branch lengths. In fact the power of detection gets significantly higher if the clade has at least two species compared to the power of detection in a single species (see Additional file 1, Tables S1-S8). In the case of "losses," the power is generally quite good especially for elements of 100 bp and greater.

Table 1 also shows the accuracy obtained by DivE on predicting elements that are accelerated, a function lacking in DLESS. There is no program that we are aware of that is designed to detect both the accelerated element as well as the specific phylogenetic lineage in which the acceleration takes place. It is interesting to note that the power of detecting accelerated elements is significantly greater than the power to predict "gained" elements, although not as good as the power to detect "lost" elements (see also Additional file 1, Tables S17-S24).

We should also note that by design DivE cannot distinguish between accelerations in the simian clade and accelerations in the prosimian clades. This is due to the symmetry of the phylogenetic tree in which enlargements in the branches from the phylogeny's root to the simians and to the prosimians are indistinguishable. If acceleration in any of these clades was detected, we assumed a 50% chance that the acceleration was in either clade. In real applications, an outgroup can be used to distinguish between these two cases.

Both DivE and DLESS can predict conservation not only on subtrees of the phylogenetic tree, but on all branches of the phylogeny as well. For this task, DivE's accuracy is again significantly better than that of DLESS, as shown in Table 2. In this case, the power of detecting elements under negative selection is quite good even for elements as small as 50 bp, when *ρ *≤ 0.2. The accuracy of detection increases significantly for elements bigger than 100 bp, and is over 90% for *ρ *= 0.3, a value that has been shown to be specific to conserved regions in vertebrates [10].

**Table 2:** Accuracy of DivE (dE) and DLESS (dl) on detecting negative selection in all primates using simulated data.

selection strength	50 bp		100 bp		200 bp		500 bp		1000 bp	
	
	dE	dl	dE	dl	dE	dl	dE	dl	dE	dl
0.01	**93.6**	92.3	**97.3**	88.2	**98.2**	94.2	**99.3**	94.3	**99.6**	96.7

0.02	**93.4**	86.2	**96.7**	92.3	**98.3**	91.4	**99.3**	93.2	**99.7**	94.1

0.05	**94.2**	86.0	**95.6**	87.8	**98.2**	95.2	**99.4**	95.7	**99.6**	98.6

0.1	**94.9**	85.5	**95.9**	90.9	**98.1**	92.9	**99.2**	95.1	**99.6**	96.1

0.2	**79.1**	64.3	**95.3**	94.5	**97.8**	92.8	**99.0**	94.7	**99.5**	95.0

0.3	**64.4**	47.4	**93.3**	87.2	**95.7**	95.2	**98.5**	91.7	**99.2**	96.3

0.4	**49.7**	23.4	**75.2**	65.0	**93.6**	89.8	**97.7**	91.6	**98.8**	91.2

0.5	**37.5**	8.4	**62.7**	46.2	**85.5**	70.0	**94.9**	83.6	**97.2**	87.1

Results in bold show the greater accuracy.

### Results on fourteen primate species

Next we applied DivE to the multiple alignments of the 44 ENCODE regions [30] of primates (see Methods). These regions cover ~1% of the human genome sequence and represent the largest mammalian comparative data set yet published [31]. For our analysis, we focused exclusively on the primate sequences because DivE is designed to detect selection in closely related groups of species.

When run on all primate ENCODE regions, DivE predicted 21,633 elements with non-neutral substitution rates. These elements had lengths ranging from 50 bp to 40,000 bp, with an average length of 700 bp. Of all predicted elements, 12,385 (covering ~28% of the 32.1 million columns of the ENCODE regions' alignment) showed negative selection on either all or a subset of the branches of the phylogenetic tree. This coverage is even higher in protein coding regions and UTRs, where it reaches 74%, and 49% respectively. About 65% of the predicted sites undergoing negative selection were either fully conserved or lineage-specific gains, while the rest of them were lineage-specific losses.

DivE predicted 9248 lineage-specific acceleration events, covering 20% of all columns in the ENCODE alignment of the primates. Given its reported power to detect acceleration for a given clade [24], we ran phyloP to validate our predictions. PhyloP reported p-values of 0.05 or less for 84% of our predicted accelerated elements. By comparison, phyloP reported a p-value below 0.05 for 98% of the elements that are predicted to be under negative selection, either within a subtree or on all branches of the phylogeny. This suggests that DivE may have somewhat less power to detect acceleration as opposed to conservation. Additional file 1, Table S25 shows the exact percentages of the ENCODE primate sequences predicted to be either conserved or accelerated by DivE.

We were particularly interested in regions for which we can find evidence of positive selection. As a stricter criterion for positive selection, we looked at the 9248 accelerated regions and asked whether, in each case, the subtree involved was internally conserved; i.e., after the acceleration event, the sequences were conserved more than expected (see Figure 2). This filter eliminates cases of apparent acceleration that might be artifacts of low quality sequence or mis-alignment. DivE predicted 4942 regions (about 53% of all predictions) to be lineage-specifically accelerated (p-value < 0.05 as determined by phyloP) where the regions also appear conserved when looking only at the subtree for that particular lineage. (These regions include both one-node subtrees and clades of 2 or more species). A list of all elements that are potentially undergoing positive selection, as well as all elements predicted by DivE can be downloaded from ftp://cbcb.umd.edu/pub/data/DivE.

Positive selection (PS) is one of the most important evolutionary forces that shape our development, and many efforts have been made to detect its presence in protein-coding genes. The most widely used methods is to determine if the ratio of nonsynonymous (dN) to synonymous (dS) substitutions is larger than 1 [32]. This only works in protein-coding regions, while DivE considers all regions, but we wanted to compare its predictions in protein-coding regions with those made by a standard method.

We used the dN/dS ratio estimated with the PAML package [33] to test for positive selection (PS) in primate genes from the ENCODE regions that overlap DivE's predicted accelerated elements. There are 443 human genes with annotations in the ENCODE regions. Out of these, 182 genes overlap with accelerated elements predicted by DivE. Because the other primates were not annotated, we used Jigsaw [34] for each human gene (see Methods) to determine its equivalent annotation in the other primates. Similarly to previous work [35], to minimize the false positive rate when predicting positive selection, we accepted as valid only transcripts that aligned to at least 80% of the human CDS, without frame shifts. At each gene locus we only retained the transcripts that had the longest overlap with the accelerated predicted element. By selecting for each gene the transcript that was conserved in most species, and using the longest coding length in deciding ties, we obtained 80 genes that mapped to at least two other species besides human and overlapped the predicted accelerated elements. This procedure resulted in an average of 10 orthologous transcripts for each gene.

Using PAML to test for PS on the branches predicted as accelerated by DivE, we determined 25 genes with p-value < 0.05. After multiple testing correction, 20 genes were identified as positively selected genes (PSGs). These genes, shown in Table 3, represent only 25% of our initial 80 genes; i.e., only one in four genes that were found to intersect accelerated elements were predicted as PSGs. The remaining genes might be false positives found by DivE, or alternatively, they might have been missed because fewer sites among their CDS's were found to be accelerated than for the 25% that were consistent with the PAML analysis of PS (246 bp vs 383 bp in average), and PAML itself may have less power to determine PS on whole coding regions where only small fractions of them show acceleration. Also, as one recent study found [36], positive selection may be limited to specific regions of genes that are otherwise conserved. As mentioned above, the subtrees tested for the presence of PS were the ones predicted by DivE to be under acceleration. In cases where the acceleration was predicted in either the simians or the prosimians clade, we tested both clades for the presence of PS, and we reported the one with the highest p-value. It should be noted though that in most cases PAML finds very close p-values for both clades.

**Table 3:** List of positively selected genes that intersect DivE's predicted accelerated regions.

Gene symbol	Gene full name	Encode region	Subtree under selection	No. of orthologous sequences	P-value
*SERPINB7*	serpin peptidase inhibitor, clade B (ovalbumin), member 7	ENr122	new world monkeys	13	<.0001

*SLC22A4*	solute carrier family 22 (organic cation/ergothioneine transporter), member 4	ENm002	orangutan	14	<.0001

*UGT1A8*	UDP glucuronosyltransferase 1 family, polypeptide A8	ENr131	baboon	7	<.0001

*LILRA4*	leukocyte immunoglobulin-like receptor, subfamily A (with TM domain), member 4	ENm007	human-orangutan	6	<.0001

*APOA4*	apolipoprotein A-IV	ENm003	macaque	13	<.0001

*HBQ1*	hemoglobin, theta 1	ENm008	chimp	14	<.0001

*F7*	coagulation factor VII (serum prothrombin conversion accelerator)	ENr132	macaque	11	<.0001

*C21orf13* *(LCA5L)*	Leber congenital amaurosis 5-like	ENr133	mouse lemur	13	0.0004

*CGN*	cingulin	ENr231	dusky titi	13	0.0004

*LILRB4*	leukocyte immunoglobulin-like receptor, subfamily B (with TM and ITIM domains), member 4	ENm007	vervet-baboon	6	0.0007

*AC006985.5* *(HEATR7B1)*	HEAT repeat containing 7B1	ENr131	prosimians	9	0.0007

*ARHGDIG*	Rho GDP dissociation inhibitor (GDI) gamma	ENm008	simians	10	0.0017

*F8*	coagulation factor VIII, procoagulant component	ENm006	owl monkey	10	0.0026

*SYT8*	synaptotagmin VIII	ENm011	new world monkeys	8	0.0030

*C22orf30 (PRR14L)*	proline rich 14-like	ENm004	simians	12	0.0039

*HBZ*	hemoglobin, zeta	ENm008	macaque	4	0.0043

*LILRB5*	leukocyte immunoglobulin-like receptor, subfamily B (with TM and ITIM domains), member 5	ENm007	macaque	3	0.0068

*DEPDC5*	DEP domain containing 5	ENm004	human-colobus monkey	12	0.007

*RPS9*	ribosomal protein S9	ENm007	human-colobus monkey	13	0.0227

*DDX43*	DEAD (Asp-Glu-Ala-Asp) box polypeptide 43	ENr223	new world monkeys	12	0.0246

Positive selection was determined with the *codeml *program from the PAML package. The first column gives the ENCODE gene symbol followed in parentheses by the corresponding Genbank gene symbol (if different). The fourth column in the table shows the name of the subtree predicted to be accelerated by DivE. If there are multiple species in the selected tree, the clade is specified by the two species whose most common ancestor form the clade's root, or by the clade's name as specified in Figure 1.

Finally we looked at gene ontology (GO) [37] categories associated with the identified PSGs. The two most common GO categories were GO:0016021 ('integral to membrane') and GO:0005886 ('plasma membrane'). The first category has been previously found to be over-represented among mammalian genes predicted to be under positive selection [35]. In fact 8 out of our 20 PSGs are associated with over-represented GO categories among mammalian PSGs (see Additional file 1, Table S26).

## Discussion

In this study we introduced DivE, a new method to detect lineage-specific selection in a group of closely related species. In contrast to most previous methods, our approach does not restrict its search to selection events in a particular lineage, but rather tries to discover the particular clade undergoing selection as well as the type of selection. We should note that DivE does not specifically detect positive selection, but rather identifies regions undergoing accelerated change. An accelerated rate of evolution can be used as an indication that positive selection has occurred, but it could also be due to other factors, including relaxation of selective constraints.

Results on simulated data suggest that this method performs as well or better than earlier methods, especially for longer sequences, and its performance in discovering negative selection is comparable to similar methods both on real and simulated data. DivE also has an advantage in that it does not make any assumptions about the expected length of the regions under selection, or about the strength of the evolutionary constraint. On the other hand, our method admittedly makes the simplifying assumption that the neutral rate of point mutation is uniform over the genome (although local neutral substitution rates could be estimated *a priori *and provided as input to DivE). Neither does it model insertion and deletion events, but rather it assumes that data is missing when it encounters a deletion. Although this assumption is not always realistic, it is probably true for most deletions appearing in the ENCODE regions of the non-human primates. Nevertheless these assumptions are likely to influence the results. Also, compared to other methods, DivE is not a rigorously statistical method, but rather a heuristic approach designed to capture regions with scores higher than the false positive scores observed in neutrally simulated sequences whose length and composition is similar to the real data. Such regions are then predicted as selected. One strength of the method is its fast running time for discovering lineage-specific selected regions, which then can be further evaluated with statistical methods (e.g., phyloP).

We focused on primate genomes in our study because of their obvious relevance to human, and also because sequence data is now available for 14 different primates for a select set of regions covering approximately 1% of each genome. The very close evolutionary relationship of these species required the development of a new method that could detect signs of possible selection in very recently diverged species. We were particularly interested in positive selection, because rapidly evolving regions might be key to explaining the unique phenotypic traits that distinguish humans from other primates and the primates from one another. The DivE program predicted accelerated regions overlapping 182 genes in the ENCODE regions. Not all evolutionary acceleration events are caused by adaptive evolution [38], and therefore DivE alone should not be used to infer positive selection. Using the ratio of nonsynonymous to synonymous substitution, we found evidence of positive selection in different lineages of the primates in 20 genes, representing about 5% of all known ENCODE genes that have valid transcripts in at least two other primates. These 20 genes represent only 25% of all the genes predicted to intersect accelerated regions. These results agree with an analysis of positive selection and acceleration in the evolution of the human and chimp genomes [19], where the authors found that many of the genes with accelerated rates did not show significant signals of positive selection. Anyway, as a recent study points out [39], these results should be treated with caution because estimates of positive selection can be greatly influenced by errors in sequencing, annotation and alignment. Although the ENCODE regions for these 14 primates are between 17 and 30 Million bp (see Additional file 1, Table S25) they are still not 100% complete [40], and they represent only a small fraction of the whole genomes. They also contain less than 2% of the approximately 22,000 known human genes [41]. Therefore the results presented here might not extrapolate well to an alignment of complete genomes.

## Conclusions

The evolutionary events relating a group of species can often be discerned by searching for patterns in the aligned DNA sequences from those species. These patterns can reveal the critical changes that make each species unique and different from its relatives. Regions of strong sequence conservation are a sign of negative (or purifying) selection, in which detrimental changes are eliminated. Positive selection, in which beneficial changes are retained in a species, can appear as a region in a multi-species alignment with many more changes than expected. Different selective pressures can act on different lineages of a phylogeny. Although computational methods have been developed to identify regions under selection across species or in a particular lineage, most of these are limited to protein coding sequences only, or to identifying negative selection, or both. Little attention has been given to identifying selection pressure on both coding and non-coding sequences for any branch of a phylogeny.

Here we developed a heuristic novel approach that can discover either positive or negative selection on any lineage in a phylogeny, without making prior assumptions about the strength of the selective pressure. We have implemented our method in an open-source software package, called DivE, and applied it to identify lineage-specific selection in a group of fourteen primates.

## Methods

### Dataset

We downloaded the 44 ENCODE [30] sequences and their multiple sequence alignments for 14 primates (human, chimp, orangutan, gibbon, colobus monkey, vervet, baboon, macaque, dusky titi, owl monkey, marmoset, squirrel monkey, mouse lemur, galago), along with their associated neutral evolution phylogenetic model (as defined in section 3.2) from the NHGRI site (ftp://kronos.nhgri.nih.gov/pub/outgoing/elliott/encode/freeze/JAN-2009/). The tree topology, shown in Figure 1, follows the NCBI taxonomy (http://www.ncbi.nlm.nih.gov/guide/taxonomy/), while the support values for the nodes are from [42,43]. The alignments were generated with the TBA program [44] using the method described elsewhere [31]. The neutral model was estimated from all fourfold degenerate sites in the ENCODE regions, using the phyloFit program [29] from the PHAST package [45], and assuming the general time-reversible (REV) substitution model [46].

### Finding regions under selection

We constructed the program called DivE (Divergence by Evolution) to detect lineage-specific selection by identifying conserved or accelerated elements specific only to a subgroup of species. Given a multiple alignment of orthologous sequences, and two phylogenetic models, one describing regions evolving under neutral evolution and one describing regions under selection in a particular lineage, DivE employs a greedy heuristic to determine local regions in the alignment where the following log ratio score:(1)

is positive and maximal.

**Figure 4:**
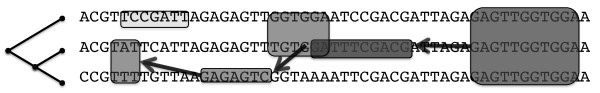
**Schematic figure of DivE's heuristic approach**. For each type of selection, and each node in the phylogenetic tree, first we identify local regions (denoted by shadowed rectangles in the figure) that score positively according to a log ratio selection score - the more intense the shadow color, the higher the score of that region is. Then DivE predicts as "under selection" the maximal scoring non-overlapping regions. The arrows in the figure connect predicted regions in the alignment.

We evaluate the score in equation (1) for every lineage in the phylogeny (which corresponds to a single node in the phylogenetic model), between any two columns in the alignment, and for any type of selection. If the score for a given lineage in the alignment region between the two columns is positive and above some fixed threshold, then we consider this an indication that the lineage has potentially undergone selection in that particular region. The region is discarded in the case where it contains another region whose score represents a significant deviation from selection (according to a previously identified threshold). This prevents the algorithm from clustering together nearby selected regions. If two candidate regions overlap, we predict as under selection only the maximum scoring region (see also Figure 4). Below we present our method for computing the score in equation (1).

As in [47], we specify the neutral evolution phylogeny of the species through a phylogenetic tree model with four parameters: *ψ^n^*= (*Q, τ, β, π*), where *Q *is a substitution rate matrix, *τ *is the topology of the phylogenetic tree, *β *is a vector of branch lengths, and *π *is a vector of equilibrium base frequencies. *τ *consists of a set of nodes, *V(τ*), and a set of edges, *E(τ*), connecting the nodes. Given a positive number *ρ*, and the subtrees *τ'*, *τ'' *of *τ*, we will denote by *ρβ *the lengths of branches in multiplied by *ρ*, by *β|E(τ') *the lengths of branches in *τ' *only, and by *β| E (τ') | ∪ β | E (τ'') *the lengths of branches in *τ' *and *τ''*. If *u ∈V (τ)*, we denote by *τ*_*u *_a subtree of *τ *rooted at *u*, and by (*u_p_,u*) the parent edge of *u*, where *u_p _*is the parent node of *u*. With these notations, we can now specify the phylogenetic models governing the gain (*g*), loss (*l*) and acceleration (*a*) evolutionary events relative to the neutral model ψ as , , and  respectively, where *ρ *∈ (0,1) is the scaling parameter that identifies the selection strength of the model.

We can now give a more detailed form for the score computed by DivE in equation (1). For each two columns *i *and *j *in an alignment of sequences, with *i *<*j*, selection type *s *∈ {*g*,*l*,*a*}, and node *u *∈ *V *(*τ*), DivE computes the following score:(2)

where *p(k;ψ) *is the probability of the column *k *in the alignment under the phylogenetic model *ψ*. We can compute the probability *p(k;ψ) *using the Felsenstein algorithm [48]. As shown in equation (2) the scaling parameter *ρ *is not fixed in the input of the program in order to avoid assumptions about the selection strength in the observed local alignment. In the actual implementation of DivE, for practical purposes, *ρ *is not left to vary freely in the (0,1) range, but there is a fixed number of possible values for *ρ *that DivE can choose from. This number and the possible values for *ρ *can be changed in the program's input. The results presented here were generated with *ρ *allowed to vary in the (0.05,0.1,0.2,0.3,0.4,0.5) set.

If *n *represents the number of sequences in the alignment, and *L *is the length of the alignment, than computing the score in equation (2) between any two columns in the alignment would lead to an *O(nL^2^) *running time complexity for DivE's heuristics. The actual implementation of DivE has a running time of *O(nLa) *where *a *represents the average length of a selected element, since we don't need to evaluate a score between any *i *and *j (i<j*) columns, if the region between the two columns contains a subregion that is neutral or shows selection of a different type (according to a predefined threshold). This linear running time of our heuristics makes DivE particularly well-suited to detecting lineage-specific selection in large genomes.

### Simulations

We used the program *evolver *from the PAML package [33], version 3.15, to simulate aligned nucleotide sequences evolving either neutrally or under selection. Aligned neutral sequences were generated to follow the neutral model of evolution described in the Dataset section, and they were of the same length (about 32.1 million bp) and with the same deletion patterns as the aligned ENCODE regions of the 14 primates. We used them to determine thresholds for DivE to call predicted elements with a false positive rate of less than 0.1%. To simulate sites under selection in a given subtree of the phylogeny the scaling parameter *ρ*<1 was used to adjust the branch lengths of the phylogeny either through multiplication of all branches' lengths in the selected subtree (in the case of gain and loss), or through division of the length of the parent edge from the root of the subtree (in the case of acceleration). The sites under section were chosen to span regions of 50, 100, 200, 500, or 1000 aligned columns that were randomly inserted inside neutrally evolving region that was twice as long, flanked on either side by another 1000 neutral alignment columns. For any given subtree, length, and scaling parameter we generated 100 such regions containing sites under selection, resulting in a total number of 243,000 simulated regions, that we used to compute the accuracy of prediction. The accuracy was computed as the average of the recall (the number of sites under selection correctly identified, also known as sensitivity) and precision (the number of predicted sites that were truly under selection.

### Gene annotations for the ENCODE regions

We downloaded the GENCODE reference set [49] of human gene annotations for the ENCODE regions from the UCSC genome browser [50], and we aligned the known human mRNA sequences from each ENCODE region to the corresponding genomic region of every primate species. The nucleotide sequences of these mRNAs were mapped using GMAP [51] and sim4cc [52]. Where available, the coding regions of these human transcripts were translated into proteins and aligned to the corresponding ENCODE regions in the other primates using PMAP (a protein mapping variant of GMAP) and exonerate [53]. The resulting spliced alignments were integrated using JIGSAW [34], which reported the combined transcript structures that had valid coding regions.

### Identification of genes under positive selection

Positively selected genes were identified using the *codeml *program from PAML. As input, we used the tree in Figure 1, and the TBA alignments of the genes extracted from the ENCODE regions. The same procedure using the improved branch-site test 2 under "model A" described in [54] was used to determine significance p-values for positively selected genes in a specific lineage. The codon frequencies were estimated from the average nucleotide frequencies at the three-codon position (F3 × 4 model). Test 2 has been shown to be the more robust in detecting positive selection among the other branch-site models implemented in *codeml*. The null hypothesis of this test assumes that some of the branches are negatively selected and some are neutral, while for the alternative hypothesis some sites are allowed to undergo positive selection (dN/dS >1) along some predefined foreground branch, which in our case was selected to be the parent branch of the node at the root of the subtree predicted to be under acceleration by DivE in the coding portion of the gene. The LRT's values resulted from test 2 were compared against the distribution *X*^1 ^to determine significance p-values. Correction for multiple testing was performed using the Benjamini and Hochberg procedure [55] using a false discovery rate (FDR) of 10% as the tests for PS are already very conservative [54].

## Authors' contributions

MP and SLS designed the DivE method. MP and GP implemented the algorithms. GP generated the gene annotations in the ENCODE regions for all non-human primates, and MP analyzed the data. MP and SLS wrote the paper. All authors read and approved the final manuscript.

## Supplementary Material

Additional file 1 **Supplementary Tables S1-S26**. This document includes the accuracy obtained by DivE and DLESS for the prediction of all simulated elements, the percentage of the ENCODE primate sequences predicted to be either conserved or accelerated by DivE, and the GO categories associated with genes predicted to be under positive selection. Click here for file
