# Supplementary material for: Detection of lineage-specific evolutionary changes among primate species
Source: BMC Bioinformatics. 2011 Jul 4;12:274. doi: 10.1186/1471-2105-12-274 (PMC3143108; doi:10.1186/1471-2105-12-274)
Supplement: Additional file 1 — Supplementary Tables S1-S26. This document includes the accuracy obtained by DivE and DLESS for the prediction of all simulated elements, the percentage of the ENCODE primate sequences predicted to be either conserved or accelerated by DivE, and the GO categories associated with genes predicted to be under positive selection. [file 1471-2105-12-274-S1.DOC]

| 0.01 gain | | **50 bp** | | **100 bp** | | **200 bp** | | **500 bp** | | **1000 bp** | |
| --- | --- | --- | --- | --- | --- | --- | --- | --- | --- | --- | --- |
| **tree** | **prog** | Pr | Re | Pr | Re | Pr | Re | Pr | Re | Pr | Re |
| human | DivE | 0 | 0 | 0 | 0 | 0 | 0 | 0 | 0 | 0 | 0 |
|  | dless | 0 | 0 | 0 | 0 | 0 | 0 | 0 | 0 | 0.25 | 0.02 |
| chimp | DivE | 0 | 0 | 0 | 0 | 0 | 0 | 0 | 0 | 0 | 0 |
|  | dless | 0 | 0 | 0 | 0 | 0 | 0 | 0 | 0 | 0.29 | 0.02 |
| human -chimp | DivE | 0 | 0 | 0 | 0 | 0 | 0 | 0.59 | 0.10 | 0.89 | 1.00 |
|  | dless | 0.02 | 0.01 | 0.05 | 0.01 | 0 | 0 | 0 | 0 | 0.54 | 0.20 |
| orangutan | DivE | 0 | 0 | 0 | 0 | 0 | 0 | 0.35 | 0.01 | 0.83 | 0.98 |
|  | dless | 0 | 0 | 0 | 0 | 0 | 0 | 0.08 | 0.01 | 0.45 | 0.04 |
| human -orangutan | DivE | 0 | 0 | 0 | 0 | 0.41 | 0.02 | 0.85 | 1.00 | 0.92 | 1.00 |
|  | dless | 0.01 | 0.00 | 0 | 0 | 0 | 0 | 0.50 | 0.07 | 0.89 | 1.00 |
| gibbon | DivE | 0 | 0 | 0 | 0 | 0 | 0 | 0.51 | 0.08 | 0.88 | 1.00 |
|  | dless | 0 | 0 | 0 | 0 | 0 | 0 | 0.17 | 0.01 | 0.55 | 0.03 |
| human - gibbon | DivE | 0 | 0 | 0.22 | 0.01 | 0.73 | 0.83 | 0.91 | 1.00 | 0.95 | 1.00 |
|  | dless | 0 | 0 | 0 | 0 | 0 | 0 | 0.88 | 1.00 | 0.94 | 0.99 |
| colobus monkey | DivE | 0 | 0 | 0 | 0 | 0 | 0 | 0.41 | 0.03 | 0.79 | 0.96 |
|  | dless | 0 | 0 | 0 | 0 | 0 | 0 | 0.17 | 0.01 | 0.31 | 0.05 |
| vervet | DivE | 0 | 0 | 0 | 0 | 0 | 0 | 0 | 0 | 0.61 | 0.21 |
|  | dless | 0 | 0 | 0 | 0 | 0 | 0 | 0 | 0 | 0.46 | 0.01 |
| baboon | DivE | 0 | 0 | 0 | 0 | 0 | 0 | 0 | 0 | 0.52 | 0.08 |
|  | dless | 0 | 0 | 0 | 0 | 0 | 0 | 0 | 0 | 0.25 | 0.01 |
| macaque | DivE | 0 | 0 | 0 | 0 | 0 | 0 | 0.17 | 0.01 | 0.38 | 0.03 |
|  | dless | 0 | 0 | 0 | 0 | 0.08 | 0.01 | 0 | 0 | 0.25 | 0.02 |
| baboon - macaque | DivE | 0 | 0 | 0 | 0 | 0.09 | 0.01 | 0.61 | 0.22 | 0.88 | 1 |
|  | dless | 0 | 0 | 0 | 0 | 0.08 | 0.01 | 0 | 0 | 0.69 | 0.5 |
| vervet - baboon | DivE | 0 | 0 | 0 | 0 | 0.31 | 0.02 | 0.85 | 1 | 0.91 | 1 |
|  | dless | 0 | 0 | 0 | 0 | 0 | 0 | 0.61 | 0.14 | 0.89 | 1 |
| colobus monkey - vervet | DivE | 0 | 0 | 0.23 | 0.03 | 0.8 | 1 | 0.91 | 1 | 0.96 | 1 |
|  | dless | 0 | 0 | 0.05 | 0 | 0 | 0 | 0.91 | 1 | 0.95 | 1 |
| human - colobus monkey | DivE | 0.29 | 0.04 | 0.77 | 0.98 | 0.92 | 1 | 0.95 | 1 | 0.98 | 1 |
|  | dless | 0 | 0 | 0.56 | 0.01 | 0.89 | 1 | 0.95 | 1 | 0.97 | 1 |
| dusky titi | DivE | 0 | 0 | 0 | 0 | 0 | 0 | 0.77 | 0.8 | 0.89 | 1 |
|  | dless | 0 | 0 | 0 | 0 | 0 | 0 | 0 | 0 | 0.84 | 0.95 |
| owl monkey | DivE | 0 | 0 | 0 | 0 | 0 | 0 | 0.61 | 0.07 | 0.89 | 1 |
|  | dless | 0 | 0 | 0 | 0 | 0 | 0 | 0 | 0 | 0.54 | 0.23 |
| marmoset | DivE | 0 | 0 | 0 | 0 | 0 | 0 | 0.7 | 0.58 | 0.88 | 1 |
|  | dless | 0 | 0 | 0 | 0 | 0 | 0 | 0.25 | 0.01 | 0.84 | 0.96 |
| squirrel monkey | DivE | 0 | 0 | 0 | 0 | 0 | 0 | 0.69 | 0.77 | 0.86 | 1 |
|  | dless | 0 | 0 | 0 | 0 | 0 | 0 | 0 | 0 | 0.8 | 0.96 |
| marmoset - squirrel monkey | DivE | 0 | 0 | 0 | 0 | 0.55 | 0.25 | 0.87 | 1 | 0.91 | 1 |
|  | dless | 0 | 0 | 0 | 0 | 0 | 0 | 0.88 | 1 | 0.9 | 1 |
| owl monkey - marmoset | DivE | 0 | 0 | 0.44 | 0.01 | 0.81 | 0.99 | 0.94 | 1 | 0.96 | 1 |
|  | dless | 0 | 0 | 0 | 0 | 0 | 0 | 0.9 | 1 | 0.95 | 1 |
| dusky titi - owl monkey | DivE | 0 | 0 | 0.74 | 0.74 | 0.88 | 1 | 0.94 | 1 | 0.98 | 1 |
|  | dless | 0 | 0 | 0 | 0 | 0.86 | 0.98 | 0.94 | 0.99 | 0.97 | 1 |
| human – dusky titi | DivE | 0.81 | 0.96 | 0.9 | 1 | 0.96 | 1 | 0.98 | 1 | 0.99 | 1 |
|  | dless | 0.64 | 0.14 | 0.87 | 0.98 | 0.94 | 1 | 0.97 | 1 | 0.99 | 1 |
| mouse lemur | DivE | 0 | 0 | 0 | 0 | 0.52 | 0.12 | 0.83 | 1 | 0.94 | 1 |
|  | dless | 0 | 0 | 0 | 0 | 0 | 0 | 0.8 | 0.7 | 0.92 | 1 |
| galago | DivE | 0 | 0 | 0 | 0 | 0.78 | 0.92 | 0.93 | 1 | 0.96 | 1 |
|  | dless | 0 | 0 | 0 | 0 | 0.38 | 0.01 | 0.91 | 1 | 0.94 | 1 |
| mouse lemur - galago | DivE | 0.18 | 0.02 | 0.77 | 0.98 | 0.9 | 1 | 0.95 | 1 | 0.98 | 1 |
|  | dless | 0 | 0 | 0.18 | 0.03 | 0.88 | 0.99 | 0.94 | 1 | 0.97 | 1 |

**Table S1.** Accuracy obtained by DivE and DLESS for the prediction of gained elements (conserved within a subtree, but evolving at the neutral rate outside the subtree) of different lengths in each clade^1^ of the phylogeny, when the selction strength is given by ρ=0.01.

| 0.02 gain | | **50 bp** | | **100 bp** | | **200 bp** | | **500 bp** | | **1000 bp** | |
| --- | --- | --- | --- | --- | --- | --- | --- | --- | --- | --- | --- |
| **tree** | **prog** | Pr | Re | Pr | Re | Pr | Re | Pr | Re | Pr | Re |
| human | DivE | 0 | 0 | 0 | 0 | 0 | 0 | 0 | 0 | 0 | 0 |
|  | dless | 0 | 0 | 0 | 0 | 0 | 0 | 0 | 0 | 0.25 | 0.01 |
| chimp | DivE | 0 | 0 | 0 | 0 | 0 | 0 | 0 | 0 | 0.28 | 0.01 |
|  | dless | 0 | 0 | 0 | 0 | 0.08 | 0.01 | 0 | 0 | 0.25 | 0.03 |
| human -chimp | DivE | 0 | 0 | 0 | 0 | 0 | 0 | 0.53 | 0.14 | 0.87 | 0.99 |
|  | dless | 0 | 0 | 0 | 0 | 0 | 0 | 0.23 | 0.01 | 0.47 | 0.10 |
| orangutan | DivE | 0 | 0 | 0 | 0 | 0 | 0 | 0.12 | 0.01 | 0.82 | 0.99 |
|  | dless | 0 | 0 | 0 | 0 | 0 | 0 | 0 | 0 | 0 | 0 |
| human -orangutan | DivE | 0 | 0 | 0 | 0 | 0.44 | 0.01 | 0.87 | 0.99 | 0.92 | 1.00 |
|  | dless | 0 | 0 | 0 | 0 | 0.08 | 0.00 | 0.67 | 0.06 | 0.89 | 1.00 |
| gibbon | DivE | 0 | 0 | 0 | 0 | 0 | 0 | 0.44 | 0.06 | 0.84 | 0.99 |
|  | dless | 0 | 0 | 0 | 0 | 0 | 0 | 0 | 0 | 0.54 | 0.11 |
| human - gibbon | DivE | 0.19 | 0.02 | 0 | 0 | 0.74 | 0.70 | 0.92 | 1.00 | 0.96 | 1.00 |
|  | dless | 0.02 | 0 | 0 | 0 | 0 | 0 | 0.89 | 1.00 | 0.95 | 1.00 |
| colobus monkey | DivE | 0 | 0 | 0 | 0 | 0 | 0 | 0.5 | 0.02 | 0.86 | 0.94 |
|  | dless | 0 | 0 | 0 | 0 | 0 | 0 | 0 | 0 | 0.38 | 0.01 |
| vervet | DivE | 0 | 0 | 0 | 0 | 0.05 | 0.01 | 0 | 0 | 0.65 | 0.17 |
|  | dless | 0 | 0 | 0 | 0 | 0 | 0 | 0 | 0 | 0.38 | 0.01 |
| baboon | DivE | 0 | 0 | 0 | 0 | 0 | 0 | 0.15 | 0.01 | 0.53 | 0.03 |
|  | dless | 0 | 0 | 0 | 0 | 0 | 0 | 0 | 0 | 0 | 0 |
| macaque | DivE | 0 | 0 | 0 | 0 | 0.06 | 0.01 | 0 | 0 | 0.58 | 0.02 |
|  | dless | 0 | 0 | 0 | 0 | 0 | 0 | 0 | 0 | 0.25 | 0.01 |
| baboon - macaque | DivE | 0 | 0 | 0 | 0 | 0.13 | 0.01 | 0.58 | 0.2 | 0.87 | 1 |
|  | dless | 0 | 0 | 0 | 0 | 0 | 0 | 0.14 | 0.01 | 0.71 | 0.48 |
| vervet - baboon | DivE | 0 | 0 | 0 | 0 | 0.36 | 0.02 | 0.86 | 0.98 | 0.92 | 1 |
|  | dless | 0 | 0 | 0 | 0 | 0 | 0 | 0.47 | 0.1 | 0.9 | 1 |
| colobus monkey - vervet | DivE | 0 | 0 | 0.22 | 0.02 | 0.79 | 0.92 | 0.92 | 1 | 0.95 | 1 |
|  | dless | 0 | 0 | 0 | 0 | 0 | 0 | 0.89 | 1 | 0.94 | 1 |
| human - colobus monkey | DivE | 0.56 | 0.01 | 0.8 | 0.97 | 0.9 | 1 | 0.95 | 1 | 0.98 | 1 |
|  | dless | 0.02 | 0 | 0.68 | 0.03 | 0.87 | 0.99 | 0.94 | 1 | 0.97 | 0.99 |
| dusky titi | DivE | 0 | 0 | 0 | 0 | 0.11 | 0.01 | 0.77 | 0.67 | 0.9 | 1 |
|  | dless | 0 | 0 | 0 | 0 | 0 | 0 | 0 | 0 | 0.86 | 0.88 |
| owl monkey | DivE | 0 | 0 | 0 | 0 | 0.18 | 0.01 | 0.48 | 0.13 | 0.85 | 0.99 |
|  | dless | 0 | 0 | 0 | 0 | 0 | 0 | 0 | 0 | 0.63 | 0.21 |
| marmoset | DivE | 0 | 0 | 0 | 0 | 0 | 0 | 0.77 | 0.5 | 0.87 | 1 |
|  | dless | 0 | 0 | 0 | 0 | 0 | 0 | 0 | 0 | 0.82 | 0.84 |
| squirrel monkey | DivE | 0 | 0 | 0 | 0 | 0.26 | 0.01 | 0.76 | 0.76 | 0.87 | 1 |
|  | dless | 0 | 0 | 0 | 0 | 0 | 0 | 0.26 | 0.01 | 0.78 | 0.89 |
| marmoset - squirrel monkey | DivE | 0 | 0 | 0 | 0 | 0.55 | 0.16 | 0.91 | 1 | 0.94 | 1 |
|  | dless | 0 | 0 | 0 | 0 | 0 | 0 | 0.85 | 0.96 | 0.92 | 1 |
| owl monkey - marmoset | DivE | 0 | 0 | 0.2 | 0.02 | 0.81 | 0.95 | 0.91 | 1 | 0.96 | 1 |
|  | dless | 0 | 0 | 0 | 0 | 0.59 | 0.04 | 0.9 | 1 | 0.94 | 0.99 |
| dusky titi - owl monkey | DivE | 0 | 0 | 0.77 | 0.7 | 0.92 | 1 | 0.96 | 1 | 0.97 | 1 |
|  | dless | 0 | 0 | 0 | 0 | 0.87 | 0.93 | 0.94 | 1 | 0.97 | 1 |
| human – dusky titi | DivE | 0.83 | 0.96 | 0.93 | 0.99 | 0.94 | 1 | 0.98 | 1 | 0.99 | 1 |
|  | dless | 0.72 | 0.09 | 0.87 | 0.98 | 0.92 | 1 | 0.98 | 0.99 | 0.98 | 1 |
| mouse lemur | DivE | 0 | 0 | 0 | 0 | 0.32 | 0.11 | 0.82 | 0.98 | 0.92 | 1 |
|  | dless | 0 | 0 | 0 | 0 | 0.01 | 0 | 0.85 | 0.7 | 0.9 | 1 |
| galago | DivE | 0 | 0 | 0.46 | 0.02 | 0.78 | 0.85 | 0.93 | 1 | 0.97 | 1 |
|  | dless | 0 | 0 | 0 | 0 | 0 | 0 | 0.9 | 1 | 0.95 | 0.99 |
| mouse lemur - galago | DivE | 0.11 | 0.02 | 0.78 | 0.96 | 0.88 | 1 | 0.96 | 1 | 0.97 | 1 |
|  | dless | 0 | 0 | 0.71 | 0.01 | 0.87 | 1 | 0.95 | 1 | 0.96 | 1 |

**Table S2.** Accuracy obtained by DivE and DLESS for the prediction of gained elements of different lengths in each clade^1^ of the phylogeny, when the selction strength is given by ρ=0.02.

| 0.05 gain | | **50 bp** | | **100 bp** | | **200 bp** | | **500 bp** | | **1000 bp** | |
| --- | --- | --- | --- | --- | --- | --- | --- | --- | --- | --- | --- |
| **tree** | **prog** | Pr | Re | Pr | Re | Pr | Re | Pr | Re | Pr | Re |
| human | DivE | 0 | 0 | 0 | 0 | 0 | 0 | 0 | 0 | 0 | 0 |
|  | dless | 0 | 0 | 0 | 0 | 0 | 0 | 0.17 | 0.02 | 0.25 | 0.02 |
| chimp | DivE | 0 | 0 | 0 | 0 | 0 | 0 | 0 | 0 | 0 | 0 |
|  | dless | 0 | 0 | 0 | 0 | 0 | 0 | 0.17 | 0.01 | 0.25 | 0.01 |
| human -chimp | DivE | 0 | 0 | 0 | 0 | 0 | 0 | 0.48 | 0.07 | 0.84 | 0.98 |
|  | dless | 0 | 0 | 0 | 0 | 0 | 0 | 0.21 | 0.02 | 0.50 | 0.12 |
| orangutan | DivE | 0 | 0 | 0 | 0 | 0.02 | 0.00 | 0 | 0 | 0.83 | 0.89 |
|  | dless | 0 | 0 | 0 | 0 | 0 | 0 | 0 | 0 | 0.42 | 0.01 |
| human -orangutan | DivE | 0 | 0 | 0 | 0 | 0.40 | 0.05 | 0.88 | 0.98 | 0.91 | 0.99 |
|  | dless | 0 | 0 | 0 | 0 | 0.08 | 0.00 | 0.59 | 0.04 | 0.90 | 1.00 |
| gibbon | DivE | 0 | 0 | 0 | 0 | 0 | 0 | 0.35 | 0.02 | 0.88 | 0.97 |
|  | dless | 0 | 0 | 0 | 0 | 0 | 0 | 0 | 0 | 0.46 | 0.05 |
| human - gibbon | DivE | 0 | 0 | 0 | 0 | 0.66 | 0.51 | 0.91 | 0.99 | 0.95 | 1.00 |
|  | dless | 0 | 0 | 0 | 0 | 0.08 | 0.01 | 0.89 | 1.00 | 0.92 | 1.00 |
| colobus monkey | DivE | 0 | 0 | 0 | 0 | 0 | 0 | 0.34 | 0.02 | 0.79 | 0.82 |
|  | dless | 0 | 0 | 0 | 0 | 0 | 0 | 0 | 0 | 0.4 | 0.04 |
| vervet | DivE | 0 | 0 | 0 | 0 | 0 | 0 | 0 | 0 | 0.55 | 0.15 |
|  | dless | 0 | 0 | 0 | 0 | 0 | 0 | 0.17 | 0.01 | 0.25 | 0.02 |
| baboon | DivE | 0 | 0 | 0.05 | 0.01 | 0 | 0 | 0 | 0 | 0.42 | 0.03 |
|  | dless | 0 | 0 | 0 | 0 | 0 | 0 | 0 | 0 | 0.25 | 0.03 |
| macaque | DivE | 0 | 0 | 0 | 0 | 0 | 0 | 0 | 0 | 0.44 | 0.04 |
|  | dless | 0.01 | 0.01 | 0.05 | 0.01 | 0 | 0 | 0 | 0 | 0 | 0 |
| baboon - macaque | DivE | 0 | 0 | 0 | 0 | 0 | 0 | 0.61 | 0.1 | 0.88 | 1 |
|  | dless | 0 | 0 | 0.05 | 0.01 | 0 | 0 | 0 | 0 | 0.72 | 0.29 |
| vervet - baboon | DivE | 0 | 0 | 0 | 0 | 0 | 0 | 0.84 | 0.98 | 0.92 | 1 |
|  | dless | 0 | 0 | 0 | 0 | 0.08 | 0 | 0.68 | 0.03 | 0.9 | 0.99 |
| colobus monkey - vervet | DivE | 0 | 0 | 0.38 | 0.03 | 0.78 | 0.81 | 0.93 | 1 | 0.96 | 1 |
|  | dless | 0 | 0 | 0.05 | 0 | 0.08 | 0.01 | 0.89 | 1 | 0.95 | 1 |
| human - colobus monkey | DivE | 0 | 0 | 0.82 | 0.83 | 0.92 | 0.99 | 0.95 | 1 | 0.98 | 1 |
|  | dless | 0.02 | 0 | 0.28 | 0.01 | 0.88 | 0.97 | 0.95 | 1 | 0.97 | 0.99 |
| dusky titi | DivE | 0 | 0 | 0 | 0 | 0.16 | 0.01 | 0.75 | 0.55 | 0.9 | 0.99 |
|  | dless | 0 | 0 | 0 | 0 | 0 | 0 | 0 | 0 | 0.82 | 0.69 |
| owl monkey | DivE | 0 | 0 | 0 | 0 | 0 | 0 | 0.5 | 0.12 | 0.85 | 0.99 |
|  | dless | 0 | 0 | 0 | 0 | 0 | 0 | 0 | 0 | 0.55 | 0.07 |
| marmoset | DivE | 0 | 0 | 0 | 0 | 0 | 0 | 0.69 | 0.37 | 0.89 | 0.99 |
|  | dless | 0 | 0 | 0 | 0 | 0 | 0 | 0 | 0 | 0.8 | 0.59 |
| squirrel monkey | DivE | 0 | 0 | 0 | 0 | 0.31 | 0.01 | 0.72 | 0.53 | 0.88 | 0.99 |
|  | dless | 0 | 0 | 0 | 0 | 0 | 0 | 0.32 | 0.01 | 0.81 | 0.73 |
| marmoset - squirrel monkey | DivE | 0 | 0 | 0 | 0 | 0.62 | 0.17 | 0.91 | 0.99 | 0.94 | 0.99 |
|  | dless | 0 | 0 | 0 | 0 | 0 | 0 | 0.84 | 0.85 | 0.93 | 0.98 |
| owl monkey - marmoset | DivE | 0 | 0 | 0 | 0 | 0.8 | 0.88 | 0.92 | 1 | 0.96 | 1 |
|  | dless | 0.01 | 0 | 0 | 0 | 0 | 0 | 0.91 | 0.99 | 0.95 | 1 |
| dusky titi - owl monkey | DivE | 0.08 | 0.01 | 0.73 | 0.54 | 0.87 | 0.99 | 0.95 | 1 | 0.97 | 1 |
|  | dless | 0 | 0 | 0 | 0 | 0.81 | 0.75 | 0.95 | 0.98 | 0.97 | 0.99 |
| human – dusky titi | DivE | 0.81 | 0.79 | 0.88 | 0.99 | 0.95 | 1 | 0.98 | 1 | 0.99 | 1 |
|  | dless | 0.66 | 0.1 | 0.85 | 0.97 | 0.94 | 0.99 | 0.97 | 1 | 0.99 | 0.99 |
| mouse lemur | DivE | 0 | 0 | 0 | 0 | 0.47 | 0.08 | 0.88 | 0.99 | 0.93 | 1 |
|  | dless | 0 | 0 | 0 | 0 | 0 | 0 | 0.76 | 0.49 | 0.93 | 0.99 |
| galago | DivE | 0 | 0 | 0 | 0 | 0.74 | 0.76 | 0.89 | 0.99 | 0.96 | 1 |
|  | dless | 0 | 0 | 0 | 0 | 0.63 | 0.01 | 0.89 | 1 | 0.94 | 1 |
| mouse lemur - galago | DivE | 0.28 | 0.01 | 0.7 | 0.8 | 0.91 | 0.99 | 0.96 | 1 | 0.98 | 1 |
|  | dless | 0 | 0 | 0.62 | 0.01 | 0.83 | 0.98 | 0.95 | 0.99 | 0.97 | 1 |

**Table S3.** Accuracy obtained by DivE and DLESS for the prediction of gained elements of different lengths in each clade^1^ of the phylogeny, when the selction strength is given by ρ=0.05.

| 0.1 gain | | **50 bp** | | **100 bp** | | **200 bp** | | **500 bp** | | **1000 bp** | |
| --- | --- | --- | --- | --- | --- | --- | --- | --- | --- | --- | --- |
| **tree** | **prog** | Pr | Re | Pr | Re | Pr | Re | Pr | Re | Pr | Re |
| human | DivE | 0 | 0 | 0 | 0 | 0 | 0 | 0 | 0 | 0 | 0 |
|  | dless | 0 | 0 | 0 | 0 | 0 | 0 | 0 | 0 | 0 | 0 |
| chimp | DivE | 0 | 0 | 0 | 0 | 0 | 0 | 0 | 0 | 0 | 0 |
|  | dless | 0 | 0 | 0 | 0 | 0 | 0 | 0.17 | 0.01 | 0 | 0 |
| human -chimp | DivE | 0 | 0 | 0 | 0 | 0 | 0 | 0.52 | 0.10 | 0.87 | 0.88 |
|  | dless | 0 | 0 | 0.05 | 0.01 | 0 | 0 | 0 | 0 | 0.43 | 0.06 |
| orangutan | DivE | 0 | 0 | 0 | 0 | 0 | 0 | 0 | 0 | 0.78 | 0.59 |
|  | dless | 0 | 0 | 0 | 0 | 0 | 0 | 0 | 0 | 0.38 | 0.03 |
| human -orangutan | DivE | 0 | 0 | 0 | 0 | 0 | 0 | 0.85 | 0.85 | 0.90 | 0.99 |
|  | dless | 0 | 0 | 0.02 | 0.00 | 0 | 0 | 0.69 | 0.01 | 0.87 | 1.00 |
| gibbon | DivE | 0 | 0 | 0 | 0 | 0 | 0 | 0.49 | 0.04 | 0.85 | 0.88 |
|  | dless | 0 | 0 | 0 | 0 | 0 | 0 | 0.17 | 0.01 | 0.31 | 0.03 |
| human - gibbon | DivE | 0 | 0 | 0.20 | 0.01 | 0.64 | 0.29 | 0.91 | 0.98 | 0.94 | 0.99 |
|  | dless | 0 | 0 | 0.05 | 0.00 | 0.08 | 0.01 | 0.91 | 0.95 | 0.94 | 1.00 |
| colobus monkey | DivE | 0 | 0 | 0 | 0 | 0 | 0 | 0.22 | 0.02 | 0.78 | 0.58 |
|  | dless | 0 | 0 | 0 | 0 | 0 | 0 | 0 | 0 | 0.32 | 0.02 |
| vervet | DivE | 0 | 0 | 0 | 0 | 0 | 0 | 0 | 0 | 0.63 | 0.06 |
|  | dless | 0 | 0 | 0 | 0 | 0 | 0 | 0 | 0 | 0 | 0 |
| baboon | DivE | 0 | 0 | 0 | 0 | 0 | 0 | 0 | 0 | 0.27 | 0.02 |
|  | dless | 0 | 0 | 0.05 | 0.01 | 0 | 0 | 0.17 | 0.01 | 0.25 | 0.01 |
| macaque | DivE | 0 | 0 | 0 | 0 | 0 | 0 | 0 | 0 | 0.4 | 0.02 |
|  | dless | 0 | 0 | 0 | 0 | 0 | 0 | 0.17 | 0.01 | 0.25 | 0.01 |
| baboon - macaque | DivE | 0 | 0 | 0 | 0 | 0 | 0 | 0.63 | 0.11 | 0.88 | 0.95 |
|  | dless | 0 | 0 | 0 | 0 | 0 | 0 | 0 | 0 | 0.74 | 0.14 |
| vervet - baboon | DivE | 0 | 0 | 0 | 0 | 0.38 | 0.03 | 0.85 | 0.92 | 0.92 | 0.99 |
|  | dless | 0.01 | 0 | 0 | 0 | 0 | 0 | 0.43 | 0.04 | 0.88 | 1 |
| colobus monkey - vervet | DivE | 0 | 0 | 0.17 | 0.01 | 0.8 | 0.6 | 0.91 | 0.99 | 0.94 | 0.99 |
|  | dless | 0 | 0 | 0 | 0 | 0.29 | 0.01 | 0.89 | 0.98 | 0.93 | 1 |
| human - colobus monkey | DivE | 0.44 | 0.03 | 0.78 | 0.71 | 0.9 | 0.98 | 0.97 | 0.99 | 0.98 | 1 |
|  | dless | 0 | 0 | 0 | 0 | 0.89 | 0.87 | 0.96 | 1 | 0.97 | 1 |
| dusky titi | DivE | 0 | 0 | 0 | 0 | 0 | 0 | 0.73 | 0.35 | 0.9 | 0.98 |
|  | dless | 0 | 0 | 0 | 0 | 0 | 0 | 0 | 0 | 0.76 | 0.41 |
| owl monkey | DivE | 0 | 0 | 0 | 0 | 0 | 0 | 0.43 | 0.07 | 0.88 | 0.94 |
|  | dless | 0 | 0 | 0 | 0 | 0 | 0 | 0 | 0 | 0.62 | 0.05 |
| marmoset | DivE | 0 | 0 | 0 | 0 | 0 | 0 | 0.63 | 0.24 | 0.89 | 0.96 |
|  | dless | 0 | 0 | 0 | 0 | 0 | 0 | 0 | 0 | 0.78 | 0.36 |
| squirrel monkey | DivE | 0 | 0 | 0 | 0 | 0 | 0 | 0.66 | 0.29 | 0.88 | 0.98 |
|  | dless | 0 | 0 | 0 | 0 | 0 | 0 | 0 | 0 | 0.78 | 0.52 |
| marmoset - squirrel monkey | DivE | 0 | 0 | 0 | 0 | 0.6 | 0.13 | 0.88 | 0.98 | 0.94 | 0.99 |
|  | dless | 0 | 0 | 0 | 0 | 0 | 0 | 0.81 | 0.61 | 0.92 | 1 |
| owl monkey - marmoset | DivE | 0.09 | 0.01 | 0 | 0 | 0.79 | 0.72 | 0.93 | 0.99 | 0.96 | 0.99 |
|  | dless | 0 | 0 | 0 | 0 | 0 | 0 | 0.89 | 1 | 0.94 | 1 |
| dusky titi - owl monkey | DivE | 0.27 | 0.01 | 0.61 | 0.34 | 0.89 | 0.98 | 0.94 | 0.99 | 0.97 | 0.99 |
|  | dless | 0 | 0 | 0 | 0 | 0.85 | 0.5 | 0.93 | 1 | 0.97 | 1 |
| human – dusky titi | DivE | 0.71 | 0.64 | 0.89 | 0.99 | 0.95 | 0.99 | 0.99 | 0.99 | 0.99 | 1 |
|  | dless | 0.45 | 0.02 | 0.85 | 0.92 | 0.93 | 0.99 | 0.98 | 1 | 0.99 | 1 |
| mouse lemur | DivE | 0 | 0 | 0 | 0 | 0.49 | 0.04 | 0.87 | 0.95 | 0.94 | 0.98 |
|  | dless | 0 | 0 | 0 | 0 | 0 | 0 | 0.82 | 0.25 | 0.93 | 0.99 |
| galago | DivE | 0 | 0 | 0.28 | 0.01 | 0.75 | 0.5 | 0.9 | 0.98 | 0.94 | 0.99 |
|  | dless | 0 | 0 | 0 | 0 | 0.47 | 0.01 | 0.9 | 0.98 | 0.93 | 1 |
| mouse lemur - galago | DivE | 0.23 | 0.02 | 0.73 | 0.61 | 0.89 | 0.98 | 0.95 | 1 | 0.97 | 1 |
|  | dless | 0 | 0 | 0.02 | 0.01 | 0.86 | 0.82 | 0.95 | 0.99 | 0.97 | 0.99 |

**Table S4.** Accuracy obtained by DivE and DLESS for the prediction of gained elements of different lengths in each clade^1^ of the phylogeny, when the selction strength is given by ρ=0.1.

| 0.2 gain | | **50 bp** | | **100 bp** | | **200 bp** | | **500 bp** | | **1000 bp** | |
| --- | --- | --- | --- | --- | --- | --- | --- | --- | --- | --- | --- |
| **tree** | **prog** | Pr | Re | Pr | Re | Pr | Re | Pr | Re | Pr | Re |
| human | DivE | 0 | 0 | 0 | 0 | 0 | 0 | 0.09 | 0.01 | 0.13 | 0.00 |
|  | dless | 0 | 0 | 0 | 0 | 0 | 0 | 0 | 0 | 0 | 0 |
| chimp | DivE | 0 | 0 | 0 | 0 | 0 | 0 | 0 | 0 | 0 | 0 |
|  | dless | 0.02 | 0.01 | 0 | 0 | 0 | 0 | 0 | 0 | 0 | 0 |
| human -chimp | DivE | 0 | 0 | 0 | 0 | 0 | 0 | 0.58 | 0.02 | 0.84 | 0.58 |
|  | dless | 0.02 | 0.01 | 0 | 0 | 0.08 | 0.01 | 0 | 0 | 0.62 | 0.02 |
| orangutan | DivE | 0 | 0 | 0 | 0 | 0 | 0 | 0 | 0 | 0.70 | 0.24 |
|  | dless | 0 | 0 | 0 | 0 | 0 | 0 | 0 | 0 | 0 | 0 |
| human -orangutan | DivE | 0 | 0 | 0 | 0 | 0.52 | 0.01 | 0.82 | 0.41 | 0.91 | 0.95 |
|  | dless | 0.01 | 0 | 0 | 0 | 0 | 0 | 0.35 | 0.02 | 0.90 | 0.76 |
| gibbon | DivE | 0 | 0 | 0 | 0 | 0 | 0 | 0.33 | 0.01 | 0.84 | 0.45 |
|  | dless | 0 | 0 | 0 | 0 | 0 | 0 | 0 | 0 | 0.64 | 0.02 |
| human - gibbon | DivE | 0 | 0 | 0 | 0 | 0.56 | 0.10 | 0.91 | 0.95 | 0.95 | 0.98 |
|  | dless | 0 | 0 | 0 | 0 | 0.08 | 0.00 | 0.84 | 0.53 | 0.93 | 0.98 |
| colobus monkey | DivE | 0 | 0 | 0 | 0 | 0 | 0 | 0 | 0 | 0.76 | 0.24 |
|  | dless | 0 | 0 | 0 | 0 | 0 | 0 | 0 | 0 | 0 | 0 |
| vervet | DivE | 0 | 0 | 0 | 0 | 0 | 0 | 0.19 | 0.01 | 0.54 | 0.06 |
|  | dless | 0 | 0 | 0 | 0 | 0 | 0 | 0 | 0 | 0 | 0 |
| baboon | DivE | 0 | 0 | 0 | 0 | 0 | 0 | 0 | 0 | 0.21 | 0.01 |
|  | dless | 0 | 0 | 0 | 0 | 0 | 0 | 0 | 0 | 0.4 | 0.01 |
| macaque | DivE | 0 | 0 | 0 | 0 | 0 | 0 | 0 | 0 | 0 | 0 |
|  | dless | 0 | 0 | 0 | 0 | 0 | 0 | 0 | 0 | 0.25 | 0.01 |
| baboon - macaque | DivE | 0 | 0 | 0 | 0 | 0 | 0 | 0.44 | 0.06 | 0.82 | 0.64 |
|  | dless | 0 | 0 | 0 | 0 | 0 | 0 | 0.17 | 0.01 | 0.4 | 0.05 |
| vervet - baboon | DivE | 0 | 0 | 0 | 0 | 0 | 0 | 0.82 | 0.54 | 0.89 | 0.95 |
|  | dless | 0 | 0 | 0 | 0 | 0 | 0 | 0.38 | 0.01 | 0.89 | 0.77 |
| colobus monkey - vervet | DivE | 0 | 0 | 0.28 | 0.01 | 0.78 | 0.2 | 0.91 | 0.93 | 0.96 | 0.98 |
|  | dless | 0 | 0 | 0 | 0 | 0 | 0 | 0.91 | 0.85 | 0.95 | 0.98 |
| human - colobus monkey | DivE | 0 | 0 | 0.72 | 0.3 | 0.9 | 0.91 | 0.96 | 0.98 | 0.98 | 0.99 |
|  | dless | 0 | 0 | 0.05 | 0 | 0.82 | 0.45 | 0.96 | 0.99 | 0.98 | 1 |
| dusky titi | DivE | 0 | 0 | 0 | 0 | 0 | 0 | 0.6 | 0.1 | 0.89 | 0.87 |
|  | dless | 0 | 0 | 0 | 0 | 0 | 0 | 0 | 0 | 0.67 | 0.1 |
| owl monkey | DivE | 0 | 0 | 0 | 0 | 0 | 0 | 0.38 | 0.04 | 0.84 | 0.71 |
|  | dless | 0 | 0 | 0 | 0 | 0 | 0 | 0 | 0 | 0.47 | 0.03 |
| marmoset | DivE | 0 | 0 | 0 | 0 | 0 | 0 | 0.59 | 0.07 | 0.88 | 0.82 |
|  | dless | 0 | 0 | 0 | 0 | 0 | 0 | 0.11 | 0.01 | 0.71 | 0.1 |
| squirrel monkey | DivE | 0 | 0 | 0 | 0 | 0 | 0 | 0.65 | 0.06 | 0.87 | 0.78 |
|  | dless | 0 | 0 | 0 | 0 | 0 | 0 | 0.26 | 0.01 | 0.73 | 0.08 |
| marmoset - squirrel monkey | DivE | 0 | 0 | 0 | 0 | 0.41 | 0.04 | 0.87 | 0.81 | 0.93 | 0.97 |
|  | dless | 0 | 0 | 0 | 0 | 0 | 0 | 0.82 | 0.19 | 0.93 | 0.97 |
| owl monkey - marmoset | DivE | 0 | 0 | 0 | 0 | 0.77 | 0.21 | 0.91 | 0.94 | 0.96 | 0.98 |
|  | dless | 0 | 0 | 0 | 0 | 0 | 0 | 0.9 | 0.82 | 0.95 | 0.99 |
| dusky titi - owl monkey | DivE | 0 | 0 | 0.42 | 0.1 | 0.87 | 0.81 | 0.95 | 0.96 | 0.98 | 0.98 |
|  | dless | 0 | 0 | 0 | 0 | 0.78 | 0.21 | 0.95 | 0.98 | 0.97 | 0.98 |
| human – dusky titi | DivE | 0.82 | 0.22 | 0.87 | 0.9 | 0.95 | 0.97 | 0.98 | 0.99 | 0.99 | 0.99 |
|  | dless | 0.73 | 0.02 | 0.84 | 0.44 | 0.95 | 0.99 | 0.97 | 1 | 0.99 | 1 |
| mouse lemur | DivE | 0 | 0 | 0 | 0 | 0.26 | 0.01 | 0.89 | 0.72 | 0.94 | 0.94 |
|  | dless | 0 | 0 | 0 | 0 | 0 | 0 | 0.92 | 0.02 | 0.93 | 0.92 |
| galago | DivE | 0 | 0 | 0 | 0 | 0.57 | 0.14 | 0.93 | 0.93 | 0.94 | 0.97 |
|  | dless | 0 | 0 | 0 | 0 | 0 | 0 | 0.84 | 0.7 | 0.95 | 0.98 |
| mouse lemur - galago | DivE | 0 | 0 | 0.75 | 0.2 | 0.91 | 0.9 | 0.96 | 0.97 | 0.97 | 0.99 |
|  | dless | 0 | 0 | 0 | 0 | 0.71 | 0.38 | 0.95 | 0.99 | 0.97 | 0.99 |

**Table S5.** Accuracy obtained by DivE and DLESS for the prediction of gained elements of different lengths in each clade^1^ of the phylogeny, when the selction strength is given by ρ=0.2.

| 0.3 gain | | **50 bp** | | **100 bp** | | **200 bp** | | **500 bp** | | **1000 bp** | |
| --- | --- | --- | --- | --- | --- | --- | --- | --- | --- | --- | --- |
| **tree** | **prog** | Pr | Re | Pr | Re | Pr | Re | Pr | Re | Pr | Re |
| human | DivE | 0 | 0 | 0 | 0 | 0 | 0 | 0 | 0 | 0.07 | 0 |
|  | dless | 0 | 0 | 0 | 0 | 0 | 0 | 0 | 0 | 0.25 | 0.01 |
| chimp | DivE | 0 | 0 | 0.01 | 0.01 | 0 | 0 | 0 | 0 | 0 | 0 |
|  | dless | 0 | 0 | 0 | 0 | 0 | 0 | 0.17 | 0.01 | 0.25 | 0.01 |
| human -chimp | DivE | 0 | 0 | 0 | 0 | 0 | 0 | 0 | 0 | 0.82 | 0.28 |
|  | dless | 0 | 0 | 0.05 | 0.01 | 0 | 0 | 0.17 | 0.01 | 0.97 | 0.01 |
| orangutan | DivE | 0 | 0 | 0 | 0 | 0 | 0 | 0 | 0 | 0.50 | 0.06 |
|  | dless | 0 | 0 | 0 | 0 | 0 | 0 | 0 | 0 | 0.25 | 0.01 |
| human -orangutan | DivE | 0 | 0 | 0 | 0 | 0 | 0 | 0.73 | 0.30 | 0.91 | 0.75 |
|  | dless | 0.02 | 0.01 | 0 | 0 | 0 | 0 | 0 | 0 | 0.86 | 0.18 |
| gibbon | DivE | 0 | 0 | 0 | 0 | 0 | 0 | 0.25 | 0.01 | 0.77 | 0.21 |
|  | dless | 0 | 0 | 0 | 0 | 0 | 0 | 0 | 0 | 0.37 | 0.01 |
| human - gibbon | DivE | 0 | 0 | 0 | 0 | 0.58 | 0.05 | 0.82 | 0.70 | 0.93 | 0.93 |
|  | dless | 0 | 0 | 0 | 0 | 0 | 0 | 0.84 | 0.12 | 0.95 | 0.86 |
| colobus monkey | DivE | 0 | 0 | 0 | 0 | 0 | 0 | 0 | 0 | 0.28 | 0.01 |
|  | dless | 0 | 0 | 0 | 0 | 0 | 0 | 0 | 0 | 0.34 | 0.01 |
| vervet | DivE | 0 | 0 | 0 | 0 | 0 | 0 | 0 | 0 | 0.44 | 0.03 |
|  | dless | 0 | 0 | 0 | 0 | 0 | 0 | 0 | 0 | 0 | 0 |
| baboon | DivE | 0 | 0 | 0 | 0 | 0 | 0 | 0 | 0 | 0 | 0 |
|  | dless | 0 | 0 | 0 | 0 | 0 | 0 | 0 | 0 | 0.25 | 0.01 |
| macaque | DivE | 0 | 0 | 0 | 0 | 0 | 0 | 0 | 0 | 0.4 | 0.02 |
|  | dless | 0 | 0 | 0 | 0 | 0.08 | 0.01 | 0.17 | 0.01 | 0 | 0 |
| baboon - macaque | DivE | 0 | 0 | 0 | 0 | 0.48 | 0.01 | 0.44 | 0.03 | 0.79 | 0.38 |
|  | dless | 0 | 0 | 0 | 0 | 0 | 0 | 0.17 | 0.01 | 0.37 | 0.03 |
| vervet - baboon | DivE | 0 | 0 | 0 | 0 | 0 | 0 | 0.89 | 0.28 | 0.92 | 0.79 |
|  | dless | 0.02 | 0 | 0 | 0 | 0 | 0 | 0.17 | 0 | 0.85 | 0.27 |
| colobus monkey - vervet | DivE | 0 | 0 | 0 | 0 | 0.41 | 0.03 | 0.91 | 0.78 | 0.96 | 0.95 |
|  | dless | 0 | 0 | 0 | 0 | 0 | 0 | 0.89 | 0.28 | 0.95 | 0.95 |
| human - colobus monkey | DivE | 0 | 0 | 0.66 | 0.1 | 0.85 | 0.68 | 0.94 | 0.95 | 0.97 | 0.98 |
|  | dless | 0 | 0 | 0 | 0 | 0.8 | 0.14 | 0.96 | 0.96 | 0.98 | 0.99 |
| dusky titi | DivE | 0 | 0 | 0 | 0 | 0 | 0 | 0.68 | 0.03 | 0.86 | 0.39 |
|  | dless | 0 | 0 | 0 | 0 | 0 | 0 | 0 | 0 | 0 | 0 |
| owl monkey | DivE | 0 | 0 | 0 | 0 | 0 | 0 | 0 | 0 | 0.75 | 0.31 |
|  | dless | 0 | 0 | 0 | 0 | 0 | 0 | 0 | 0 | 0 | 0 |
| marmoset | DivE | 0 | 0 | 0 | 0 | 0 | 0 | 0.53 | 0.03 | 0.85 | 0.46 |
|  | dless | 0 | 0 | 0 | 0 | 0 | 0 | 0 | 0 | 0.61 | 0.02 |
| squirrel monkey | DivE | 0 | 0 | 0 | 0 | 0 | 0 | 0.39 | 0.02 | 0.82 | 0.52 |
|  | dless | 0 | 0 | 0 | 0 | 0 | 0 | 0 | 0 | 0.6 | 0.04 |
| marmoset - squirrel monkey | DivE | 0 | 0 | 0 | 0 | 0.3 | 0.01 | 0.73 | 0.43 | 0.93 | 0.91 |
|  | dless | 0 | 0 | 0 | 0 | 0 | 0 | 0.69 | 0.02 | 0.93 | 0.74 |
| owl monkey - marmoset | DivE | 0 | 0 | 0 | 0 | 0.56 | 0.04 | 0.9 | 0.87 | 0.94 | 0.95 |
|  | dless | 0 | 0 | 0 | 0 | 0 | 0 | 0.88 | 0.36 | 0.95 | 0.97 |
| dusky titi - owl monkey | DivE | 0 | 0 | 0.45 | 0.04 | 0.81 | 0.43 | 0.93 | 0.94 | 0.97 | 0.97 |
|  | dless | 0 | 0 | 0 | 0 | 0.8 | 0.01 | 0.95 | 0.92 | 0.97 | 0.99 |
| human – dusky titi | DivE | 0.74 | 0.05 | 0.86 | 0.59 | 0.94 | 0.94 | 0.97 | 0.98 | 0.98 | 0.99 |
|  | dless | 0.02 | 0 | 0.84 | 0.15 | 0.95 | 0.9 | 0.98 | 0.97 | 0.99 | 0.99 |
| mouse lemur | DivE | 0 | 0 | 0.05 | 0 | 0.33 | 0.01 | 0.82 | 0.4 | 0.87 | 0.87 |
|  | dless | 0 | 0 | 0 | 0 | 0 | 0 | 0.8 | 0.02 | 0.93 | 0.56 |
| galago | DivE | 0 | 0 | 0 | 0 | 0.66 | 0.07 | 0.89 | 0.72 | 0.94 | 0.95 |
|  | dless | 0 | 0 | 0 | 0 | 0 | 0 | 0.89 | 0.26 | 0.96 | 0.95 |
| mouse lemur - galago | DivE | 0.3 | 0.02 | 0.59 | 0.07 | 0.82 | 0.49 | 0.95 | 0.94 | 0.97 | 0.97 |
|  | dless | 0 | 0 | 0 | 0 | 0.84 | 0.11 | 0.97 | 0.93 | 0.98 | 0.98 |

**Table S6.** Accuracy obtained by DivE and DLESS for the prediction of gained elements of different lengths in each clade^1^ of the phylogeny, when the selction strength is given by ρ=0.3.

| 0.4 gain | | **50 bp** | | **100 bp** | | **200 bp** | | **500 bp** | | **1000 bp** | |
| --- | --- | --- | --- | --- | --- | --- | --- | --- | --- | --- | --- |
| **tree** | **prog** | Pr | Re | Pr | Re | Pr | Re | Pr | Re | Pr | Re |
| human | DivE | 0 | 0 | 0 | 0 | 0 | 0 | 0 | 0 | 0 | 0 |
|  | dless | 0 | 0 | 0 | 0 | 0 | 0 | 0 | 0 | 0.20 | 0.01 |
| chimp | DivE | 0 | 0 | 0 | 0 | 0 | 0 | 0 | 0 | 0 | 0 |
|  | dless | 0 | 0 | 0 | 0 | 0.04 | 0.01 | 0 | 0 | 0.25 | 0.01 |
| human -chimp | DivE | 0 | 0 | 0 | 0 | 0 | 0 | 0.36 | 0.01 | 0.60 | 0.07 |
|  | dless | 0.02 | 0.01 | 0 | 0 | 0 | 0 | 0.24 | 0.02 | 0.25 | 0.01 |
| orangutan | DivE | 0 | 0 | 0 | 0 | 0 | 0 | 0 | 0 | 0.70 | 0.06 |
|  | dless | 0.01 | 0.01 | 0 | 0 | 0 | 0 | 0 | 0 | 0 | 0 |
| human -orangutan | DivE | 0 | 0 | 0 | 0 | 0 | 0 | 0.64 | 0.02 | 0.86 | 0.51 |
|  | dless | 0.02 | 0.00 | 0 | 0 | 0 | 0 | 0.17 | 0.00 | 0.73 | 0.06 |
| gibbon | DivE | 0 | 0 | 0 | 0 | 0 | 0 | 0 | 0 | 0.76 | 0.07 |
|  | dless | 0 | 0 | 0 | 0 | 0 | 0 | 0 | 0 | 0.35 | 0.01 |
| human - gibbon | DivE | 0 | 0 | 0 | 0 | 0.48 | 0.02 | 0.84 | 0.30 | 0.93 | 0.83 |
|  | dless | 0 | 0 | 0 | 0 | 0 | 0 | 0.58 | 0.04 | 0.92 | 0.57 |
| colobus monkey | DivE | 0 | 0 | 0 | 0 | 0 | 0 | 0 | 0 | 0 | 0 |
|  | dless | 0.02 | 0.01 | 0 | 0 | 0 | 0 | 0 | 0 | 0 | 0 |
| vervet | DivE | 0 | 0 | 0 | 0 | 0 | 0 | 0 | 0 | 0.3 | 0.01 |
|  | dless | 0 | 0 | 0 | 0 | 0 | 0 | 0 | 0 | 0.25 | 0.01 |
| baboon | DivE | 0 | 0 | 0 | 0 | 0 | 0 | 0 | 0 | 0 | 0 |
|  | dless | 0 | 0 | 0.02 | 0.01 | 0 | 0 | 0 | 0 | 0 | 0 |
| macaque | DivE | 0 | 0 | 0 | 0 | 0 | 0 | 0 | 0 | 0 | 0 |
|  | dless | 0 | 0 | 0 | 0 | 0 | 0 | 0 | 0 | 0 | 0 |
| baboon - macaque | DivE | 0 | 0 | 0 | 0 | 0 | 0 | 0 | 0 | 0.86 | 0.17 |
|  | dless | 0 | 0 | 0 | 0 | 0 | 0 | 0 | 0 | 0 | 0 |
| vervet - baboon | DivE | 0 | 0 | 0 | 0 | 0 | 0 | 0.74 | 0.08 | 0.87 | 0.44 |
|  | dless | 0 | 0 | 0 | 0 | 0 | 0 | 0 | 0 | 0.89 | 0.06 |
| colobus monkey - vervet | DivE | 0 | 0 | 0 | 0 | 0.68 | 0.03 | 0.85 | 0.54 | 0.94 | 0.88 |
|  | dless | 0.02 | 0 | 0 | 0 | 0 | 0 | 0.69 | 0.07 | 0.96 | 0.69 |
| human - colobus monkey | DivE | 0 | 0 | 0.81 | 0.02 | 0.83 | 0.27 | 0.95 | 0.84 | 0.96 | 0.96 |
|  | dless | 0 | 0 | 0 | 0 | 0.55 | 0.02 | 0.96 | 0.64 | 0.97 | 0.93 |
| dusky titi | DivE | 0 | 0 | 0 | 0 | 0 | 0 | 0.77 | 0.02 | 0.84 | 0.16 |
|  | dless | 0 | 0 | 0 | 0 | 0 | 0 | 0 | 0 | 0 | 0 |
| owl monkey | DivE | 0 | 0 | 0 | 0 | 0 | 0 | 0 | 0 | 0.71 | 0.07 |
|  | dless | 0 | 0 | 0 | 0 | 0 | 0 | 0 | 0 | 0 | 0 |
| marmoset | DivE | 0 | 0 | 0 | 0 | 0 | 0 | 0 | 0 | 0.87 | 0.15 |
|  | dless | 0 | 0 | 0 | 0 | 0 | 0 | 0 | 0 | 0 | 0 |
| squirrel monkey | DivE | 0 | 0 | 0 | 0 | 0 | 0 | 0.6 | 0.04 | 0.78 | 0.2 |
|  | dless | 0 | 0 | 0 | 0 | 0 | 0 | 0 | 0 | 0 | 0 |
| marmoset - squirrel monkey | DivE | 0 | 0 | 0 | 0 | 0 | 0 | 0.7 | 0.14 | 0.9 | 0.75 |
|  | dless | 0 | 0 | 0 | 0 | 0.12 | 0.01 | 0.58 | 0.01 | 0.88 | 0.24 |
| owl monkey - marmoset | DivE | 0 | 0 | 0.15 | 0 | 0.74 | 0.01 | 0.85 | 0.49 | 0.93 | 0.86 |
|  | dless | 0 | 0 | 0 | 0 | 0 | 0 | 0.9 | 0.07 | 0.95 | 0.63 |
| dusky titi - owl monkey | DivE | 0 | 0 | 0 | 0 | 0.68 | 0.17 | 0.91 | 0.84 | 0.96 | 0.93 |
|  | dless | 0 | 0 | 0 | 0 | 0.25 | 0.01 | 0.93 | 0.39 | 0.98 | 0.92 |
| human – dusky titi | DivE | 0.62 | 0.04 | 0.81 | 0.28 | 0.91 | 0.74 | 0.98 | 0.95 | 0.98 | 0.98 |
|  | dless | 0.02 | 0 | 0.85 | 0.03 | 0.92 | 0.42 | 0.98 | 0.95 | 0.99 | 0.96 |
| mouse lemur | DivE | 0 | 0 | 0 | 0 | 0 | 0 | 0.79 | 0.21 | 0.89 | 0.65 |
|  | dless | 0 | 0 | 0 | 0 | 0 | 0 | 0.79 | 0.01 | 0.75 | 0.16 |
| galago | DivE | 0 | 0 | 0 | 0 | 0.44 | 0.03 | 0.89 | 0.42 | 0.94 | 0.89 |
|  | dless | 0 | 0 | 0 | 0 | 0 | 0 | 0.5 | 0.07 | 0.95 | 0.57 |
| mouse lemur - galago | DivE | 0 | 0 | 0.63 | 0.03 | 0.84 | 0.18 | 0.95 | 0.87 | 0.97 | 0.95 |
|  | dless | 0 | 0 | 0 | 0 | 0.67 | 0.01 | 0.96 | 0.64 | 0.98 | 0.94 |

**Table S7.** Accuracy obtained by DivE and DLESS for the prediction of gained elements of different lengths in each clade^1^ of the phylogeny, when the selction strength is given by ρ=0.4.

| 0.5 gain | | **50 bp** | | **100 bp** | | **200 bp** | | **500 bp** | | **1000 bp** | |
| --- | --- | --- | --- | --- | --- | --- | --- | --- | --- | --- | --- |
| **tree** | **prog** | Pr | Re | Pr | Re | Pr | Re | Pr | Re | Pr | Re |
| human | DivE | 0 | 0 | 0 | 0 | 0 | 0 | 0 | 0 | 0 | 0 |
|  | dless | 0.02 | 0.01 | 0.05 | 0.01 | 0.08 | 0.01 | 0 | 0 | 0 | 0 |
| chimp | DivE | 0 | 0 | 0 | 0 | 0 | 0 | 0 | 0 | 0 | 0 |
|  | dless | 0.02 | 0.01 | 0 | 0 | 0.08 | 0.01 | 0 | 0 | 0 | 0 |
| human -chimp | DivE | 0 | 0 | 0 | 0 | 0 | 0 | 0 | 0 | 0.52 | 0.02 |
|  | dless | 0 | 0 | 0 | 0 | 0 | 0 | 0.17 | 0.01 | 0.25 | 0.01 |
| orangutan | DivE | 0 | 0 | 0 | 0 | 0 | 0 | 0 | 0 | 0 | 0 |
|  | dless | 0 | 0 | 0 | 0 | 0 | 0 | 0 | 0 | 0 | 0 |
| human -orangutan | DivE | 0 | 0 | 0 | 0 | 0.20 | 0.01 | 0 | 0 | 0.89 | 0.19 |
|  | dless | 0 | 0 | 0 | 0 | 0.08 | 0.01 | 0 | 0 | 0 | 0 |
| gibbon | DivE | 0 | 0 | 0 | 0 | 0.08 | 0.01 | 0 | 0 | 0.95 | 0.01 |
|  | dless | 0 | 0 | 0 | 0 | 0 | 0 | 0 | 0 | 0 | 0 |
| human - gibbon | DivE | 0 | 0 | 0 | 0 | 0.46 | 0.01 | 0.91 | 0.10 | 0.93 | 0.56 |
|  | dless | 0 | 0 | 0 | 0 | 0 | 0 | 0.31 | 0.01 | 0.98 | 0.10 |
| colobus monkey | DivE | 0 | 0 | 0 | 0 | 0 | 0 | 0 | 0 | 0 | 0 |
|  | dless | 0 | 0 | 0 | 0 | 0 | 0 | 0 | 0 | 0.25 | 0.01 |
| vervet | DivE | 0 | 0 | 0 | 0 | 0 | 0 | 0 | 0 | 0 | 0 |
|  | dless | 0 | 0 | 0 | 0 | 0 | 0 | 0 | 0 | 0 | 0 |
| baboon | DivE | 0 | 0 | 0 | 0 | 0 | 0 | 0 | 0 | 0.26 | 0.01 |
|  | dless | 0 | 0 | 0 | 0 | 0 | 0 | 0 | 0 | 0 | 0 |
| macaque | DivE | 0 | 0 | 0 | 0 | 0 | 0 | 0 | 0 | 0 | 0 |
|  | dless | 0 | 0 | 0 | 0 | 0 | 0 | 0 | 0 | 0 | 0 |
| baboon - macaque | DivE | 0 | 0 | 0 | 0 | 0 | 0 | 0.67 | 0.01 | 0.61 | 0.06 |
|  | dless | 0 | 0 | 0 | 0 | 0 | 0 | 0 | 0 | 0 | 0 |
| vervet - baboon | DivE | 0 | 0 | 0 | 0 | 0 | 0 | 0.28 | 0 | 0.91 | 0.15 |
|  | dless | 0 | 0 | 0 | 0 | 0 | 0 | 0.17 | 0.01 | 0.86 | 0.01 |
| colobus monkey - vervet | DivE | 0 | 0 | 0 | 0 | 0.51 | 0.02 | 0.83 | 0.11 | 0.92 | 0.69 |
|  | dless | 0.02 | 0 | 0 | 0 | 0 | 0 | 0 | 0 | 0.95 | 0.15 |
| human - colobus monkey | DivE | 0 | 0 | 0 | 0 | 0.82 | 0.1 | 0.95 | 0.64 | 0.95 | 0.87 |
|  | dless | 0 | 0 | 0 | 0 | 0.81 | 0.01 | 0.95 | 0.21 | 0.96 | 0.53 |
| dusky titi | DivE | 0 | 0 | 0 | 0 | 0 | 0 | 0 | 0 | 0.99 | 0.02 |
|  | dless | 0 | 0 | 0 | 0 | 0 | 0 | 0 | 0 | 0 | 0 |
| owl monkey | DivE | 0 | 0 | 0 | 0 | 0 | 0 | 0 | 0 | 0.6 | 0.04 |
|  | dless | 0 | 0 | 0 | 0 | 0 | 0 | 0 | 0 | 0 | 0 |
| marmoset | DivE | 0 | 0 | 0 | 0 | 0.17 | 0.01 | 0.5 | 0.01 | 0.78 | 0.01 |
|  | dless | 0 | 0 | 0 | 0 | 0 | 0 | 0 | 0 | 0 | 0 |
| squirrel monkey | DivE | 0 | 0 | 0 | 0 | 0.06 | 0.01 | 0 | 0 | 0.69 | 0.04 |
|  | dless | 0 | 0 | 0 | 0 | 0 | 0 | 0 | 0 | 0.44 | 0.01 |
| marmoset - squirrel monkey | DivE | 0 | 0 | 0 | 0 | 0 | 0 | 0.74 | 0.03 | 0.88 | 0.32 |
|  | dless | 0 | 0 | 0 | 0 | 0 | 0 | 0 | 0 | 0.94 | 0.03 |
| owl monkey - marmoset | DivE | 0 | 0 | 0 | 0 | 0.46 | 0.01 | 0.81 | 0.22 | 0.94 | 0.68 |
|  | dless | 0 | 0 | 0 | 0 | 0 | 0 | 0.88 | 0.01 | 0.97 | 0.21 |
| dusky titi - owl monkey | DivE | 0 | 0 | 0 | 0 | 0.67 | 0.07 | 0.9 | 0.48 | 0.96 | 0.84 |
|  | dless | 0 | 0 | 0 | 0 | 0 | 0 | 0.94 | 0.07 | 0.98 | 0.42 |
| human – dusky titi | DivE | 0 | 0 | 0.93 | 0.05 | 0.89 | 0.49 | 0.96 | 0.85 | 0.98 | 0.94 |
|  | dless | 0 | 0 | 0 | 0 | 0.96 | 0.09 | 0.98 | 0.55 | 0.99 | 0.85 |
| mouse lemur | DivE | 0 | 0 | 0 | 0 | 0 | 0 | 0.81 | 0.04 | 0.87 | 0.24 |
|  | dless | 0 | 0 | 0 | 0 | 0 | 0 | 0 | 0 | 0 | 0 |
| galago | DivE | 0 | 0 | 0 | 0 | 0.77 | 0.01 | 0.71 | 0.14 | 0.91 | 0.57 |
|  | dless | 0 | 0 | 0 | 0 | 0.77 | 0.01 | 0.71 | 0.14 | 0.91 | 0.57 |
| mouse lemur - galago | DivE | 0 | 0 | 0 | 0 | 0.85 | 0.08 | 0.92 | 0.56 | 0.96 | 0.87 |
|  | dless | 0 | 0 | 0 | 0 | 0 | 0 | 0.94 | 0.11 | 0.98 | 0.56 |

**Table S8.** Accuracy obtained by DivE and DLESS for the prediction of gained elements of different lengths in each clade^1^ of the phylogeny, when the selction strength is given by ρ=0.5.

| 0.01 loss | | **50 bp** | | **100 bp** | | **200 bp** | | **500 bp** | | **1000 bp** | |
| --- | --- | --- | --- | --- | --- | --- | --- | --- | --- | --- | --- |
| **tree** | **prog** | Pr | Re | Pr | Re | Pr | Re | Pr | Re | Pr | Re |
| human | DivE | 0.85 | 0.96 | 0.9 | 0.98 | 0.96 | 0.99 | 0.99 | 1 | 0.99 | 1 |
|  | dless | 0.8 | 0.92 | 0.86 | 0.88 | 0.89 | 0.9 | 0.92 | 0.95 | 0.95 | 0.97 |
| chimp | DivE | 0.8 | 0.94 | 0.9 | 0.96 | 0.94 | 0.98 | 0.99 | 1 | 0.99 | 1 |
|  | dless | 0.81 | 0.95 | 0.85 | 0.95 | 0.9 | 0.93 | 0.93 | 0.92 | 0.96 | 1 |
| human -chimp | DivE | 0.8 | 0.98 | 0.9 | 0.99 | 0.96 | 1 | 0.98 | 1 | 0.99 | 1 |
|  | dless | 0.72 | 0.9 | 0.82 | 0.95 | 0.88 | 0.95 | 0.95 | 1 | 0.98 | 0.96 |
| orangutan | DivE | 0.84 | 0.96 | 0.89 | 0.98 | 0.96 | 0.99 | 0.98 | 1 | 0.99 | 1 |
|  | dless | 0.8 | 0.92 | 0.87 | 0.96 | 0.9 | 0.93 | 0.96 | 0.96 | 0.98 | 1 |
| human -orangutan | DivE | 0.77 | 0.98 | 0.88 | 1 | 0.94 | 1 | 0.98 | 1 | 0.99 | 1 |
|  | dless | 0.68 | 0.93 | 0.79 | 0.96 | 0.87 | 0.98 | 0.96 | 1 | 0.99 | 1 |
| gibbon | DivE | 0.85 | 0.97 | 0.91 | 0.99 | 0.97 | 1 | 0.98 | 1 | 0.99 | 1 |
|  | dless | 0.82 | 0.89 | 0.87 | 0.89 | 0.92 | 0.94 | 0.96 | 0.98 | 0.99 | 0.99 |
| human - gibbon | DivE | 0.82 | 0.99 | 0.87 | 0.99 | 0.94 | 1 | 0.98 | 1 | 0.99 | 1 |
|  | dless | 0.66 | 0.79 | 0.78 | 0.97 | 0.91 | 0.99 | 0.98 | 1 | 0.99 | 1 |
| colobus monkey | DivE | 0.85 | 0.96 | 0.92 | 0.98 | 0.95 | 0.99 | 0.98 | 1 | 0.99 | 1 |
|  | dless | 0.8 | 0.89 | 0.87 | 0.92 | 0.91 | 0.95 | 0.95 | 0.99 | 0.98 | 1 |
| vervet | DivE | 0.79 | 0.95 | 0.91 | 0.97 | 0.96 | 0.99 | 0.98 | 1 | 0.99 | 1 |
|  | dless | 0.79 | 0.95 | 0.86 | 0.86 | 0.9 | 0.98 | 0.94 | 0.97 | 0.98 | 0.99 |
| baboon | DivE | 0.81 | 0.95 | 0.92 | 0.97 | 0.96 | 0.99 | 0.98 | 1 | 0.99 | 1 |
|  | dless | 0.8 | 0.92 | 0.86 | 0.94 | 0.9 | 0.91 | 0.94 | 0.95 | 0.97 | 0.99 |
| macaque | DivE | 0.81 | 0.94 | 0.92 | 0.97 | 0.96 | 0.99 | 0.98 | 1 | 0.99 | 1 |
|  | dless | 0.8 | 0.93 | 0.87 | 0.91 | 0.9 | 0.96 | 0.94 | 0.96 | 0.98 | 0.99 |
| baboon - macaque | DivE | 0.82 | 0.97 | 0.89 | 0.99 | 0.95 | 1 | 0.98 | 1 | 0.99 | 1 |
|  | dless | 0.76 | 0.93 | 0.8 | 0.93 | 0.87 | 0.97 | 0.95 | 0.99 | 0.98 | 0.99 |
| vervet - baboon | DivE | 0.78 | 0.98 | 0.87 | 0.99 | 0.93 | 1 | 0.98 | 1 | 0.99 | 1 |
|  | dless | 0.7 | 0.93 | 0.78 | 0.97 | 0.88 | 0.96 | 0.98 | 1 | 0.99 | 0.99 |
| colobus monkey - vervet | DivE | 0.78 | 0.98 | 0.88 | 1 | 0.95 | 1 | 0.99 | 1 | 0.99 | 1 |
|  | dless | 0.67 | 0.74 | 0.79 | 0.96 | 0.92 | 0.99 | 0.98 | 1 | 0.99 | 1 |
| human - colobus monkey | DivE | 0.7 | 0.99 | 0.86 | 1 | 0.93 | 1 | 0.98 | 1 | 0.99 | 1 |
|  | dless | 0.39 | 0.17 | 0.76 | 1 | 0.92 | 1 | 0.98 | 1 | 0.99 | 1 |
| dusky titi | DivE | 0.84 | 0.96 | 0.92 | 0.98 | 0.97 | 0.99 | 0.98 | 1 | 0.99 | 1 |
|  | dless | 0.8 | 0.9 | 0.87 | 0.96 | 0.91 | 0.99 | 0.97 | 0.98 | 0.99 | 0.99 |
| owl monkey | DivE | 0.82 | 0.97 | 0.91 | 0.98 | 0.96 | 0.99 | 0.99 | 1 | 0.99 | 1 |
|  | dless | 0.79 | 0.91 | 0.86 | 0.92 | 0.91 | 0.91 | 0.96 | 0.96 | 0.99 | 0.98 |
| marmoset | DivE | 0.83 | 0.96 | 0.92 | 0.98 | 0.96 | 0.99 | 0.99 | 1 | 0.99 | 1 |
|  | dless | 0.79 | 0.89 | 0.89 | 0.94 | 0.92 | 0.96 | 0.97 | 0.99 | 0.99 | 1 |
| squirrel monkey | DivE | 0.82 | 0.96 | 0.91 | 0.99 | 0.96 | 1 | 0.98 | 1 | 0.99 | 1 |
|  | dless | 0.81 | 0.92 | 0.88 | 0.98 | 0.92 | 0.95 | 0.97 | 0.97 | 0.99 | 1 |
| marmoset - squirrel monkey | DivE | 0.84 | 0.99 | 0.91 | 0.99 | 0.95 | 1 | 0.98 | 1 | 0.99 | 1 |
|  | dless | 0.75 | 0.86 | 0.85 | 0.95 | 0.91 | 0.96 | 0.98 | 1 | 0.99 | 1 |
| owl monkey - marmoset | DivE | 0.82 | 0.99 | 0.88 | 0.99 | 0.96 | 1 | 0.98 | 1 | 0.99 | 1 |
|  | dless | 0.71 | 0.67 | 0.81 | 0.98 | 0.93 | 0.99 | 0.98 | 1 | 0.99 | 0.99 |
| dusky titi - owl monkey | DivE | 0.79 | 0.99 | 0.92 | 1 | 0.96 | 1 | 0.98 | 1 | 0.99 | 1 |
|  | dless | 0.66 | 0.36 | 0.86 | 0.99 | 0.93 | 0.99 | 0.98 | 0.99 | 0.99 | 1 |
| human – dusky titi | DivE | 0.12 | 0.04 | 0.66 | 0.94 | 0.91 | 1 | 0.96 | 1 | 0.97 | 1 |
|  | dless | 0 | 0 | 0.24 | 0.02 | 0.89 | 1 | 0.92 | 1 | 0.97 | 0.98 |
| mouse lemur | DivE | 0.89 | 0.98 | 0.93 | 0.99 | 0.96 | 1 | 0.98 | 1 | 0.99 | 1 |
|  | dless | 0.81 | 0.72 | 0.89 | 0.97 | 0.94 | 0.99 | 0.98 | 1 | 0.99 | 1 |
| galago | DivE | 0.8 | 0.97 | 0.94 | 0.99 | 0.96 | 1 | 0.98 | 1 | 0.99 | 1 |
|  | dless | 0.78 | 0.68 | 0.9 | 0.96 | 0.95 | 0.98 | 0.98 | 1 | 0.99 | 0.99 |
| mouse lemur - galago | DivE | 0.81 | 0.94 | 0.91 | 0.99 | 0.94 | 1 | 0.99 | 1 | 0.99 | 1 |
|  | dless | 0.62 | 0.06 | 0.86 | 0.98 | 0.93 | 1 | 0.98 | 1 | 0.99 | 1 |

**Table S9.** Accuracy obtained by DivE and DLESS for the prediction of lost elements (the element in the subtree is evolving at the neutral rate, while outside the substree the element is constrained and is evolving more slowly) of different lengths in each clade^1^ of the phylogeny, when the selction strength is given by ρ=0.01.

| 0.02 loss | | **50 bp** | | **100 bp** | | **200 bp** | | **500 bp** | | **1000 bp** | |
| --- | --- | --- | --- | --- | --- | --- | --- | --- | --- | --- | --- |
| **tree** | **prog** | Pr | Re | Pr | Re | Pr | Re | Pr | Re | Pr | Re |
| human | DivE | 0.82 | 0.95 | 0.9 | 0.98 | 0.95 | 0.99 | 0.98 | 1 | 0.99 | 1 |
|  | dless | 0.81 | 0.84 | 0.84 | 0.93 | 0.89 | 0.93 | 0.92 | 0.95 | 0.95 | 0.96 |
| chimp | DivE | 0.83 | 0.94 | 0.9 | 0.96 | 0.95 | 0.98 | 0.99 | 1 | 0.99 | 1 |
|  | dless | 0.81 | 0.92 | 0.87 | 0.9 | 0.9 | 0.87 | 0.94 | 0.94 | 0.96 | 0.97 |
| human -chimp | DivE | 0.82 | 0.97 | 0.9 | 0.99 | 0.94 | 1 | 0.98 | 1 | 0.99 | 1 |
|  | dless | 0.75 | 0.91 | 0.81 | 0.96 | 0.87 | 0.95 | 0.94 | 0.98 | 0.99 | 1 |
| orangutan | DivE | 0.82 | 0.95 | 0.91 | 0.98 | 0.96 | 0.99 | 0.98 | 1 | 0.99 | 1 |
|  | dless | 0.77 | 0.89 | 0.86 | 0.85 | 0.91 | 0.95 | 0.95 | 0.99 | 0.98 | 0.99 |
| human -orangutan | DivE | 0.79 | 0.98 | 0.89 | 0.99 | 0.95 | 1 | 0.98 | 1 | 0.99 | 1 |
|  | dless | 0.7 | 0.87 | 0.79 | 0.97 | 0.89 | 0.97 | 0.97 | 1 | 0.99 | 0.99 |
| gibbon | DivE | 0.84 | 0.96 | 0.92 | 0.98 | 0.96 | 0.99 | 0.99 | 1 | 0.99 | 1 |
|  | dless | 0.82 | 0.9 | 0.87 | 0.92 | 0.91 | 0.97 | 0.97 | 0.97 | 0.99 | 1 |
| human - gibbon | DivE | 0.74 | 0.99 | 0.87 | 0.99 | 0.94 | 1 | 0.98 | 1 | 0.99 | 1 |
|  | dless | 0.63 | 0.77 | 0.81 | 0.99 | 0.9 | 1 | 0.98 | 1 | 0.99 | 0.99 |
| colobus monkey | DivE | 0.85 | 0.95 | 0.94 | 0.98 | 0.96 | 0.98 | 0.98 | 1 | 0.99 | 1 |
|  | dless | 0.81 | 0.92 | 0.88 | 0.96 | 0.91 | 0.9 | 0.96 | 1 | 0.98 | 0.99 |
| vervet | DivE | 0.8 | 0.94 | 0.89 | 0.98 | 0.95 | 0.99 | 0.98 | 1 | 0.99 | 1 |
|  | dless | 0.8 | 0.9 | 0.85 | 0.9 | 0.9 | 0.92 | 0.95 | 0.97 | 0.97 | 0.98 |
| baboon | DivE | 0.86 | 0.95 | 0.9 | 0.96 | 0.95 | 0.99 | 0.98 | 1 | 0.99 | 1 |
|  | dless | 0.81 | 0.91 | 0.86 | 0.95 | 0.89 | 0.94 | 0.94 | 0.94 | 0.97 | 0.98 |
| macaque | DivE | 0.84 | 0.94 | 0.87 | 0.97 | 0.96 | 0.99 | 0.98 | 1 | 0.99 | 1 |
|  | dless | 0.79 | 0.9 | 0.84 | 0.9 | 0.9 | 0.91 | 0.94 | 0.96 | 0.97 | 0.99 |
| baboon - macaque | DivE | 0.77 | 0.97 | 0.9 | 0.99 | 0.94 | 1 | 0.98 | 1 | 0.99 | 1 |
|  | dless | 0.75 | 0.93 | 0.81 | 0.95 | 0.88 | 0.98 | 0.94 | 0.98 | 0.98 | 1 |
| vervet - baboon | DivE | 0.8 | 0.98 | 0.89 | 0.99 | 0.94 | 1 | 0.98 | 1 | 0.99 | 1 |
|  | dless | 0.69 | 0.91 | 0.8 | 0.96 | 0.87 | 1 | 0.96 | 1 | 0.99 | 0.98 |
| colobus monkey - vervet | DivE | 0.79 | 0.98 | 0.9 | 1 | 0.95 | 1 | 0.98 | 1 | 0.99 | 1 |
|  | dless | 0.64 | 0.68 | 0.81 | 0.98 | 0.92 | 0.99 | 0.97 | 1 | 0.99 | 1 |
| human - colobus monkey | DivE | 0.72 | 0.99 | 0.89 | 0.99 | 0.94 | 1 | 0.98 | 1 | 0.99 | 1 |
|  | dless | 0.44 | 0.13 | 0.81 | 1 | 0.93 | 1 | 0.98 | 0.99 | 0.99 | 1 |
| dusky titi | DivE | 0.81 | 0.97 | 0.91 | 0.98 | 0.96 | 1 | 0.98 | 1 | 0.99 | 1 |
|  | dless | 0.8 | 0.95 | 0.87 | 0.99 | 0.92 | 0.95 | 0.97 | 0.99 | 0.99 | 1 |
| owl monkey | DivE | 0.82 | 0.95 | 0.9 | 0.98 | 0.97 | 1 | 0.99 | 1 | 0.99 | 1 |
|  | dless | 0.79 | 0.93 | 0.88 | 0.92 | 0.92 | 0.97 | 0.97 | 0.98 | 0.99 | 0.99 |
| marmoset | DivE | 0.81 | 0.96 | 0.91 | 0.98 | 0.96 | 0.99 | 0.99 | 1 | 0.99 | 1 |
|  | dless | 0.78 | 0.88 | 0.87 | 0.95 | 0.91 | 0.97 | 0.97 | 0.97 | 0.99 | 0.99 |
| squirrel monkey | DivE | 0.83 | 0.96 | 0.9 | 0.98 | 0.96 | 0.99 | 0.98 | 1 | 0.99 | 1 |
|  | dless | 0.81 | 0.92 | 0.86 | 0.91 | 0.91 | 0.95 | 0.97 | 0.99 | 0.99 | 1 |
| marmoset - squirrel monkey | DivE | 0.85 | 0.98 | 0.89 | 0.99 | 0.97 | 1 | 0.98 | 1 | 0.99 | 1 |
|  | dless | 0.77 | 0.78 | 0.84 | 0.97 | 0.93 | 0.99 | 0.98 | 1 | 0.99 | 1 |
| owl monkey - marmoset | DivE | 0.99 | 0.88 | 1 | 0.96 | 1 | 0.98 | 1 | 0.99 | 1 |  |
|  | dless | 0.68 | 0.68 | 0.83 | 0.98 | 0.92 | 0.99 | 0.98 | 1 | 0.99 | 0.98 |
| dusky titi - owl monkey | DivE | 0.81 | 0.98 | 0.9 | 1 | 0.96 | 1 | 0.98 | 1 | 0.99 | 1 |
|  | dless | 0.61 | 0.31 | 0.85 | 0.99 | 0.93 | 0.99 | 0.97 | 1 | 0.99 | 1 |
| human – dusky titi | DivE | 0.1 | 0.02 | 0.64 | 0.94 | 0.88 | 1 | 0.96 | 1 | 0.98 | 1 |
|  | dless | 0 | 0 | 0.24 | 0.04 | 0.87 | 1 | 0.95 | 1 | 0.97 | 0.99 |
| mouse lemur | DivE | 0.82 | 0.98 | 0.93 | 0.99 | 0.96 | 1 | 0.98 | 1 | 0.99 | 1 |
|  | dless | 0.8 | 0.69 | 0.89 | 0.94 | 0.94 | 0.99 | 0.98 | 1 | 0.99 | 0.99 |
| galago | DivE | 0.83 | 0.98 | 0.92 | 0.99 | 0.95 | 1 | 0.99 | 1 | 0.99 | 1 |
|  | dless | 0.78 | 0.64 | 0.89 | 0.99 | 0.95 | 0.98 | 0.98 | 1 | 0.99 | 1 |
| mouse lemur - galago | DivE | 0.82 | 0.97 | 0.91 | 0.99 | 0.95 | 1 | 0.98 | 1 | 0.99 | 1 |
|  | dless | 0.67 | 0.08 | 0.87 | 0.99 | 0.92 | 1 | 0.98 | 1 | 0.99 | 0.98 |

**Table S10.** Accuracy obtained by DivE and DLESS for the prediction of lost elements of different lengths in each clade^1^ of the phylogeny, when the selction strength is given b ρ=0.02.

| 0.05 loss | | **50 bp** | | **100 bp** | | **200 bp** | | **500 bp** | | **1000 bp** | |
| --- | --- | --- | --- | --- | --- | --- | --- | --- | --- | --- | --- |
| **tree** | **prog** | Pr | Re | Pr | Re | Pr | Re | Pr | Re | Pr | Re |
| human | DivE | 0.82 | 0.94 | 0.9 | 0.97 | 0.95 | 0.99 | 0.99 | 1 | 0.99 | 1 |
|  | dless | 0.81 | 0.85 | 0.87 | 0.91 | 0.89 | 0.9 | 0.93 | 0.93 | 0.94 | 0.94 |
| chimp | DivE | 0.83 | 0.93 | 0.91 | 0.96 | 0.94 | 0.98 | 0.99 | 1 | 0.99 | 1 |
|  | dless | 0.79 | 0.92 | 0.86 | 0.91 | 0.89 | 0.97 | 0.94 | 0.93 | 0.95 | 0.95 |
| human -chimp | DivE | 0.78 | 0.95 | 0.89 | 0.98 | 0.95 | 0.99 | 0.98 | 1 | 0.99 | 1 |
|  | dless | 0.74 | 0.89 | 0.82 | 0.94 | 0.88 | 0.95 | 0.94 | 0.99 | 0.98 | 0.99 |
| orangutan | DivE | 0.8 | 0.93 | 0.91 | 0.97 | 0.96 | 0.99 | 0.99 | 1 | 0.99 | 1 |
|  | dless | 0.78 | 0.87 | 0.87 | 0.9 | 0.91 | 0.96 | 0.96 | 0.98 | 0.99 | 0.99 |
| human -orangutan | DivE | 0.78 | 0.97 | 0.86 | 0.99 | 0.93 | 1 | 0.98 | 1 | 0.99 | 1 |
|  | dless | 0.66 | 0.85 | 0.77 | 0.97 | 0.88 | 1 | 0.97 | 0.99 | 0.99 | 1 |
| gibbon | DivE | 0.82 | 0.94 | 0.88 | 0.98 | 0.96 | 0.99 | 0.99 | 1 | 0.99 | 1 |
|  | dless | 0.78 | 0.92 | 0.86 | 0.92 | 0.91 | 0.97 | 0.97 | 0.97 | 0.99 | 1 |
| human - gibbon | DivE | 0.79 | 0.98 | 0.9 | 0.99 | 0.95 | 1 | 0.98 | 1 | 0.99 | 1 |
|  | dless | 0.59 | 0.54 | 0.82 | 0.98 | 0.92 | 1 | 0.97 | 1 | 0.99 | 0.99 |
| colobus monkey | DivE | 0.78 | 0.95 | 0.91 | 0.98 | 0.96 | 0.99 | 0.98 | 1 | 0.99 | 1 |
|  | dless | 0.8 | 0.91 | 0.88 | 0.92 | 0.91 | 0.97 | 0.96 | 0.98 | 0.99 | 1 |
| vervet | DivE | 0.79 | 0.94 | 0.92 | 0.97 | 0.95 | 0.99 | 0.99 | 1 | 0.99 | 1 |
|  | dless | 0.8 | 0.88 | 0.85 | 0.89 | 0.9 | 0.94 | 0.94 | 0.94 | 0.97 | 0.97 |
| baboon | DivE | 0.81 | 0.93 | 0.87 | 0.95 | 0.97 | 0.99 | 0.98 | 1 | 0.99 | 1 |
|  | dless | 0.79 | 0.9 | 0.84 | 0.95 | 0.91 | 0.86 | 0.94 | 0.94 | 0.97 | 1 |
| macaque | DivE | 0.81 | 0.93 | 0.92 | 0.96 | 0.96 | 0.98 | 0.98 | 0.99 | 0.99 | 1 |
|  | dless | 0.79 | 0.89 | 0.87 | 0.96 | 0.9 | 0.95 | 0.94 | 0.95 | 0.97 | 1 |
| baboon - macaque | DivE | 0.78 | 0.96 | 0.91 | 0.98 | 0.94 | 1 | 0.98 | 1 | 0.99 | 1 |
|  | dless | 0.72 | 0.89 | 0.83 | 0.95 | 0.87 | 0.96 | 0.95 | 1 | 0.98 | 0.99 |
| vervet - baboon | DivE | 0.78 | 0.97 | 0.89 | 0.99 | 0.93 | 1 | 0.98 | 1 | 0.99 | 1 |
|  | dless | 0.69 | 0.85 | 0.78 | 0.98 | 0.88 | 0.98 | 0.97 | 1 | 0.99 | 0.99 |
| colobus monkey - vervet | DivE | 0.75 | 0.98 | 0.89 | 0.99 | 0.96 | 1 | 0.99 | 1 | 0.99 | 1 |
|  | dless | 0.63 | 0.62 | 0.8 | 0.99 | 0.92 | 0.99 | 0.98 | 1 | 0.99 | 0.99 |
| human - colobus monkey | DivE | 0.7 | 0.94 | 0.83 | 0.99 | 0.95 | 1 | 0.98 | 1 | 0.99 | 1 |
|  | dless | 0.42 | 0.09 | 0.74 | 0.98 | 0.9 | 1 | 0.98 | 1 | 0.99 | 1 |
| dusky titi | DivE | 0.86 | 0.95 | 0.92 | 0.98 | 0.96 | 0.99 | 0.98 | 1 | 0.99 | 1 |
|  | dless | 0.81 | 0.8 | 0.87 | 0.91 | 0.92 | 0.96 | 0.97 | 1 | 0.99 | 1 |
| owl monkey | DivE | 0.79 | 0.95 | 0.91 | 0.97 | 0.95 | 0.99 | 0.98 | 1 | 0.99 | 1 |
|  | dless | 0.8 | 0.89 | 0.86 | 0.9 | 0.91 | 0.93 | 0.97 | 0.99 | 0.99 | 1 |
| marmoset | DivE | 0.8 | 0.94 | 0.92 | 0.98 | 0.96 | 0.99 | 0.99 | 1 | 0.99 | 1 |
|  | dless | 0.82 | 0.82 | 0.88 | 0.93 | 0.91 | 0.95 | 0.97 | 1 | 0.99 | 0.98 |
| squirrel monkey | DivE | 0.8 | 0.95 | 0.92 | 0.98 | 0.96 | 0.99 | 0.98 | 1 | 0.99 | 1 |
|  | dless | 0.78 | 0.81 | 0.88 | 0.9 | 0.92 | 0.94 | 0.97 | 0.99 | 0.99 | 1 |
| marmoset - squirrel monkey | DivE | 0.83 | 0.96 | 0.9 | 0.99 | 0.96 | 1 | 0.98 | 1 | 0.99 | 1 |
|  | dless | 0.73 | 0.79 | 0.84 | 0.96 | 0.93 | 0.99 | 0.97 | 1 | 0.99 | 0.99 |
| owl monkey - marmoset | DivE | 0.78 | 0.97 | 0.9 | 0.99 | 0.96 | 1 | 0.98 | 1 | 0.99 | 1 |
|  | dless | 0.67 | 0.53 | 0.84 | 0.96 | 0.93 | 0.97 | 0.98 | 1 | 0.99 | 1 |
| dusky titi - owl monkey | DivE | 0.79 | 0.97 | 0.89 | 0.99 | 0.94 | 1 | 0.98 | 1 | 0.99 | 1 |
|  | dless | 0.62 | 0.14 | 0.85 | 0.98 | 0.93 | 1 | 0.98 | 1 | 0.99 | 1 |
| human – dusky titi | DivE | 0.2 | 0.05 | 0.7 | 0.87 | 0.9 | 0.99 | 0.96 | 1 | 0.98 | 1 |
|  | dless | 0 | 0 | 0.45 | 0.02 | 0.88 | 0.98 | 0.95 | 1 | 0.97 | 1 |
| mouse lemur | DivE | 0.81 | 0.97 | 0.94 | 0.98 | 0.96 | 1 | 0.98 | 1 | 0.99 | 1 |
|  | dless | 0.77 | 0.68 | 0.89 | 0.97 | 0.95 | 0.99 | 0.98 | 1 | 0.99 | 1 |
| galago | DivE | 0.86 | 0.98 | 0.94 | 0.99 | 0.97 | 1 | 0.98 | 1 | 0.99 | 1 |
|  | dless | 0.77 | 0.47 | 0.9 | 0.95 | 0.95 | 1 | 0.98 | 1 | 0.99 | 1 |
| mouse lemur - galago | DivE | 0.76 | 0.81 | 0.91 | 0.99 | 0.95 | 1 | 0.98 | 1 | 0.99 | 1 |
|  | dless | 0.62 | 0.06 | 0.86 | 0.96 | 0.93 | 0.97 | 0.98 | 1 | 0.99 | 1 |

**Table S11.** Accuracy obtained by DivE and DLESS for the prediction of lost elements of different lengths in each clade^1^ of the phylogeny, when the selction strength is given by ρ=0.05.

| 0.1 loss | | **50 bp** | | **100 bp** | | **200 bp** | | **500 bp** | | **1000 bp** | |
| --- | --- | --- | --- | --- | --- | --- | --- | --- | --- | --- | --- |
| **tree** | **prog** | Pr | Re | Pr | Re | Pr | Re | Pr | Re | Pr | Re |
| human | DivE | 0.82 | 0.92 | 0.9 | 0.95 | 0.94 | 0.97 | 0.98 | 0.99 | 0.99 | 1 |
|  | dless | 0.8 | 0.8 | 0.87 | 0.87 | 0.9 | 0.9 | 0.92 | 0.93 | 0.95 | 1 |
| chimp | DivE | 0.75 | 0.91 | 0.9 | 0.94 | 0.94 | 0.97 | 0.98 | 0.99 | 0.99 | 1 |
|  | dless | 0.76 | 0.79 | 0.88 | 0.91 | 0.9 | 0.94 | 0.95 | 0.98 | 0.96 | 0.97 |
| human -chimp | DivE | 0.8 | 0.92 | 0.86 | 0.96 | 0.95 | 0.99 | 0.98 | 1 | 0.99 | 1 |
|  | dless | 0.74 | 0.77 | 0.8 | 0.97 | 0.87 | 0.92 | 0.94 | 0.96 | 0.99 | 1 |
| orangutan | DivE | 0.77 | 0.93 | 0.91 | 0.96 | 0.94 | 0.98 | 0.98 | 0.99 | 0.99 | 1 |
|  | dless | 0.79 | 0.74 | 0.86 | 0.91 | 0.9 | 0.93 | 0.96 | 0.97 | 0.98 | 0.98 |
| human -orangutan | DivE | 0.74 | 0.95 | 0.88 | 0.97 | 0.95 | 0.99 | 0.98 | 1 | 0.99 | 1 |
|  | dless | 0.68 | 0.65 | 0.8 | 0.97 | 0.89 | 0.98 | 0.96 | 0.99 | 0.99 | 1 |
| gibbon | DivE | 0.82 | 0.91 | 0.91 | 0.96 | 0.96 | 0.99 | 0.99 | 1 | 0.99 | 1 |
|  | dless | 0.78 | 0.69 | 0.87 | 0.93 | 0.92 | 0.95 | 0.96 | 0.99 | 0.99 | 1 |
| human - gibbon | DivE | 0.74 | 0.96 | 0.88 | 0.99 | 0.92 | 0.99 | 0.98 | 1 | 0.99 | 1 |
|  | dless | 0.6 | 0.47 | 0.8 | 0.96 | 0.89 | 0.98 | 0.98 | 1 | 0.99 | 1 |
| colobus monkey | DivE | 0.84 | 0.93 | 0.9 | 0.97 | 0.95 | 0.98 | 0.98 | 1 | 0.99 | 1 |
|  | dless | 0.81 | 0.77 | 0.86 | 0.92 | 0.9 | 0.95 | 0.96 | 0.97 | 0.98 | 0.98 |
| vervet | DivE | 0.79 | 0.92 | 0.9 | 0.96 | 0.96 | 0.98 | 0.98 | 1 | 0.99 | 1 |
|  | dless | 0.78 | 0.83 | 0.85 | 0.92 | 0.92 | 0.92 | 0.95 | 0.96 | 0.98 | 0.99 |
| baboon | DivE | 0.83 | 0.9 | 0.9 | 0.95 | 0.95 | 0.98 | 0.98 | 0.99 | 0.99 | 1 |
|  | dless | 0.8 | 0.81 | 0.85 | 0.88 | 0.91 | 0.87 | 0.94 | 0.94 | 0.97 | 1 |
| macaque | DivE | 0.8 | 0.91 | 0.92 | 0.95 | 0.95 | 0.98 | 0.99 | 1 | 0.99 | 1 |
|  | dless | 0.79 | 0.79 | 0.88 | 0.95 | 0.9 | 0.98 | 0.95 | 0.99 | 0.97 | 1 |
| baboon - macaque | DivE | 0.8 | 0.94 | 0.86 | 0.96 | 0.95 | 0.99 | 0.98 | 1 | 0.99 | 1 |
|  | dless | 0.72 | 0.65 | 0.8 | 0.91 | 0.89 | 0.97 | 0.95 | 1 | 0.99 | 1 |
| vervet - baboon | DivE | 0.75 | 0.95 | 0.86 | 0.98 | 0.94 | 0.99 | 0.98 | 1 | 0.99 | 1 |
|  | dless | 0.69 | 0.67 | 0.78 | 0.95 | 0.88 | 0.96 | 0.97 | 1 | 0.99 | 1 |
| colobus monkey - vervet | DivE | 0.79 | 0.96 | 0.89 | 0.99 | 0.93 | 0.99 | 0.98 | 1 | 0.99 | 1 |
|  | dless | 0.64 | 0.4 | 0.78 | 0.98 | 0.91 | 1 | 0.98 | 1 | 0.99 | 1 |
| human - colobus monkey | DivE | 0.64 | 0.84 | 0.82 | 0.99 | 0.95 | 0.99 | 0.98 | 1 | 0.99 | 1 |
|  | dless | 0.35 | 0.1 | 0.78 | 0.96 | 0.94 | 0.98 | 0.98 | 1 | 0.99 | 1 |
| dusky titi | DivE | 0.84 | 0.93 | 0.92 | 0.97 | 0.96 | 0.98 | 0.98 | 1 | 0.99 | 1 |
|  | dless | 0.82 | 0.71 | 0.87 | 0.91 | 0.92 | 0.96 | 0.97 | 0.99 | 0.99 | 0.99 |
| owl monkey | DivE | 0.79 | 0.93 | 0.91 | 0.97 | 0.97 | 0.98 | 0.99 | 1 | 0.99 | 1 |
|  | dless | 0.81 | 0.67 | 0.87 | 0.94 | 0.91 | 0.95 | 0.96 | 0.99 | 0.99 | 1 |
| marmoset | DivE | 0.83 | 0.93 | 0.9 | 0.96 | 0.97 | 0.99 | 0.98 | 1 | 0.99 | 1 |
|  | dless | 0.8 | 0.57 | 0.88 | 0.88 | 0.92 | 0.99 | 0.97 | 1 | 0.99 | 1 |
| squirrel monkey | DivE | 0.81 | 0.92 | 0.92 | 0.96 | 0.96 | 0.99 | 0.99 | 1 | 0.99 | 1 |
|  | dless | 0.76 | 0.64 | 0.87 | 0.93 | 0.92 | 0.97 | 0.97 | 1 | 0.99 | 1 |
| marmoset - squirrel monkey | DivE | 0.81 | 0.94 | 0.92 | 0.98 | 0.94 | 0.99 | 0.99 | 1 | 0.99 | 1 |
|  | dless | 0.67 | 0.51 | 0.86 | 0.94 | 0.91 | 0.97 | 0.97 | 1 | 0.99 | 0.99 |
| owl monkey - marmoset | DivE | 0.76 | 0.93 | 0.9 | 0.98 | 0.94 | 0.99 | 0.98 | 1 | 0.99 | 1 |
|  | dless | 0.68 | 0.38 | 0.84 | 0.99 | 0.92 | 1 | 0.97 | 1 | 0.99 | 0.99 |
| dusky titi - owl monkey | DivE | 0.79 | 0.86 | 0.91 | 0.98 | 0.97 | 0.99 | 0.98 | 1 | 0.99 | 1 |
|  | dless | 0.35 | 0.08 | 0.89 | 0.97 | 0.94 | 1 | 0.98 | 1 | 0.99 | 1 |
| human – dusky titi | DivE | 0.06 | 0.02 | 0.53 | 0.62 | 0.86 | 0.99 | 0.96 | 0.99 | 0.98 | 1 |
|  | dless | 0 | 0 | 0.24 | 0.02 | 0.87 | 0.82 | 0.95 | 1 | 0.97 | 1 |
| mouse lemur | DivE | 0.83 | 0.93 | 0.92 | 0.98 | 0.96 | 0.99 | 0.99 | 1 | 0.99 | 1 |
|  | dless | 0.77 | 0.39 | 0.9 | 0.96 | 0.94 | 0.97 | 0.98 | 1 | 0.99 | 1 |
| galago | DivE | 0.86 | 0.93 | 0.93 | 0.98 | 0.96 | 0.99 | 0.99 | 1 | 0.99 | 1 |
|  | dless | 0.78 | 0.26 | 0.9 | 0.96 | 0.94 | 0.99 | 0.98 | 1 | 0.99 | 0.99 |
| mouse lemur - galago | DivE | 0.75 | 0.77 | 0.89 | 0.98 | 0.94 | 0.99 | 0.98 | 1 | 0.99 | 1 |
|  | dless | 0.62 | 0.05 | 0.87 | 0.89 | 0.92 | 1 | 0.97 | 1 | 0.99 | 1 |

**Table S12.** Accuracy obtained by DivE and DLESS for the prediction of lost elements of different lengths in each clade^1^ of the phylogeny, when the selction strength is given by ρ=0.1.

| 0.2 loss | | **50 bp** | | **100 bp** | | **200 bp** | | **500 bp** | | **1000 bp** | |
| --- | --- | --- | --- | --- | --- | --- | --- | --- | --- | --- | --- |
| **tree** | **prog** | Pr | Re | Pr | Re | Pr | Re | Pr | Re | Pr | Re |
| human | DivE | 0.77 | 0.81 | 0.89 | 0.9 | 0.93 | 0.93 | 0.96 | 0.97 | 0.99 | 0.99 |
|  | dless | 0.79 | 0.29 | 0.87 | 0.95 | 0.9 | 0.9 | 0.92 | 0.92 | 0.95 | 0.99 |
| chimp | DivE | 0.75 | 0.8 | 0.86 | 0.9 | 0.93 | 0.94 | 0.97 | 0.98 | 0.98 | 0.99 |
|  | dless | 0.78 | 0.39 | 0.87 | 0.94 | 0.9 | 0.93 | 0.93 | 0.92 | 0.96 | 0.99 |
| human -chimp | DivE | 0.73 | 0.79 | 0.86 | 0.92 | 0.95 | 0.95 | 0.97 | 0.99 | 0.99 | 1 |
|  | dless | 0.65 | 0.26 | 0.82 | 0.94 | 0.89 | 0.94 | 0.93 | 0.99 | 0.98 | 1 |
| orangutan | DivE | 0.79 | 0.73 | 0.89 | 0.9 | 0.94 | 0.95 | 0.97 | 0.97 | 0.99 | 1 |
|  | dless | 0.74 | 0.32 | 0.88 | 0.93 | 0.92 | 0.92 | 0.96 | 0.96 | 0.98 | 0.99 |
| human -orangutan | DivE | 0.73 | 0.71 | 0.84 | 0.94 | 0.91 | 0.97 | 0.97 | 0.99 | 0.99 | 1 |
|  | dless | 0.68 | 0.26 | 0.79 | 0.92 | 0.86 | 0.97 | 0.97 | 1 | 0.99 | 1 |
| gibbon | DivE | 0.78 | 0.81 | 0.89 | 0.91 | 0.95 | 0.95 | 0.98 | 0.99 | 0.99 | 1 |
|  | dless | 0.73 | 0.36 | 0.88 | 0.96 | 0.92 | 0.95 | 0.96 | 0.99 | 0.99 | 1 |
| human - gibbon | DivE | 0.71 | 0.64 | 0.84 | 0.95 | 0.9 | 0.98 | 0.98 | 0.99 | 0.99 | 1 |
|  | dless | 0.55 | 0.16 | 0.79 | 0.92 | 0.89 | 0.97 | 0.98 | 1 | 0.99 | 0.99 |
| colobus monkey | DivE | 0.77 | 0.82 | 0.89 | 0.91 | 0.94 | 0.95 | 0.98 | 0.99 | 0.99 | 0.99 |
|  | dless | 0.73 | 0.34 | 0.87 | 0.96 | 0.91 | 0.93 | 0.96 | 0.98 | 0.99 | 1 |
| vervet | DivE | 0.8 | 0.78 | 0.89 | 0.91 | 0.93 | 0.95 | 0.98 | 0.99 | 0.99 | 1 |
|  | dless | 0.77 | 0.36 | 0.88 | 0.93 | 0.91 | 0.92 | 0.95 | 1 | 0.97 | 0.97 |
| baboon | DivE | 0.83 | 0.8 | 0.89 | 0.91 | 0.93 | 0.94 | 0.97 | 0.97 | 0.99 | 0.99 |
|  | dless | 0.81 | 0.38 | 0.88 | 0.92 | 0.9 | 0.93 | 0.94 | 0.97 | 0.98 | 0.97 |
| macaque | DivE | 0.8 | 0.82 | 0.86 | 0.9 | 0.93 | 0.93 | 0.97 | 0.97 | 0.99 | 0.99 |
|  | dless | 0.72 | 0.32 | 0.87 | 0.92 | 0.91 | 0.94 | 0.95 | 0.97 | 0.98 | 0.99 |
| baboon - macaque | DivE | 0.78 | 0.79 | 0.85 | 0.93 | 0.91 | 0.96 | 0.97 | 0.98 | 0.99 | 1 |
|  | dless | 0.76 | 0.26 | 0.82 | 0.93 | 0.87 | 0.95 | 0.94 | 0.99 | 0.98 | 0.98 |
| vervet - baboon | DivE | 0.74 | 0.73 | 0.81 | 0.94 | 0.9 | 0.96 | 0.97 | 0.99 | 0.99 | 1 |
|  | dless | 0.62 | 0.24 | 0.8 | 0.96 | 0.88 | 0.98 | 0.97 | 0.99 | 0.99 | 0.99 |
| colobus monkey - vervet | DivE | 0.7 | 0.6 | 0.83 | 0.95 | 0.93 | 0.98 | 0.98 | 0.99 | 0.99 | 1 |
|  | dless | 0.6 | 0.13 | 0.79 | 0.92 | 0.92 | 0.99 | 0.98 | 1 | 0.99 | 0.99 |
| human - colobus monkey | DivE | 0.61 | 0.47 | 0.75 | 0.96 | 0.91 | 0.98 | 0.98 | 0.99 | 0.99 | 1 |
|  | dless | 0.15 | 0.02 | 0.72 | 0.72 | 0.91 | 0.97 | 0.98 | 1 | 0.99 | 0.99 |
| dusky titi | DivE | 0.82 | 0.76 | 0.88 | 0.92 | 0.94 | 0.96 | 0.98 | 0.99 | 0.99 | 1 |
|  | dless | 0.72 | 0.28 | 0.88 | 0.95 | 0.93 | 0.96 | 0.97 | 1 | 0.99 | 1 |
| owl monkey | DivE | 0.81 | 0.78 | 0.89 | 0.93 | 0.94 | 0.96 | 0.98 | 0.99 | 0.99 | 1 |
|  | dless | 0.78 | 0.32 | 0.88 | 0.94 | 0.92 | 0.96 | 0.97 | 0.99 | 0.99 | 0.98 |
| marmoset | DivE | 0.79 | 0.74 | 0.88 | 0.92 | 0.96 | 0.96 | 0.98 | 0.99 | 0.99 | 1 |
|  | dless | 0.79 | 0.33 | 0.88 | 0.93 | 0.93 | 0.98 | 0.97 | 0.97 | 0.99 | 0.99 |
| squirrel monkey | DivE | 0.78 | 0.83 | 0.92 | 0.93 | 0.95 | 0.96 | 0.98 | 0.99 | 0.99 | 1 |
|  | dless | 0.71 | 0.28 | 0.88 | 0.91 | 0.93 | 0.95 | 0.97 | 0.99 | 0.99 | 1 |
| marmoset - squirrel monkey | DivE | 0.75 | 0.8 | 0.88 | 0.94 | 0.93 | 0.97 | 0.98 | 0.99 | 0.99 | 1 |
|  | dless | 0.71 | 0.19 | 0.84 | 0.96 | 0.92 | 0.97 | 0.98 | 1 | 0.99 | 0.99 |
| owl monkey - marmoset | DivE | 0.7 | 0.66 | 0.86 | 0.96 | 0.94 | 0.97 | 0.98 | 0.99 | 0.99 | 1 |
|  | dless | 0.53 | 0.11 | 0.83 | 0.9 | 0.93 | 0.98 | 0.98 | 1 | 0.99 | 1 |
| dusky titi - owl monkey | DivE | 0.71 | 0.51 | 0.88 | 0.96 | 0.94 | 0.98 | 0.98 | 0.99 | 0.99 | 0.99 |
|  | dless | 0.42 | 0.04 | 0.86 | 0.82 | 0.95 | 0.99 | 0.98 | 1 | 0.99 | 0.99 |
| human – dusky titi | DivE | 0.1 | 0.02 | 0.48 | 0.17 | 0.76 | 0.81 | 0.94 | 0.98 | 0.97 | 0.99 |
|  | dless | 0 | 0 | 0 | 0 | 0.73 | 0.33 | 0.95 | 0.99 | 0.97 | 0.99 |
| mouse lemur | DivE | 0.81 | 0.6 | 0.86 | 0.94 | 0.96 | 0.97 | 0.98 | 0.99 | 0.99 | 1 |
|  | dless | 0.82 | 0.16 | 0.89 | 0.93 | 0.94 | 0.98 | 0.98 | 1 | 0.99 | 1 |
| galago | DivE | 0.74 | 0.73 | 0.9 | 0.95 | 0.96 | 0.98 | 0.98 | 0.99 | 0.99 | 1 |
|  | dless | 0.59 | 0.1 | 0.9 | 0.9 | 0.95 | 0.96 | 0.98 | 1 | 0.99 | 1 |
| mouse lemur - galago | DivE | 0.64 | 0.26 | 0.88 | 0.89 | 0.93 | 0.97 | 0.98 | 0.99 | 0.99 | 0.99 |
|  | dless | 0.75 | 0.01 | 0.85 | 0.47 | 0.94 | 0.98 | 0.98 | 1 | 0.99 | 1 |

**Table S13.** Accuracy obtained by DivE and DLESS for the prediction of lost elements of different lengths in each clade^1^ of the phylogeny, when the selction strength is given by ρ=0.2.

| 0.3 loss | | **50 bp** | | **100 bp** | | **200 bp** | | **500 bp** | | **1000 bp** | |
| --- | --- | --- | --- | --- | --- | --- | --- | --- | --- | --- | --- |
| **tree** | **prog** | Pr | Re | Pr | Re | Pr | Re | Pr | Re | Pr | Re |
| human | DivE | 0.72 | 0.55 | 0.83 | 0.86 | 0.91 | 0.89 | 0.95 | 0.94 | 0.97 | 0.97 |
|  | dless | 0.76 | 0.16 | 0.88 | 0.84 | 0.91 | 0.93 | 0.93 | 0.97 | 0.94 | 0.95 |
| chimp | DivE | 0.8 | 0.51 | 0.84 | 0.82 | 0.91 | 0.9 | 0.95 | 0.94 | 0.97 | 0.96 |
|  | dless | 0.68 | 0.14 | 0.85 | 0.81 | 0.9 | 0.93 | 0.94 | 0.93 | 0.96 | 0.98 |
| human -chimp | DivE | 0.71 | 0.48 | 0.81 | 0.88 | 0.9 | 0.92 | 0.95 | 0.97 | 0.98 | 0.99 |
|  | dless | 0.67 | 0.13 | 0.81 | 0.78 | 0.87 | 0.92 | 0.95 | 0.97 | 0.99 | 0.99 |
| orangutan | DivE | 0.74 | 0.46 | 0.87 | 0.83 | 0.92 | 0.9 | 0.96 | 0.95 | 0.98 | 0.98 |
|  | dless | 0.79 | 0.13 | 0.88 | 0.85 | 0.92 | 0.95 | 0.96 | 0.98 | 0.99 | 0.97 |
| human -orangutan | DivE | 0.69 | 0.46 | 0.83 | 0.88 | 0.87 | 0.95 | 0.96 | 0.98 | 0.97 | 0.99 |
|  | dless | 0.67 | 0.05 | 0.8 | 0.68 | 0.87 | 0.96 | 0.97 | 0.98 | 0.99 | 1 |
| gibbon | DivE | 0.74 | 0.4 | 0.83 | 0.85 | 0.92 | 0.91 | 0.96 | 0.96 | 0.98 | 0.99 |
|  | dless | 0.56 | 0.05 | 0.86 | 0.74 | 0.92 | 0.92 | 0.96 | 0.95 | 0.99 | 0.99 |
| human - gibbon | DivE | 0.64 | 0.4 | 0.78 | 0.88 | 0.89 | 0.95 | 0.94 | 0.98 | 0.99 | 0.99 |
|  | dless | 0.61 | 0.05 | 0.76 | 0.62 | 0.9 | 0.92 | 0.97 | 0.99 | 0.99 | 1 |
| colobus monkey | DivE | 0.75 | 0.48 | 0.86 | 0.86 | 0.91 | 0.9 | 0.95 | 0.96 | 0.98 | 0.98 |
|  | dless | 0.76 | 0.1 | 0.88 | 0.8 | 0.91 | 0.94 | 0.96 | 0.98 | 0.98 | 0.97 |
| vervet | DivE | 0.73 | 0.56 | 0.85 | 0.85 | 0.93 | 0.91 | 0.94 | 0.93 | 0.98 | 0.97 |
|  | dless | 0.5 | 0.09 | 0.87 | 0.8 | 0.92 | 0.95 | 0.95 | 0.97 | 0.97 | 0.93 |
| baboon | DivE | 0.77 | 0.52 | 0.84 | 0.84 | 0.92 | 0.9 | 0.95 | 0.94 | 0.98 | 0.97 |
|  | dless | 0.73 | 0.17 | 0.86 | 0.8 | 0.91 | 0.87 | 0.94 | 0.95 | 0.98 | 0.96 |
| macaque | DivE | 0.75 | 0.5 | 0.86 | 0.85 | 0.91 | 0.9 | 0.96 | 0.95 | 0.98 | 0.97 |
|  | dless | 0.64 | 0.1 | 0.87 | 0.8 | 0.9 | 0.88 | 0.94 | 0.94 | 0.98 | 0.96 |
| baboon - macaque | DivE | 0.74 | 0.45 | 0.82 | 0.88 | 0.9 | 0.92 | 0.95 | 0.97 | 0.98 | 0.98 |
|  | dless | 0.55 | 0.07 | 0.83 | 0.72 | 0.88 | 0.96 | 0.94 | 0.98 | 0.99 | 0.98 |
| vervet - baboon | DivE | 0.65 | 0.41 | 0.8 | 0.86 | 0.86 | 0.93 | 0.95 | 0.98 | 0.98 | 0.99 |
|  | dless | 0.51 | 0.09 | 0.77 | 0.7 | 0.89 | 0.95 | 0.96 | 0.99 | 0.99 | 1 |
| colobus monkey - vervet | DivE | 0.65 | 0.31 | 0.8 | 0.87 | 0.89 | 0.95 | 0.95 | 0.98 | 0.99 | 0.99 |
|  | dless | 0.15 | 0.02 | 0.79 | 0.56 | 0.91 | 0.97 | 0.98 | 0.98 | 0.99 | 0.99 |
| human - colobus monkey | DivE | 0.46 | 0.14 | 0.7 | 0.77 | 0.84 | 0.95 | 0.96 | 0.97 | 0.99 | 0.99 |
|  | dless | 0 | 0 | 0.63 | 0.26 | 0.89 | 0.92 | 0.98 | 0.98 | 0.99 | 0.99 |
| dusky titi | DivE | 0.68 | 0.38 | 0.85 | 0.87 | 0.92 | 0.92 | 0.96 | 0.97 | 0.99 | 0.99 |
|  | dless | 0.41 | 0.07 | 0.88 | 0.71 | 0.93 | 0.95 | 0.98 | 0.97 | 0.99 | 1 |
| owl monkey | DivE | 0.68 | 0.42 | 0.87 | 0.88 | 0.92 | 0.92 | 0.97 | 0.96 | 0.99 | 0.99 |
|  | dless | 0.74 | 0.08 | 0.87 | 0.82 | 0.92 | 0.92 | 0.97 | 0.97 | 0.99 | 0.99 |
| marmoset | DivE | 0.76 | 0.43 | 0.89 | 0.87 | 0.93 | 0.92 | 0.97 | 0.97 | 0.99 | 0.99 |
|  | dless | 0.62 | 0.09 | 0.88 | 0.73 | 0.93 | 0.91 | 0.98 | 0.97 | 0.99 | 0.99 |
| squirrel monkey | DivE | 0.66 | 0.46 | 0.83 | 0.85 | 0.91 | 0.91 | 0.97 | 0.97 | 0.98 | 0.98 |
|  | dless | 0.74 | 0.08 | 0.86 | 0.75 | 0.91 | 0.91 | 0.98 | 0.96 | 0.99 | 0.98 |
| marmoset - squirrel monkey | DivE | 0.73 | 0.3 | 0.87 | 0.88 | 0.91 | 0.93 | 0.96 | 0.98 | 0.98 | 0.99 |
|  | dless | 0.59 | 0.06 | 0.85 | 0.7 | 0.93 | 0.96 | 0.97 | 0.99 | 0.99 | 1 |
| owl monkey - marmoset | DivE | 0.72 | 0.42 | 0.83 | 0.86 | 0.9 | 0.95 | 0.97 | 0.98 | 0.99 | 0.99 |
|  | dless | 0.62 | 0.05 | 0.82 | 0.57 | 0.92 | 0.97 | 0.98 | 0.98 | 0.99 | 0.99 |
| dusky titi - owl monkey | DivE | 0.67 | 0.25 | 0.84 | 0.78 | 0.91 | 0.96 | 0.97 | 0.98 | 0.99 | 0.99 |
|  | dless | 0.57 | 0.02 | 0.85 | 0.35 | 0.94 | 0.97 | 0.98 | 0.99 | 0.99 | 0.98 |
| human – dusky titi | DivE | 0.15 | 0.01 | 0.37 | 0.04 | 0.71 | 0.53 | 0.95 | 0.96 | 0.98 | 0.97 |
|  | dless | 0 | 0 | 0 | 0 | 0.72 | 0.08 | 0.96 | 0.92 | 0.97 | 0.98 |
| mouse lemur | DivE | 0.85 | 0.37 | 0.85 | 0.88 | 0.94 | 0.94 | 0.98 | 0.98 | 0.99 | 0.99 |
|  | dless | 0.8 | 0.04 | 0.88 | 0.66 | 0.95 | 0.96 | 0.98 | 0.98 | 0.99 | 0.98 |
| galago | DivE | 0.81 | 0.23 | 0.88 | 0.84 | 0.94 | 0.95 | 0.98 | 0.99 | 0.99 | 0.99 |
|  | dless | 0.02 | 0 | 0.89 | 0.48 | 0.95 | 0.96 | 0.98 | 0.99 | 0.99 | 1 |
| mouse lemur - galago | DivE | 0.75 | 0.14 | 0.84 | 0.64 | 0.91 | 0.94 | 0.97 | 0.98 | 0.99 | 0.99 |
|  | dless | 0 | 0 | 0.76 | 0.15 | 0.92 | 0.91 | 0.98 | 0.99 | 0.99 | 0.99 |

**Table S14.** Accuracy obtained by DivE and DLESS for the prediction of lost elements of different lengths in each clade^1^ of the phylogeny, when the selction strength is given by ρ=0.3.

| 0.4 loss | | **50 bp** | | **100 bp** | | **200 bp** | | **500 bp** | | **1000 bp** | |
| --- | --- | --- | --- | --- | --- | --- | --- | --- | --- | --- | --- |
| **tree** | **prog** | Pr | Re | Pr | Re | Pr | Re | Pr | Re | Pr | Re |
| human | DivE | 0.67 | 0.24 | 0.81 | 0.76 | 0.9 | 0.85 | 0.93 | 0.91 | 0.95 | 0.94 |
|  | dless | 0.41 | 0.04 | 0.86 | 0.44 | 0.9 | 0.87 | 0.93 | 0.87 | 0.95 | 0.93 |
| chimp | DivE | 0.72 | 0.21 | 0.8 | 0.67 | 0.89 | 0.85 | 0.94 | 0.9 | 0.96 | 0.93 |
|  | dless | 0.79 | 0.02 | 0.84 | 0.36 | 0.9 | 0.77 | 0.93 | 0.91 | 0.96 | 0.88 |
| human -chimp | DivE | 0.59 | 0.27 | 0.82 | 0.75 | 0.86 | 0.87 | 0.92 | 0.93 | 0.96 | 0.96 |
|  | dless | 0 | 0 | 0.8 | 0.34 | 0.87 | 0.78 | 0.94 | 0.94 | 0.99 | 0.95 |
| orangutan | DivE | 0.77 | 0.26 | 0.85 | 0.7 | 0.91 | 0.87 | 0.94 | 0.91 | 0.97 | 0.93 |
|  | dless | 0.8 | 0.06 | 0.87 | 0.31 | 0.92 | 0.8 | 0.96 | 0.89 | 0.98 | 0.92 |
| human -orangutan | DivE | 0.62 | 0.18 | 0.77 | 0.7 | 0.87 | 0.89 | 0.92 | 0.96 | 0.94 | 0.98 |
|  | dless | 0.28 | 0.02 | 0.72 | 0.31 | 0.87 | 0.79 | 0.96 | 0.94 | 0.99 | 0.98 |
| gibbon | DivE | 0.78 | 0.27 | 0.84 | 0.66 | 0.9 | 0.86 | 0.94 | 0.92 | 0.97 | 0.96 |
|  | dless | 0.64 | 0.03 | 0.81 | 0.45 | 0.91 | 0.82 | 0.96 | 0.88 | 0.99 | 0.97 |
| human - gibbon | DivE | 0.64 | 0.16 | 0.73 | 0.61 | 0.8 | 0.87 | 0.94 | 0.96 | 0.97 | 0.98 |
|  | dless | 0 | 0 | 0.75 | 0.14 | 0.87 | 0.75 | 0.98 | 0.96 | 0.99 | 0.97 |
| colobus monkey | DivE | 0.68 | 0.2 | 0.83 | 0.72 | 0.91 | 0.86 | 0.95 | 0.92 | 0.96 | 0.96 |
|  | dless | 0.82 | 0.04 | 0.86 | 0.32 | 0.91 | 0.8 | 0.96 | 0.91 | 0.98 | 0.94 |
| vervet | DivE | 0.64 | 0.21 | 0.84 | 0.75 | 0.9 | 0.86 | 0.93 | 0.91 | 0.96 | 0.94 |
|  | dless | 0.36 | 0.03 | 0.86 | 0.43 | 0.92 | 0.87 | 0.95 | 0.9 | 0.97 | 0.88 |
| baboon | DivE | 0.73 | 0.2 | 0.85 | 0.72 | 0.89 | 0.87 | 0.93 | 0.92 | 0.96 | 0.95 |
|  | dless | 0.81 | 0.05 | 0.87 | 0.29 | 0.91 | 0.79 | 0.94 | 0.88 | 0.97 | 0.92 |
| macaque | DivE | 0.69 | 0.2 | 0.84 | 0.75 | 0.9 | 0.85 | 0.94 | 0.91 | 0.96 | 0.94 |
|  | dless | 0.83 | 0.02 | 0.86 | 0.42 | 0.91 | 0.82 | 0.95 | 0.89 | 0.97 | 0.86 |
| baboon - macaque | DivE | 0.66 | 0.21 | 0.81 | 0.66 | 0.88 | 0.88 | 0.92 | 0.93 | 0.96 | 0.97 |
|  | dless | 0.22 | 0.01 | 0.83 | 0.27 | 0.87 | 0.78 | 0.94 | 0.93 | 0.99 | 0.96 |
| vervet - baboon | DivE | 0.63 | 0.15 | 0.77 | 0.71 | 0.83 | 0.88 | 0.92 | 0.96 | 0.96 | 0.98 |
|  | dless | 0.75 | 0.02 | 0.74 | 0.26 | 0.86 | 0.81 | 0.97 | 0.95 | 0.99 | 0.96 |
| colobus monkey - vervet | DivE | 0.65 | 0.14 | 0.77 | 0.63 | 0.86 | 0.88 | 0.95 | 0.96 | 0.98 | 0.98 |
|  | dless | 0.18 | 0.02 | 0.72 | 0.15 | 0.93 | 0.69 | 0.98 | 0.95 | 0.99 | 0.98 |
| human - colobus monkey | DivE | 0.35 | 0.03 | 0.58 | 0.36 | 0.76 | 0.87 | 0.94 | 0.96 | 0.99 | 0.98 |
|  | dless | 0.37 | 0.01 | 0.61 | 0.06 | 0.85 | 0.58 | 0.99 | 0.95 | 0.99 | 0.96 |
| dusky titi | DivE | 0.71 | 0.16 | 0.85 | 0.7 | 0.9 | 0.86 | 0.95 | 0.94 | 0.98 | 0.97 |
|  | dless | 0.69 | 0.03 | 0.88 | 0.26 | 0.93 | 0.78 | 0.98 | 0.96 | 0.99 | 0.95 |
| owl monkey | DivE | 0.73 | 0.28 | 0.86 | 0.72 | 0.91 | 0.85 | 0.94 | 0.93 | 0.98 | 0.97 |
|  | dless | 0.63 | 0.05 | 0.87 | 0.42 | 0.92 | 0.8 | 0.97 | 0.9 | 0.99 | 0.95 |
| marmoset | DivE | 0.69 | 0.29 | 0.88 | 0.66 | 0.91 | 0.85 | 0.95 | 0.93 | 0.98 | 0.97 |
|  | dless | 0.29 | 0.02 | 0.84 | 0.3 | 0.93 | 0.82 | 0.98 | 0.92 | 0.99 | 0.96 |
| squirrel monkey | DivE | 0.74 | 0.26 | 0.83 | 0.69 | 0.91 | 0.85 | 0.96 | 0.93 | 0.97 | 0.97 |
|  | dless | 0.58 | 0.02 | 0.9 | 0.33 | 0.93 | 0.76 | 0.98 | 0.94 | 0.99 | 0.95 |
| marmoset - squirrel monkey | DivE | 0.63 | 0.17 | 0.83 | 0.65 | 0.89 | 0.9 | 0.95 | 0.96 | 0.98 | 0.97 |
|  | dless | 0.21 | 0.01 | 0.82 | 0.21 | 0.91 | 0.78 | 0.98 | 0.94 | 0.99 | 0.97 |
| owl monkey - marmoset | DivE | 0.6 | 0.13 | 0.82 | 0.68 | 0.85 | 0.9 | 0.94 | 0.96 | 0.98 | 0.97 |
|  | dless | 0.68 | 0.01 | 0.83 | 0.25 | 0.9 | 0.71 | 0.98 | 0.97 | 0.99 | 0.98 |
| dusky titi - owl monkey | DivE | 0.63 | 0.1 | 0.78 | 0.5 | 0.88 | 0.88 | 0.95 | 0.95 | 0.99 | 0.97 |
|  | dless | 0 | 0 | 0.86 | 0.09 | 0.92 | 0.67 | 0.98 | 0.95 | 0.99 | 0.97 |
| human – dusky titi | DivE | 0.15 | 0.01 | 0.15 | 0.03 | 0.61 | 0.21 | 0.9 | 0.87 | 0.95 | 0.94 |
|  | dless | 0 | 0 | 0 | 0 | 0.34 | 0.03 | 0.96 | 0.64 | 0.98 | 0.91 |
| mouse lemur | DivE | 0.75 | 0.17 | 0.85 | 0.64 | 0.91 | 0.89 | 0.97 | 0.95 | 0.99 | 0.98 |
|  | dless | 0.23 | 0.01 | 0.79 | 0.26 | 0.93 | 0.8 | 0.98 | 0.93 | 0.99 | 0.98 |
| galago | DivE | 0.71 | 0.11 | 0.85 | 0.54 | 0.91 | 0.86 | 0.97 | 0.96 | 0.98 | 0.98 |
|  | dless | 0.62 | 0.01 | 0.76 | 0.19 | 0.95 | 0.67 | 0.98 | 0.96 | 0.99 | 0.96 |
| mouse lemur - galago | DivE | 0.64 | 0.03 | 0.82 | 0.37 | 0.91 | 0.82 | 0.97 | 0.95 | 0.98 | 0.97 |
|  | dless | 0.13 | 0.01 | 0.87 | 0.05 | 0.94 | 0.45 | 0.98 | 0.96 | 0.99 | 0.97 |

**Table S15.** Accuracy obtained by DivE and DLESS for the prediction of lost elements of different lengths in each clade^1^ of the phylogeny, when the selction strength is given by ρ=0.4.

| 0.5 loss | | **50 bp** | | **100 bp** | | **200 bp** | | **500 bp** | | **1000 bp** | |
| --- | --- | --- | --- | --- | --- | --- | --- | --- | --- | --- | --- |
| **tree** | **prog** | Pr | Re | Pr | Re | Pr | Re | Pr | Re | Pr | Re |
| human | DivE | 0.58 | 0.13 | 0.85 | 0.44 | 0.91 | 0.73 | 0.93 | 0.86 | 0.94 | 0.9 |
|  | dless | 0.33 | 0.01 | 0.85 | 0.11 | 0.91 | 0.45 | 0.92 | 0.69 | 0.94 | 0.74 |
| chimp | DivE | 0.54 | 0.08 | 0.8 | 0.44 | 0.89 | 0.73 | 0.93 | 0.86 | 0.94 | 0.9 |
|  | dless | 0.8 | 0.01 | 0.77 | 0.14 | 0.92 | 0.42 | 0.92 | 0.66 | 0.95 | 0.71 |
| human -chimp | DivE | 0.63 | 0.02 | 0.78 | 0.44 | 0.85 | 0.76 | 0.9 | 0.88 | 0.94 | 0.93 |
|  | dless | 0 | 0 | 0.79 | 0.04 | 0.87 | 0.36 | 0.91 | 0.69 | 0.96 | 0.72 |
| orangutan | DivE | 0.61 | 0.09 | 0.82 | 0.43 | 0.86 | 0.76 | 0.93 | 0.87 | 0.96 | 0.92 |
|  | dless | 0.76 | 0.02 | 0.84 | 0.11 | 0.9 | 0.38 | 0.95 | 0.64 | 0.96 | 0.71 |
| human -orangutan | DivE | 0.65 | 0.05 | 0.75 | 0.31 | 0.83 | 0.77 | 0.91 | 0.9 | 0.94 | 0.94 |
|  | dless | 0.51 | 0.01 | 0.77 | 0.05 | 0.86 | 0.4 | 0.94 | 0.7 | 0.98 | 0.83 |
| gibbon | DivE | 0.64 | 0.09 | 0.79 | 0.38 | 0.89 | 0.74 | 0.94 | 0.86 | 0.96 | 0.92 |
|  | dless | 0.64 | 0.01 | 0.58 | 0.1 | 0.92 | 0.36 | 0.95 | 0.7 | 0.98 | 0.78 |
| human - gibbon | DivE | 0.48 | 0.07 | 0.67 | 0.28 | 0.8 | 0.73 | 0.88 | 0.89 | 0.96 | 0.95 |
|  | dless | 0 | 0 | 0.68 | 0.04 | 0.81 | 0.25 | 0.96 | 0.67 | 0.99 | 0.77 |
| colobus monkey | DivE | 0.72 | 0.08 | 0.81 | 0.48 | 0.9 | 0.77 | 0.93 | 0.85 | 0.96 | 0.92 |
|  | dless | 0.23 | 0.01 | 0.86 | 0.1 | 0.91 | 0.4 | 0.95 | 0.68 | 0.97 | 0.75 |
| vervet | DivE | 0.77 | 0.1 | 0.83 | 0.37 | 0.89 | 0.78 | 0.93 | 0.87 | 0.95 | 0.91 |
|  | dless | 0 | 0 | 0.74 | 0.09 | 0.92 | 0.45 | 0.94 | 0.68 | 0.96 | 0.72 |
| baboon | DivE | 0.71 | 0.09 | 0.81 | 0.37 | 0.89 | 0.78 | 0.93 | 0.85 | 0.94 | 0.89 |
|  | dless | 0.83 | 0.01 | 0.91 | 0.09 | 0.92 | 0.41 | 0.94 | 0.7 | 0.95 | 0.79 |
| macaque | DivE | 0.65 | 0.08 | 0.85 | 0.43 | 0.91 | 0.74 | 0.92 | 0.86 | 0.95 | 0.9 |
|  | dless | 0 | 0 | 0.82 | 0.11 | 0.91 | 0.36 | 0.94 | 0.67 | 0.96 | 0.76 |
| baboon - macaque | DivE | 0.56 | 0.06 | 0.75 | 0.44 | 0.85 | 0.7 | 0.91 | 0.86 | 0.93 | 0.93 |
|  | dless | 0 | 0 | 0.79 | 0.06 | 0.89 | 0.38 | 0.93 | 0.69 | 0.96 | 0.75 |
| vervet - baboon | DivE | 0.45 | 0.07 | 0.71 | 0.31 | 0.83 | 0.74 | 0.91 | 0.91 | 0.95 | 0.96 |
|  | dless | 0.02 | 0 | 0.41 | 0.06 | 0.86 | 0.31 | 0.95 | 0.73 | 0.98 | 0.83 |
| colobus monkey - vervet | DivE | 0.45 | 0.08 | 0.67 | 0.24 | 0.82 | 0.69 | 0.93 | 0.9 | 0.97 | 0.96 |
|  | dless | 0 | 0 | 0.72 | 0.04 | 0.85 | 0.28 | 0.98 | 0.66 | 0.99 | 0.86 |
| human - colobus monkey | DivE | 0.53 | 0.01 | 0.51 | 0.16 | 0.71 | 0.52 | 0.9 | 0.89 | 0.98 | 0.94 |
|  | dless | 0.35 | 0 | 0 | 0 | 0.78 | 0.16 | 0.97 | 0.6 | 0.99 | 0.83 |
| dusky titi | DivE | 0.59 | 0.1 | 0.81 | 0.45 | 0.87 | 0.72 | 0.94 | 0.87 | 0.97 | 0.94 |
|  | dless | 0 | 0 | 0.87 | 0.04 | 0.9 | 0.34 | 0.96 | 0.66 | 0.98 | 0.76 |
| owl monkey | DivE | 0.63 | 0.08 | 0.82 | 0.38 | 0.88 | 0.73 | 0.94 | 0.87 | 0.96 | 0.93 |
|  | dless | 0.37 | 0.04 | 0.81 | 0.06 | 0.9 | 0.36 | 0.96 | 0.68 | 0.98 | 0.75 |
| marmoset | DivE | 0.53 | 0.06 | 0.86 | 0.39 | 0.92 | 0.74 | 0.94 | 0.88 | 0.96 | 0.93 |
|  | dless | 0 | 0 | 0.85 | 0.1 | 0.92 | 0.36 | 0.97 | 0.66 | 0.99 | 0.81 |
| squirrel monkey | DivE | 0.67 | 0.03 | 0.82 | 0.41 | 0.87 | 0.7 | 0.94 | 0.87 | 0.97 | 0.94 |
|  | dless | 0.5 | 0.01 | 0.76 | 0.1 | 0.94 | 0.35 | 0.97 | 0.7 | 0.99 | 0.77 |
| marmoset - squirrel monkey | DivE | 0.67 | 0.06 | 0.76 | 0.39 | 0.87 | 0.71 | 0.95 | 0.9 | 0.97 | 0.94 |
|  | dless | 0 | 0 | 0.74 | 0.07 | 0.92 | 0.28 | 0.98 | 0.77 | 0.99 | 0.85 |
| owl monkey - marmoset | DivE | 0.21 | 0.01 | 0.81 | 0.29 | 0.85 | 0.73 | 0.93 | 0.87 | 0.97 | 0.95 |
|  | dless | 0.02 | 0 | 0.73 | 0.05 | 0.89 | 0.29 | 0.97 | 0.64 | 0.99 | 0.85 |
| dusky titi - owl monkey | DivE | 0.39 | 0.03 | 0.68 | 0.15 | 0.86 | 0.65 | 0.96 | 0.9 | 0.98 | 0.95 |
|  | dless | 0 | 0 | 0.65 | 0.03 | 0.89 | 0.16 | 0.98 | 0.63 | 0.99 | 0.83 |
| human – dusky titi | DivE | 0 | 0 | 0.71 | 0.01 | 0.62 | 0.09 | 0.9 | 0.58 | 0.96 | 0.87 |
|  | dless | 0 | 0 | 0 | 0 | 0 | 0 | 0.96 | 0.15 | 0.98 | 0.46 |
| mouse lemur | DivE | 0.64 | 0.05 | 0.76 | 0.28 | 0.9 | 0.75 | 0.95 | 0.9 | 0.98 | 0.95 |
|  | dless | 0.02 | 0 | 0.81 | 0.03 | 0.95 | 0.33 | 0.98 | 0.78 | 0.99 | 0.87 |
| galago | DivE | 0.69 | 0.04 | 0.8 | 0.36 | 0.86 | 0.69 | 0.96 | 0.91 | 0.98 | 0.95 |
|  | dless | 0.93 | 0.01 | 0.84 | 0.03 | 0.94 | 0.24 | 0.98 | 0.75 | 1 | 0.86 |
| mouse lemur - galago | DivE | 0.74 | 0.01 | 0.7 | 0.08 | 0.89 | 0.51 | 0.96 | 0.86 | 0.98 | 0.93 |
|  | dless | 0.02 | 0 | 0.05 | 0 | 0.92 | 0.12 | 0.98 | 0.59 | 0.99 | 0.79 |

**Table S16.** Accuracy obtained by DivE and DLESS for the prediction of lost elements of different lengths in each clade^1^ of the phylogeny, when the selction strength is given by ρ=0.5.

| 0.01^-1^ acc | **50 bp** | | **100 bp** | | **200 bp** | | **500 bp** | | **1000 bp** | |
| --- | --- | --- | --- | --- | --- | --- | --- | --- | --- | --- |
| **tree** | Pr | Re | Pr | Re | Pr | Re | Pr | Re | Pr | Re |
| human | 0.2 | 0.96 | 0.47 | 0.98 | 0.74 | 0.99 | 0.81 | 1 | 0.88 | 1 |
| chimp | 0.29 | 0.97 | 0.55 | 0.99 | 0.75 | 0.99 | 0.83 | 1 | 0.91 | 1 |
| human -chimp | 0.37 | 0.98 | 0.65 | 0.99 | 0.76 | 0.99 | 0.86 | 1 | 0.92 | 1 |
| orangutan | 0.51 | 0.98 | 0.65 | 0.99 | 0.77 | 0.99 | 0.9 | 1 | 0.94 | 1 |
| human -orangutan | 0.36 | 0.92 | 0.44 | 0.96 | 0.59 | 0.98 | 0.83 | 0.99 | 0.88 | 1 |
| gibbon | 0.46 | 0.98 | 0.61 | 0.99 | 0.79 | 1 | 0.9 | 1 | 0.94 | 1 |
| human - gibbon | 0.39 | 0.98 | 0.56 | 0.99 | 0.77 | 0.99 | 0.83 | 1 | 0.91 | 1 |
| colobus monkey | 0.51 | 0.98 | 0.69 | 0.99 | 0.78 | 1 | 0.91 | 1 | 0.89 | 1 |
| vervet | 0.48 | 0.98 | 0.55 | 0.99 | 0.68 | 0.99 | 0.86 | 1 | 0.93 | 1 |
| baboon | 0.5 | 0.98 | 0.56 | 0.99 | 0.69 | 1 | 0.9 | 1 | 0.92 | 1 |
| macaque | 0.32 | 0.97 | 0.54 | 0.99 | 0.8 | 0.99 | 0.87 | 1 | 0.91 | 1 |
| baboon – macaque | 0.29 | 0.93 | 0.44 | 0.97 | 0.65 | 0.99 | 0.85 | 0.99 | 0.88 | 1 |
| vervet - baboon | 0.29 | 0.98 | 0.59 | 0.99 | 0.69 | 0.99 | 0.84 | 1 | 0.9 | 1 |
| colobus monkey - vervet | 0.52 | 0.98 | 0.63 | 0.99 | 0.81 | 1 | 0.92 | 1 | 0.96 | 1 |
| human - colobus monkey | 0.5 | 0.98 | 0.61 | 0.99 | 0.77 | 1 | 0.87 | 1 | 0.94 | 1 |
| dusky titi | 0.58 | 0.98 | 0.67 | 0.99 | 0.84 | 0.99 | 0.94 | 1 | 0.93 | 1 |
| owl monkey | 0.48 | 0.98 | 0.7 | 0.99 | 0.71 | 1 | 0.88 | 1 | 0.97 | 1 |
| marmoset | 0.5 | 0.98 | 0.75 | 0.99 | 0.83 | 1 | 0.84 | 1 | 0.96 | 1 |
| squirrel monkey | 0.54 | 0.98 | 0.69 | 0.99 | 0.88 | 1 | 0.94 | 1 | 0.97 | 1 |
| marmoset - squirrel monkey | 0.23 | 0.72 | 0.43 | 0.95 | 0.57 | 0.97 | 0.74 | 0.99 | 0.83 | 0.99 |
| owl monkey - marmoset | 0.26 | 0.95 | 0.48 | 0.98 | 0.63 | 0.99 | 0.81 | 1 | 0.92 | 1 |
| dusky titi - owl monkey | 0.61 | 0.98 | 0.7 | 0.99 | 0.92 | 1 | 0.94 | 1 | 0.97 | 1 |
| human – dusky titi | 0.53 | 0.98 | 0.75 | 0.99 | 0.86 | 1 | 0.94 | 1 | 0.96 | 1 |
| mouse lemur | 0.74 | 0.98 | 0.87 | 0.99 | 0.9 | 0.99 | 0.93 | 1 | 0.98 | 1 |
| galago | 0.86 | 0.97 | 0.87 | 0.99 | 0.93 | 0.99 | 0.95 | 1 | 0.98 | 1 |
| mouse lemur - galago | 0.61 | 0.98 | 0.71 | 0.99 | 0.83 | 1 | 0.9 | 1 | 0.97 | 1 |

**Table S17.** Accuracy obtained by DivE for the prediction of lost elements of different lengths in each clade^1^ of the phylogeny, when the selction strength is given by ρ=0.01^-1^.

| 0.02^-1^ acc | **50 bp** | | **100 bp** | | **200 bp** | | **500 bp** | | **1000 bp** | |
| --- | --- | --- | --- | --- | --- | --- | --- | --- | --- | --- |
| **tree** | Pr | Re | Pr | Re | Pr | Re | Pr | Re | Pr | Re |
| human | 0.35 | 0.92 | 0.55 | 0.97 | 0.61 | 0.98 | 0.88 | 0.99 | 0.87 | 1 |
| chimp | 0.44 | 0.93 | 0.57 | 0.97 | 0.66 | 0.99 | 0.89 | 0.99 | 0.89 | 1 |
| human -chimp | 0.38 | 0.95 | 0.56 | 0.98 | 0.71 | 0.99 | 0.83 | 1 | 0.88 | 1 |
| orangutan | 0.51 | 0.97 | 0.63 | 0.99 | 0.74 | 0.99 | 0.9 | 1 | 0.96 | 1 |
| human -orangutan | 0.29 | 0.58 | 0.41 | 0.91 | 0.66 | 0.96 | 0.81 | 0.98 | 0.86 | 0.99 |
| gibbon | 0.46 | 0.98 | 0.62 | 0.99 | 0.76 | 0.99 | 0.9 | 1 | 0.94 | 1 |
| human - gibbon | 0.35 | 0.96 | 0.65 | 0.98 | 0.7 | 0.99 | 0.83 | 1 | 0.93 | 1 |
| colobus monkey | 0.6 | 0.97 | 0.6 | 0.99 | 0.81 | 0.99 | 0.92 | 1 | 0.92 | 1 |
| vervet | 0.47 | 0.96 | 0.62 | 0.98 | 0.72 | 0.99 | 0.85 | 1 | 0.91 | 1 |
| baboon | 0.47 | 0.96 | 0.58 | 0.98 | 0.72 | 0.99 | 0.8 | 1 | 0.96 | 1 |
| macaque | 0.41 | 0.96 | 0.45 | 0.99 | 0.79 | 0.99 | 0.89 | 1 | 0.93 | 1 |
| baboon - macaque | 0.37 | 0.71 | 0.56 | 0.93 | 0.67 | 0.97 | 0.77 | 0.99 | 0.88 | 0.99 |
| vervet - baboon | 0.39 | 0.94 | 0.53 | 0.97 | 0.67 | 0.98 | 0.84 | 0.99 | 0.88 | 1 |
| colobus monkey - vervet | 0.55 | 0.98 | 0.76 | 0.99 | 0.8 | 0.99 | 0.91 | 1 | 0.96 | 1 |
| human - colobus monkey | 0.53 | 0.97 | 0.6 | 0.99 | 0.78 | 0.99 | 0.91 | 1 | 0.93 | 1 |
| dusky titi | 0.67 | 0.98 | 0.79 | 0.99 | 0.8 | 1 | 0.94 | 1 | 0.97 | 1 |
| owl monkey | 0.57 | 0.98 | 0.73 | 0.99 | 0.81 | 0.99 | 0.84 | 1 | 0.95 | 1 |
| marmoset | 0.52 | 0.98 | 0.76 | 0.99 | 0.79 | 0.99 | 0.86 | 1 | 0.96 | 1 |
| squirrel monkey | 0.62 | 0.98 | 0.67 | 0.99 | 0.84 | 0.99 | 0.91 | 1 | 0.95 | 1 |
| marmoset - squirrel monkey | 0.15 | 0.22 | 0.32 | 0.74 | 0.66 | 0.94 | 0.77 | 0.98 | 0.85 | 0.99 |
| owl monkey - marmoset | 0.28 | 0.88 | 0.56 | 0.96 | 0.68 | 0.98 | 0.79 | 0.99 | 0.85 | 1 |
| dusky titi - owl monkey | 0.44 | 0.98 | 0.68 | 0.99 | 0.79 | 1 | 0.93 | 1 | 0.96 | 1 |
| human – dusky titi | 0.55 | 0.98 | 0.71 | 0.99 | 0.8 | 0.99 | 0.94 | 1 | 0.96 | 1 |
| mouse lemur | 0.76 | 0.98 | 0.83 | 0.99 | 0.93 | 0.99 | 0.95 | 1 | 0.95 | 1 |
| galago | 0.81 | 0.98 | 0.81 | 0.99 | 0.88 | 0.99 | 0.97 | 1 | 0.96 | 1 |
| mouse lemur - galago | 0.58 | 0.98 | 0.74 | 0.99 | 0.84 | 0.99 | 0.93 | 1 | 0.97 | 1 |

**Table S18.** Accuracy obtained by DivE for the prediction of lost elements of different lengths in each clade^1^ of the phylogeny, when the selction strength is given by ρ=0.02^-1^.

| 0.05^-1^ acc | **50 bp** | | **100 bp** | | **200 bp** | | **500 bp** | | **1000 bp** | |
| --- | --- | --- | --- | --- | --- | --- | --- | --- | --- | --- |
| **tree** | Pr | Re | Pr | Re | Pr | Re | Pr | Re | Pr | Re |
| human | 0.28 | 0.27 | 0.45 | 0.81 | 0.64 | 0.95 | 0.79 | 0.98 | 0.9 | 0.99 |
| chimp | 0.23 | 0.5 | 0.58 | 0.91 | 0.71 | 0.96 | 0.9 | 0.98 | 0.91 | 0.99 |
| human -chimp | 0.36 | 0.65 | 0.52 | 0.94 | 0.75 | 0.96 | 0.83 | 0.99 | 0.95 | 0.99 |
| orangutan | 0.39 | 0.93 | 0.66 | 0.97 | 0.84 | 0.98 | 0.88 | 0.99 | 0.93 | 1 |
| human -orangutan | 0.03 | 0.03 | 0.52 | 0.36 | 0.59 | 0.81 | 0.75 | 0.96 | 0.88 | 0.98 |
| gibbon | 0.48 | 0.96 | 0.77 | 0.98 | 0.77 | 0.98 | 0.83 | 1 | 0.89 | 1 |
| human - gibbon | 0.35 | 0.69 | 0.46 | 0.94 | 0.69 | 0.97 | 0.86 | 0.99 | 0.94 | 1 |
| colobus monkey | 0.34 | 0.93 | 0.65 | 0.97 | 0.8 | 0.98 | 0.9 | 0.99 | 0.96 | 1 |
| vervet | 0.45 | 0.75 | 0.65 | 0.95 | 0.71 | 0.97 | 0.82 | 0.99 | 0.95 | 0.99 |
| baboon | 0.3 | 0.77 | 0.58 | 0.95 | 0.69 | 0.97 | 0.88 | 0.99 | 0.91 | 0.99 |
| macaque | 0.36 | 0.75 | 0.5 | 0.93 | 0.69 | 0.97 | 0.86 | 0.99 | 0.9 | 1 |
| baboon - macaque | 0.14 | 0.1 | 0.32 | 0.52 | 0.52 | 0.86 | 0.81 | 0.96 | 0.85 | 0.98 |
| vervet - baboon | 0.28 | 0.4 | 0.53 | 0.89 | 0.72 | 0.95 | 0.78 | 0.98 | 0.9 | 0.99 |
| colobus monkey - vervet | 0.61 | 0.94 | 0.65 | 0.97 | 0.76 | 0.99 | 0.89 | 1 | 0.96 | 1 |
| human - colobus monkey | 0.46 | 0.87 | 0.64 | 0.97 | 0.82 | 0.98 | 0.91 | 0.99 | 0.95 | 1 |
| dusky titi | 0.57 | 0.96 | 0.72 | 0.98 | 0.86 | 0.99 | 0.93 | 1 | 0.95 | 1 |
| owl monkey | 0.28 | 0.95 | 0.67 | 0.97 | 0.82 | 0.99 | 0.94 | 0.99 | 0.96 | 1 |
| marmoset | 0.48 | 0.96 | 0.7 | 0.98 | 0.84 | 0.99 | 0.94 | 0.99 | 0.95 | 1 |
| squirrel monkey | 0.62 | 0.95 | 0.7 | 0.98 | 0.82 | 0.99 | 0.94 | 1 | 0.95 | 1 |
| marmoset - squirrel monkey | 0 | 0 | 0.16 | 0.04 | 0.54 | 0.43 | 0.74 | 0.92 | 0.82 | 0.97 |
| owl monkey - marmoset | 0.14 | 0.2 | 0.54 | 0.74 | 0.64 | 0.95 | 0.76 | 0.98 | 0.86 | 0.99 |
| dusky titi - owl monkey | 0.64 | 0.96 | 0.66 | 0.98 | 0.82 | 0.99 | 0.92 | 1 | 0.95 | 1 |
| human – dusky titi | 0.5 | 0.97 | 0.75 | 0.98 | 0.83 | 0.99 | 0.91 | 1 | 0.97 | 1 |
| mouse lemur | 0.64 | 0.97 | 0.8 | 0.99 | 0.92 | 0.99 | 0.9 | 1 | 0.95 | 1 |
| galago | 0.64 | 0.98 | 0.85 | 0.99 | 0.92 | 0.99 | 0.98 | 1 | 0.96 | 1 |
| mouse lemur - galago | 0.44 | 0.86 | 0.62 | 0.97 | 0.83 | 0.99 | 0.88 | 0.99 | 0.96 | 1 |

**Table S19.** Accuracy obtained by DivE for the prediction of lost elements of different lengths in each clade^1^ of the phylogeny, when the selction strength is given by ρ=0.05^-1^.

| 0.1^-1^ acc | **50 bp** | | **100 bp** | | **200 bp** | | **500 bp** | | **1000 bp** | |
| --- | --- | --- | --- | --- | --- | --- | --- | --- | --- | --- |
| **tree** | Pr | Re | Pr | Re | Pr | Re | Pr | Re | Pr | Re |
| human | 0.02 | 0.01 | 0.29 | 0.18 | 0.59 | 0.6 | 0.79 | 0.94 | 0.82 | 0.97 |
| chimp | 0.19 | 0.08 | 0.42 | 0.31 | 0.69 | 0.84 | 0.81 | 0.96 | 0.91 | 0.98 |
| human -chimp | 0.46 | 0.14 | 0.47 | 0.62 | 0.7 | 0.88 | 0.82 | 0.98 | 0.91 | 0.98 |
| orangutan | 0.42 | 0.34 | 0.63 | 0.85 | 0.78 | 0.96 | 0.9 | 0.98 | 0.92 | 0.99 |
| human -orangutan | 0 | 0 | 0.16 | 0.05 | 0.64 | 0.19 | 0.75 | 0.76 | 0.86 | 0.94 |
| gibbon | 0.48 | 0.52 | 0.46 | 0.93 | 0.73 | 0.98 | 0.91 | 0.99 | 0.97 | 0.99 |
| human - gibbon | 0.18 | 0.1 | 0.56 | 0.53 | 0.69 | 0.88 | 0.86 | 0.98 | 0.94 | 0.98 |
| colobus monkey | 0.35 | 0.43 | 0.72 | 0.87 | 0.81 | 0.96 | 0.9 | 0.98 | 0.94 | 0.99 |
| vervet | 0.21 | 0.26 | 0.47 | 0.76 | 0.77 | 0.93 | 0.88 | 0.98 | 0.94 | 0.99 |
| baboon | 0.33 | 0.15 | 0.45 | 0.61 | 0.74 | 0.94 | 0.86 | 0.97 | 0.94 | 0.99 |
| macaque | 0.35 | 0.14 | 0.51 | 0.62 | 0.67 | 0.93 | 0.9 | 0.97 | 0.95 | 0.98 |
| baboon - macaque | 0 | 0 | 0.42 | 0.04 | 0.67 | 0.32 | 0.79 | 0.83 | 0.89 | 0.96 |
| vervet - baboon | 0.31 | 0.03 | 0.43 | 0.32 | 0.68 | 0.77 | 0.8 | 0.96 | 0.82 | 0.98 |
| colobus monkey - vervet | 0.49 | 0.56 | 0.67 | 0.86 | 0.77 | 0.96 | 0.9 | 0.99 | 0.96 | 0.99 |
| human - colobus monkey | 0.4 | 0.34 | 0.64 | 0.82 | 0.71 | 0.95 | 0.91 | 0.98 | 0.95 | 0.99 |
| dusky titi | 0.57 | 0.69 | 0.73 | 0.94 | 0.85 | 0.97 | 0.93 | 0.99 | 0.97 | 0.99 |
| owl monkey | 0.49 | 0.58 | 0.48 | 0.92 | 0.82 | 0.97 | 0.93 | 0.99 | 0.96 | 0.99 |
| marmoset | 0.57 | 0.76 | 0.71 | 0.95 | 0.79 | 0.98 | 0.92 | 0.99 | 0.87 | 0.99 |
| squirrel monkey | 0.56 | 0.75 | 0.63 | 0.93 | 0.82 | 0.97 | 0.9 | 0.99 | 0.92 | 0.99 |
| marmoset - squirrel monkey | 0 | 0 | 0 | 0 | 0.78 | 0.08 | 0.68 | 0.54 | 0.86 | 0.88 |
| owl monkey - marmoset | 0 | 0 | 0.35 | 0.12 | 0.54 | 0.66 | 0.79 | 0.91 | 0.87 | 0.97 |
| dusky titi - owl monkey | 0.54 | 0.68 | 0.65 | 0.94 | 0.82 | 0.98 | 0.89 | 0.99 | 0.96 | 0.99 |
| human – dusky titi | 0.44 | 0.8 | 0.61 | 0.96 | 0.78 | 0.98 | 0.96 | 0.99 | 0.94 | 1 |
| mouse lemur | 0.71 | 0.94 | 0.81 | 0.97 | 0.9 | 0.99 | 0.94 | 0.99 | 0.95 | 1 |
| galago | 0.84 | 0.95 | 0.86 | 0.98 | 0.91 | 0.99 | 0.97 | 1 | 0.98 | 1 |
| mouse lemur - galago | 0.26 | 0.22 | 0.6 | 0.76 | 0.66 | 0.96 | 0.89 | 0.98 | 0.94 | 0.99 |

**Table S20.** Accuracy obtained by DivE for the prediction of lost elements of different lengths in each clade^1^ of the phylogeny, when the selction strength is given by ρ=0.1^-1^.

| 0.2^-1^ acc | **50 bp** | | **100 bp** | | **200 bp** | | **500 bp** | | **1000 bp** | |
| --- | --- | --- | --- | --- | --- | --- | --- | --- | --- | --- |
| **tree** | Pr | Re | Pr | Re | Pr | Re | Pr | Re | Pr | Re |
| human | 0 | 0 | 0 | 0 | 0.34 | 0.08 | 0.66 | 0.43 | 0.83 | 0.9 |
| chimp | 0.23 | 0.02 | 1 | 0.02 | 0.66 | 0.13 | 0.64 | 0.7 | 0.89 | 0.92 |
| human -chimp | 0 | 0 | 0.37 | 0.04 | 0.43 | 0.24 | 0.85 | 0.84 | 0.9 | 0.95 |
| orangutan | 0.29 | 0.04 | 0.43 | 0.2 | 0.71 | 0.56 | 0.87 | 0.94 | 0.93 | 0.98 |
| human -orangutan | 0 | 0 | 0 | 0 | 0 | 0 | 0.59 | 0.07 | 0.84 | 0.44 |
| gibbon | 0.17 | 0.04 | 0.59 | 0.19 | 0.78 | 0.78 | 0.88 | 0.96 | 0.94 | 0.97 |
| human - gibbon | 0.87 | 0.01 | 0.2 | 0.03 | 0.56 | 0.2 | 0.77 | 0.83 | 0.9 | 0.96 |
| colobus monkey | 0.34 | 0.04 | 0.5 | 0.17 | 0.68 | 0.64 | 0.85 | 0.93 | 0.93 | 0.98 |
| vervet | 0 | 0 | 0.49 | 0.13 | 0.71 | 0.42 | 0.88 | 0.87 | 0.94 | 0.95 |
| baboon | 0.58 | 0.01 | 0.42 | 0.03 | 0.67 | 0.29 | 0.85 | 0.87 | 0.92 | 0.96 |
| macaque | 0.04 | 0.01 | 0.69 | 0.04 | 0.6 | 0.37 | 0.81 | 0.86 | 0.91 | 0.95 |
| baboon - macaque | 0 | 0 | 0 | 0 | 0.88 | 0.01 | 0.79 | 0.27 | 0.81 | 0.67 |
| vervet - baboon | 0.04 | 0.01 | 0.23 | 0.04 | 0.2 | 0.07 | 0.73 | 0.66 | 0.86 | 0.93 |
| colobus monkey - vervet | 0.25 | 0.06 | 0.46 | 0.32 | 0.79 | 0.82 | 0.91 | 0.96 | 0.95 | 0.98 |
| human - colobus monkey | 0.26 | 0.03 | 0.37 | 0.16 | 0.68 | 0.61 | 0.86 | 0.93 | 0.94 | 0.97 |
| dusky titi | 0.33 | 0.09 | 0.45 | 0.39 | 0.83 | 0.87 | 0.91 | 0.96 | 0.89 | 0.99 |
| owl monkey | 0.07 | 0.09 | 0.53 | 0.34 | 0.76 | 0.77 | 0.91 | 0.96 | 0.93 | 0.97 |
| marmoset | 0.59 | 0.08 | 0.55 | 0.46 | 0.84 | 0.9 | 0.93 | 0.97 | 0.95 | 0.98 |
| squirrel monkey | 0.6 | 0.09 | 0.57 | 0.44 | 0.81 | 0.87 | 0.93 | 0.96 | 0.86 | 0.99 |
| marmoset - squirrel monkey | 0 | 0 | 0 | 0 | 0.14 | 0.01 | 1 | 0.03 | 0.81 | 0.17 |
| owl monkey - marmoset | 0 | 0 | 0.52 | 0.01 | 0.36 | 0.08 | 0.83 | 0.32 | 0.87 | 0.83 |
| dusky titi - owl monkey | 0.2 | 0.06 | 0.56 | 0.34 | 0.79 | 0.82 | 0.91 | 0.96 | 0.97 | 0.98 |
| human – dusky titi | 0.23 | 0.13 | 0.55 | 0.67 | 0.72 | 0.91 | 0.88 | 0.98 | 0.91 | 0.99 |
| mouse lemur | 0.46 | 0.24 | 0.78 | 0.81 | 0.86 | 0.95 | 0.94 | 0.98 | 0.95 | 0.99 |
| galago | 0.56 | 0.49 | 0.84 | 0.92 | 0.91 | 0.97 | 0.96 | 0.98 | 0.98 | 0.99 |
| mouse lemur - galago | 0.85 | 0.01 | 0.35 | 0.09 | 0.65 | 0.46 | 0.81 | 0.91 | 0.93 | 0.95 |

**Table S21.** Accuracy obtained by DivE for the prediction of lost elements of different lengths in each clade^1^ of the phylogeny, when the selction strength is given by ρ=0.2^-1^.

| 0.3^-1^ acc | **50 bp** | | **100 bp** | | **200 bp** | | **500 bp** | | **1000 bp** | |
| --- | --- | --- | --- | --- | --- | --- | --- | --- | --- | --- |
| **tree** | Pr | Re | Pr | Re | Pr | Re | Pr | Re | Pr | Re |
| human | 0.01 | 0 | 0 | 0 | 0.44 | 0.03 | 0.58 | 0.11 | 0.85 | 0.38 |
| chimp | 0 | 0 | 1 | 0.01 | 0.28 | 0.02 | 0.53 | 0.17 | 0.86 | 0.59 |
| human -chimp | 0 | 0 | 0 | 0 | 0.68 | 0.05 | 0.59 | 0.28 | 0.86 | 0.72 |
| orangutan | 0 | 0 | 0.52 | 0.01 | 0.89 | 0.17 | 0.87 | 0.74 | 0.84 | 0.93 |
| human -orangutan | 0 | 0 | 0 | 0 | 0 | 0 | 0 | 0 | 0.73 | 0.06 |
| gibbon | 0.14 | 0.01 | 0.57 | 0.05 | 0.36 | 0.22 | 0.84 | 0.82 | 0.94 | 0.94 |
| human - gibbon | 0.07 | 0.01 | 0.22 | 0.02 | 0.22 | 0.04 | 0.8 | 0.32 | 0.89 | 0.76 |
| colobus monkey | 0 | 0 | 0.35 | 0.01 | 0.72 | 0.13 | 0.72 | 0.67 | 0.9 | 0.92 |
| vervet | 0 | 0 | 0.19 | 0 | 0.69 | 0.09 | 0.74 | 0.49 | 0.85 | 0.86 |
| baboon | 0 | 0 | 0.33 | 0.02 | 0.65 | 0.04 | 0.7 | 0.32 | 0.89 | 0.86 |
| macaque | 0.06 | 0.01 | 0 | 0 | 0.65 | 0.06 | 0.7 | 0.38 | 0.89 | 0.83 |
| baboon - macaque | 0 | 0 | 0 | 0 | 0 | 0 | 0.4 | 0.03 | 0.72 | 0.13 |
| vervet - baboon | 0 | 0 | 0 | 0 | 0.06 | 0.01 | 0.63 | 0.17 | 0.78 | 0.55 |
| colobus monkey - vervet | 0.06 | 0.03 | 0.6 | 0.05 | 0.71 | 0.34 | 0.84 | 0.81 | 0.93 | 0.94 |
| human - colobus monkey | 0 | 0 | 0 | 0 | 0.44 | 0.14 | 0.83 | 0.62 | 0.91 | 0.9 |
| dusky titi | 0.05 | 0.03 | 0.58 | 0.08 | 0.78 | 0.43 | 0.9 | 0.9 | 0.93 | 0.95 |
| owl monkey | 0.52 | 0.01 | 0.61 | 0.05 | 0.68 | 0.28 | 0.87 | 0.86 | 0.95 | 0.94 |
| marmoset | 0.19 | 0.01 | 0.42 | 0.11 | 0.65 | 0.37 | 0.9 | 0.89 | 0.93 | 0.95 |
| squirrel monkey | 0.85 | 0.02 | 0.41 | 0.03 | 0.74 | 0.4 | 0.87 | 0.86 | 0.95 | 0.96 |
| marmoset - squirrel monkey | 0 | 0 | 0 | 0 | 0 | 0 | 0 | 0 | 0.5 | 0 |
| owl monkey - marmoset | 0 | 0 | 0.95 | 0.01 | 0 | 0 | 0.61 | 0.07 | 0.85 | 0.3 |
| dusky titi - owl monkey | 0.13 | 0.03 | 0.59 | 0.11 | 0.78 | 0.32 | 0.88 | 0.9 | 0.94 | 0.94 |
| human – dusky titi | 0.27 | 0.03 | 0.35 | 0.12 | 0.78 | 0.54 | 0.8 | 0.93 | 0.94 | 0.96 |
| mouse lemur | 0.16 | 0.03 | 0.47 | 0.29 | 0.81 | 0.83 | 0.93 | 0.95 | 0.96 | 0.97 |
| galago | 0.37 | 0.07 | 0.81 | 0.51 | 0.86 | 0.88 | 0.95 | 0.96 | 0.98 | 0.98 |
| mouse lemur - galago | 0 | 0 | 0.16 | 0.01 | 0.81 | 0.08 | 0.83 | 0.37 | 0.89 | 0.76 |

**Table S22.** Accuracy obtained by DivE for the prediction of lost elements of different lengths in each clade^1^ of the phylogeny, when the selction strength is given by ρ=0.3^-1^.

| 0.4^-1^ acc | **50 bp** | | **100 bp** | | **200 bp** | | **500 bp** | | **1000 bp** | |
| --- | --- | --- | --- | --- | --- | --- | --- | --- | --- | --- |
| **tree** | Pr | Re | Pr | Re | Pr | Re | Pr | Re | Pr | Re |
| human | 0 | 0 | 0 | 0 | 0 | 0 | 0.44 | 0.02 | 0.79 | 0.14 |
| chimp | 0 | 0 | 0 | 0 | 0.97 | 0.02 | 0.43 | 0.05 | 0.77 | 0.22 |
| human -chimp | 0 | 0 | 0 | 0 | 0 | 0 | 0.56 | 0.05 | 0.73 | 0.3 |
| orangutan | 0 | 0 | 0 | 0 | 0.76 | 0.03 | 0.81 | 0.2 | 0.86 | 0.59 |
| human -orangutan | 0 | 0 | 0 | 0 | 0.09 | 0.01 | 1 | 0.01 | 0.74 | 0.01 |
| gibbon | 0 | 0 | 0.02 | 0.01 | 0.36 | 0.08 | 0.88 | 0.38 | 0.92 | 0.75 |
| human - gibbon | 0 | 0 | 0 | 0 | 0.13 | 0.01 | 0.72 | 0.05 | 0.83 | 0.34 |
| colobus monkey | 0 | 0 | 0.13 | 0.01 | 0.56 | 0.03 | 0.64 | 0.23 | 0.83 | 0.66 |
| vervet | 0 | 0 | 0 | 0 | 0 | 0 | 0.68 | 0.11 | 0.85 | 0.49 |
| baboon | 0 | 0.01 | 0.13 | 0.01 | 0.93 | 0.01 | 0.8 | 0.1 | 0.83 | 0.38 |
| macaque | 0 | 0 | 0 | 0 | 0.53 | 0.02 | 0.29 | 0.07 | 0.89 | 0.35 |
| baboon - macaque | 0 | 0 | 0 | 0 | 0.02 | 0 | 0.11 | 0 | 0.68 | 0.03 |
| vervet - baboon | 0 | 0 | 0 | 0 | 0 | 0 | 0.81 | 0.02 | 0.77 | 0.15 |
| colobus monkey - vervet | 0.17 | 0 | 0.69 | 0.04 | 0.81 | 0.01 | 0.8 | 0.41 | 0.92 | 0.78 |
| human - colobus monkey | 0 | 0 | 0.17 | 0.01 | 0.63 | 0.02 | 0.88 | 0.19 | 0.88 | 0.66 |
| dusky titi | 0 | 0 | 0.98 | 0.02 | 0.67 | 0.06 | 0.68 | 0.58 | 0.9 | 0.88 |
| owl monkey | 0.4 | 0.01 | 0.44 | 0.01 | 0.64 | 0.04 | 0.78 | 0.42 | 0.92 | 0.82 |
| marmoset | 0 | 0 | 0 | 0 | 0.57 | 0.08 | 0.75 | 0.5 | 0.93 | 0.87 |
| squirrel monkey | 0 | 0 | 0.06 | 0.02 | 0.89 | 0.1 | 0.79 | 0.5 | 0.89 | 0.87 |
| marmoset - squirrel monkey | 0 | 0 | 0 | 0 | 0 | 0 | 0.04 | 0 | 0.14 | 0.01 |
| owl monkey - marmoset | 0 | 0 | 0.13 | 0 | 0 | 0 | 0.39 | 0.03 | 0.59 | 0.07 |
| dusky titi - owl monkey | 0.18 | 0.01 | 0 | 0 | 0.26 | 0.07 | 0.87 | 0.5 | 0.9 | 0.87 |
| human – dusky titi | 0 | 0 | 0.34 | 0.02 | 0.59 | 0.13 | 0.81 | 0.7 | 0.86 | 0.92 |
| mouse lemur | 0 | 0 | 0.58 | 0.07 | 0.68 | 0.3 | 0.92 | 0.85 | 0.94 | 0.93 |
| galago | 0.74 | 0.01 | 0.45 | 0.15 | 0.88 | 0.55 | 0.93 | 0.9 | 0.96 | 0.95 |
| mouse lemur - galago | 0 | 0 | 0 | 0 | 0 | 0 | 0.73 | 0.1 | 0.89 | 0.41 |

**Table S23.** Accuracy obtained by DivE for the prediction of lost elements of different lengths in each clade^1^ of the phylogeny, when the selction strength is given by ρ=0.4^-1^.

| 0.5^-1^ acc | **50 bp** | | **100 bp** | | **200 bp** | | **500 bp** | | **1000 bp** | |
| --- | --- | --- | --- | --- | --- | --- | --- | --- | --- | --- |
| **tree** | Pr | Re | Pr | Re | Pr | Re | Pr | Re | Pr | Re |
| human | 0 | 0 | 0 | 0 | 0.01 | 0.01 | 0 | 0 | 0.49 | 0.02 |
| chimp | 0 | 0 | 0 | 0 | 0 | 0 | 0.07 | 0 | 0.24 | 0.04 |
| human -chimp | 0 | 0 | 0 | 0 | 0 | 0 | 1 | 0.01 | 0.85 | 0.1 |
| orangutan | 0 | 0 | 0 | 0 | 1 | 0.01 | 0.74 | 0.03 | 0.47 | 0.19 |
| human -orangutan | 0 | 0 | 0 | 0 | 0 | 0 | 0 | 0 | 0 | 0 |
| gibbon | 0 | 0 | 0 | 0 | 0 | 0 | 0.78 | 0.13 | 0.89 | 0.31 |
| human - gibbon | 0 | 0 | 0 | 0 | 0 | 0 | 0.5 | 0.01 | 0.72 | 0.05 |
| colobus monkey | 0 | 0 | 0 | 0 | 0.16 | 0.01 | 0.43 | 0.04 | 0.79 | 0.24 |
| vervet | 0 | 0 | 0 | 0 | 0 | 0 | 0.18 | 0.02 | 0.66 | 0.1 |
| baboon | 0 | 0 | 0 | 0 | 0 | 0 | 0.86 | 0.01 | 0.9 | 0.08 |
| macaque | 0.01 | 0.02 | 0 | 0 | 0 | 0 | 0.69 | 0.02 | 0.77 | 0.09 |
| baboon - macaque | 0 | 0 | 0 | 0 | 0 | 0 | 0 | 0 | 0.67 | 0.02 |
| vervet - baboon | 0 | 0 | 0 | 0 | 0 | 0 | 0.14 | 0 | 0.55 | 0.01 |
| colobus monkey - vervet | 0 | 0 | 0 | 0 | 0.29 | 0.01 | 0.87 | 0.15 | 0.89 | 0.39 |
| human - colobus monkey | 0 | 0 | 0 | 0 | 0 | 0 | 0.7 | 0.04 | 0.87 | 0.2 |
| dusky titi | 0 | 0 | 0 | 0 | 0.06 | 0.02 | 0.77 | 0.22 | 0.91 | 0.49 |
| owl monkey | 0 | 0 | 0 | 0 | 0.25 | 0.02 | 0.73 | 0.12 | 0.88 | 0.34 |
| marmoset | 0 | 0 | 0 | 0 | 0 | 0 | 0.87 | 0.17 | 0.79 | 0.5 |
| squirrel monkey | 0 | 0 | 0.13 | 0 | 0.64 | 0.03 | 0.87 | 0.19 | 0.88 | 0.49 |
| marmoset - squirrel monkey | 0 | 0 | 0 | 0 | 0 | 0 | 0 | 0 | 0 | 0 |
| owl monkey - marmoset | 0 | 0 | 0 | 0 | 0 | 0 | 0 | 0 | 0.24 | 0 |
| dusky titi - owl monkey | 0 | 0 | 0 | 0 | 0.25 | 0.01 | 0.61 | 0.09 | 0.81 | 0.54 |
| human – dusky titi | 0 | 0 | 0 | 0 | 0.72 | 0.03 | 0.72 | 0.27 | 0.9 | 0.67 |
| mouse lemur | 0.86 | 0.01 | 1 | 0.01 | 0.62 | 0.04 | 0.91 | 0.49 | 0.93 | 0.8 |
| galago | 0.05 | 0.01 | 0.75 | 0.04 | 0.91 | 0.15 | 0.89 | 0.61 | 0.95 | 0.89 |
| mouse lemur - galago | 0 | 0 | 0 | 0 | 0.32 | 0.01 | 0.61 | 0.03 | 0.73 | 0.14 |

**Table S24.** Accuracy obtained by DivE for the prediction of lost elements of different lengths in each clade[[1]](#footnote-2) of the phylogeny, when the selction strength is given by ρ=0.01^-1^.

| **Species** | **Sequence length** | **% of conserved bp** | **% of accelerated bp** |
| --- | --- | --- | --- |
| human | 29,955,247 bp | 12.56% | 15.97% |
| chimp | 28,449,864 bp | 20.11% | 9.60% |
| orangutan | 27,704,452 bp | 20.99% | 9.29% |
| gibbon | 26,493,669 bp | 21.97% | 8.13% |
| colobus monkey | 25,227,543 bp | 25.86% | 7.46% |
| vervet | 25,725,560 bp | 28.62% | 7.67% |
| baboon | 26,034,723 bp | 30.01% | 8.54% |
| macaque | 25,735,338 bp | 30.31% | 8.51% |
| dusky titi | 23,254,212 bp | 16.35% | 6.65% |
| owl monkey | 23,254,994 bp | 16.12% | 6.97% |
| marmoset | 23,449,969 bp | 15.73% | 7.29% |
| squirrel monkey | 22,091,666 bp | 15.64% | 7.30% |
| mouse lemur | 17,492,420 bp | 14.53% | 2.07% |
| galago | 17,437,102 bp | 14.23% | 2.22% |

**Table S25**. Percentage of ENCODE primate sequences predicted to be either conserved or accelerated by DivE.

| **GO Term** | **Description** | **Genes** |
| --- | --- | --- |
|  | **biological process** |  |
| GO:0002227 | innate immune response in mucosa | APOA4 |
| GO:0006869 | lipid transport | APOA4 |
| GO:0006982 | response to lipid hydroperoxide | APOA4 |
| GO:0007159 | leukocyte cell-cell adhesion | APOA4 |
| GO:0008203 | cholesterol metabolic process | APOA4 |
| GO:0010873 | positive regulation of cholesterol esterification | APOA4 |
| GO:0010898 | positive regulation of triglyceride catabolic process | APOA4 |
| GO:0019430 | removal of superoxide radicals | APOA4 |
| GO:0030300 | regulation of intestinal cholesterol absorption | APOA4 |
| GO:0032374 | regulation of cholesterol transport | APOA4 |
| GO:0033344 | cholesterol efflux | APOA4 |
| GO:0033700 | phospholipid efflux | APOA4 |
| GO:0034371 | chylomicron remodeling | APOA4 |
| GO:0034372 | very-low-density lipoprotein particle remodeling | APOA4 |
| GO:0034375 | high-density lipoprotein particle remodeling | APOA4 |
| GO:0034378 | chylomicron assembly | APOA4 |
| GO:0034445 | negative regulation of plasma lipoprotein oxidation | APOA4 |
| GO:0042157 | lipoprotein metabolic process | APOA4 |
| GO:0042632 | cholesterol homeostasis | APOA4 |
| GO:0042744 | hydrogen peroxide catabolic process | APOA4 |
| GO:0043691 | reverse cholesterol transport | APOA4 |
| GO:0044240 | multicellular organismal lipid catabolic process | APOA4 |
| GO:0045723 | positive regulation of fatty acid biosynthetic process | APOA4 |
| GO:0046470 | phosphatidylcholine metabolic process | APOA4 |
| GO:0051006 | positive regulation of lipoprotein lipase activity | APOA4 |
| GO:0055088 | lipid homeostasis | APOA4 |
| GO:0065005 | protein-lipid complex assembly | APOA4 |
| GO:0007162 | negative regulation of cell adhesion | ARHGDIG |
| GO:0007264 | small GTPase mediated signal transduction | ARHGDIG |
| GO:0007266 | Rho protein signal transduction | ARHGDIG |
| GO:0051056 | regulation of small GTPase mediated signal transduction | ARHGDIG |
| GO:0008150 | CGN Depdc5 Lca5l |  |
| GO:0035556 | intracellular signal transduction | Depdc5 |
| GO:0002690 | positive regulation of leukocyte chemotaxis | F7 |
| GO:0006508 | proteolysis | F7 |
| GO:0006916 | anti-apoptosis | F7 |
| GO:0007598 | blood coagulation, extrinsic pathway | F7 |
| GO:0007623 | circadian rhythm | F7 |
| GO:0009725 | response to hormone stimulus | F7 |
| GO:0010641 | positive regulation of platelet-derived growth factor receptor signaling pathway | F7 |
| GO:0014070 | response to organic cyclic substance | F7 |
| GO:0017187 | peptidyl-glutamic acid carboxylation | F7 |
| GO:0030194 | positive regulation of blood coagulation | F7 |
| GO:0030335 | positive regulation of cell migration | F7 |
| GO:0031100 | organ regeneration | F7 |
| GO:0031667 | response to nutrient levels | F7 |
| GO:0032571 | response to vitamin K | F7 |
| GO:0043627 | response to estrogen stimulus | F7 |
| GO:0043687 | post-translational protein modification | F7 |
| GO:0044267 | cellular protein metabolic process | F7 |
| GO:0050927 | positive regulation of positive chemotaxis | F7 |
| GO:0051897 | positive regulation of protein kinase B signaling cascade | F7 |
| GO:0060416 | response to growth hormone stimulus | F7 |
| GO:0006953 | acute-phase response | F8 |
| GO:0007155 | cell adhesion | F8 |
| GO:0055114 | oxidation-reduction process | F8 |
| GO:0007596 | blood coagulation | F8 F7 |
| GO:0015671 | oxygen transport | HBQ1 |
| GO:0006810 | transport | HBQ1 HBZ |
| GO:0000122 | negative regulation of transcription from RNA polymerase II promoter | HBZ |
| GO:0043249 | erythrocyte maturation | HBZ |
| GO:0007165 | signal transduction | LILRB4 |
| GO:0006952 | defense response | LILRB5 |
| GO:0007166 | cell surface receptor linked signaling pathway | LILRB5 |
| GO:0015870 | acetylcholine transport | SERPINB7 |
| GO:0006641 | triglyceride metabolic process | SLC22A4 |
| GO:0006811 | ion transport | SLC22A4 |
| GO:0006814 | sodium ion transport | SLC22A4 |
| GO:0007589 | body fluid secretion | SLC22A4 |
| GO:0009437 | carnitine metabolic process | SLC22A4 |
| GO:0015695 | organic cation transport | SLC22A4 |
| GO:0015697 | quaternary ammonium group transport | SLC22A4 |
| GO:0015879 | carnitine transport | SLC22A4 |
| GO:0055085 | transmembrane transport | SLC22A4 |
| GO:0006631 | fatty acid metabolic process | UGT1A8 |
| GO:0006805 | xenobiotic metabolic process | UGT1A8 |
| GO:0008152 | metabolic process | UGT1A8 |
| GO:0008202 | steroid metabolic process | UGT1A8 |
| GO:0009804 | coumarin metabolic process | UGT1A8 |
| GO:0009812 | flavonoid metabolic process | UGT1A8 |
| GO:0017144 | drug metabolic process | UGT1A8 |
| GO:0031324 | negative regulation of cellular metabolic process | UGT1A8 |
| GO:0042573 | retinoic acid metabolic process | UGT1A8 |
| GO:0045922 | negative regulation of fatty acid metabolic process | UGT1A8 |
| GO:0045939 | negative regulation of steroid metabolic process | UGT1A8 |
|  | **cellular component** |  |
| GO:0009986 | cell surface | APOA4 |
| GO:0034361 | very-low-density lipoprotein particle | APOA4 |
| GO:0034364 | high-density lipoprotein particle | APOA4 |
| GO:0042627 | chylomicron | APOA4 |
| GO:0005788 | endoplasmic reticulum lumen | APOA4 F7 |
| GO:0005576 | extracellular region | APOA4 F8 F7 |
| GO:0005829 | cytosol | ARHGDIG |
| GO:0016023 | cytoplasmic membrane-bounded vesicle | ARHGDIG |
| GO:0005737 | cytoplasm | ARHGDIG SERPINB7 |
| GO:0005923 | tight junction | CGN |
| GO:0016459 | myosin complex | CGN |
| GO:0030054 | cell junction | CGN |
| GO:0005622 | intracellular | DDX43 |
| GO:0005575 | Depdc5 Lca5l |  |
| GO:0005796 | Golgi lumen | F7 |
| GO:0042598 | vesicular fraction | F7 |
| **GO:0005615** | extracellular space | F7 F8 APOA4 |
| GO:0031093 | platelet alpha granule lumen | F8 |
| GO:0005833 | hemoglobin complex | HBZ HBQ1 |
| **GO:0016021** | integral to membrane | LILRA4 LILRB4 LILRB5 SYT8 UGT1A8 |
| GO:0005886 | plasma membrane | LILRB4 SYT8 F8 F7 SLC22A4 |
| GO:0030529 | ribonucleoprotein complex | RPS9 |
| GO:0005739 | mitochondrion | SLC22A4 |
| GO:0005887 | integral to plasma membrane | SLC22A4 |
| GO:0016324 | apical plasma membrane | SLC22A4 |
| GO:0001669 | acrosomal vesicle | SYT8 |
| GO:0031410 | cytoplasmic vesicle | SYT8 |
| GO:0005783 | endoplasmic reticulum | UGT1A8 |
| GO:0005789 | endoplasmic reticulum membrane | UGT1A8 |
| GO:0005792 | microsome | UGT1A8 |
| GO:0016020 | membrane | UGT1A8 LILRA4 LILRB5 |
|  | **molecular function** |  |
| GO:0005319 | lipid transporter activity | APOA4 |
| GO:0008289 | lipid binding | APOA4 |
| GO:0016209 | antioxidant activity | APOA4 |
| GO:0017127 | cholesterol transporter activity | APOA4 |
| GO:0031210 | phosphatidylcholine binding | APOA4 |
| GO:0043499 | eukaryotic cell surface binding | APOA4 |
| GO:0060228 | phosphatidylcholine-sterol O-acyltransferase activator activity | APOA4 |
| GO:0042803 | protein homodimerization activity | APOA4 UGT1A8 |
| GO:0005094 | Rho GDP-dissociation inhibitor activity | ARHGDIG |
| GO:0005096 | GTPase activator activity | ARHGDIG |
| GO:0003774 | motor activity | CGN |
| GO:0003779 | actin binding | CGN |
| GO:0005515 | protein binding | CGN SLC22A4 F7 F8 |
| GO:0003723 | RNA binding | DDX43 |
| GO:0004004 | ATP-dependent RNA helicase activity | DDX43 |
| GO:0004386 | helicase activity | DDX43 |
| GO:0016787 | hydrolase activity | DDX43 |
| GO:0000166 | nucleotide binding | DDX43 SLC22A4 |
| GO:0005524 | ATP binding | DDX43 SLC22A4 |
| GO:0003674 | Depdc5 Lca5l |  |
| GO:0001948 | glycoprotein binding | F7 |
| GO:0004252 | serine-type endopeptidase activity | F7 |
| GO:0005102 | receptor binding | F7 |
| GO:0005509 | calcium ion binding | F7 |
| GO:0008233 | peptidase activity | F7 |
| GO:0008236 | serine-type peptidase activity | F7 |
| GO:0016491 | oxidoreductase activity | F8 |
| GO:0005507 | copper ion binding | F8 APOA4 |
| GO:0046872 | metal ion binding | F8 HBQ1 HBZ |
| GO:0005344 | oxygen transporter activity | HBQ1 HBZ |
| GO:0019825 | oxygen binding | HBQ1 HBZ |
| GO:0020037 | heme binding | HBQ1 HBZ |
| GO:0005488 | binding | HEATR7B1 |
| GO:0004872 | receptor activity | LILRA4 LILRB4 |
| GO:0003823 | antigen binding | LILRB4 |
| **GO:0004888** | transmembrane receptor activity | LILRB5 |
| GO:0004867 | serine-type endopeptidase inhibitor activity | SERPINB7 |
| GO:0030414 | peptidase inhibitor activity | SERPINB7 |
| GO:0005215 | transporter activity | SLC22A4 |
| GO:0008513 | secondary active organic cation transmembrane transporter activity | SLC22A4 |
| GO:0015075 | ion transmembrane transporter activity | SLC22A4 |
| GO:0015226 | carnitine transporter activity | SLC22A4 |
| GO:0015293 | symporter activity | SLC22A4 |
| GO:0015491 | cation:cation antiporter activity | SLC22A4 |
| GO:0015651 | quaternary ammonium group transmembrane transporter activity | SLC22A4 |
| GO:0030165 | PDZ domain binding | SLC22A4 |
| GO:0001972 | retinoic acid binding | UGT1A8 |
| GO:0004857 | enzyme inhibitor activity | UGT1A8 |
| GO:0005496 | steroid binding | UGT1A8 |
| GO:0005504 | fatty acid binding | UGT1A8 |
| GO:0008144 | drug binding | UGT1A8 |
| GO:0015020 | glucuronosyltransferase activity | UGT1A8 |
| GO:0019899 | enzyme binding | UGT1A8 |
| GO:0046982 | protein heterodimerization activity | UGT1A8 |

**Table S26.** GO categories associated with genes predicted to be under positive selection. GO categories are shown in bold if they have been previously found to be over-represented among mammalian genes predicted to be under positive selection in [1].

**References**

1. Kosiol C, Vinar T, da Fonseca RR, Hubisz MJ, Bustamante CD, Nielsen R, Siepel A: **Patterns of positive selection in six Mammalian genomes**. *PLoS Genet* 2008, **4**(8):e1000144.

1. The name of the clade (subtree) is given by the species name when there is only one node in that subtree, or by two species' names when the clade’s root is the most common ancestor of those two species. [↑](#footnote-ref-2)
